# Bifunctionality and Antitumor Efficacy of ZG-126, a Vitamin D Receptor Agonist/Histone Deacetylase Inhibitor Hybrid Molecule

**DOI:** 10.1021/acs.jmedchem.4c00706

**Published:** 2024-06-21

**Authors:** Fatemeh Sarmadi, Zhizhong Gao, Jie Su, Camille Barbier, Patricio Artusa, Krikor Bijian, James L. Gleason, John H. White

**Affiliations:** † Department of Physiology, McGill University, 3655 Promenade Sir William Osler, Montreal, QC H3G 1Y6, Canada; ‡ Department of Medicine, McGill University, 3655 Promenade Sir William Osler, Montreal, QC H3G 1Y6, Canada; § Department of Chemistry, McGill University, 801 Sherbrooke W., Montreal, QC H3A 0B8, Canada; ∥ Segal Cancer Center and Lady Davis Institute for Medical Research, 3755 Cote Ste-Catherine, Montreal, QC H3T 1E2, Canada

## Abstract

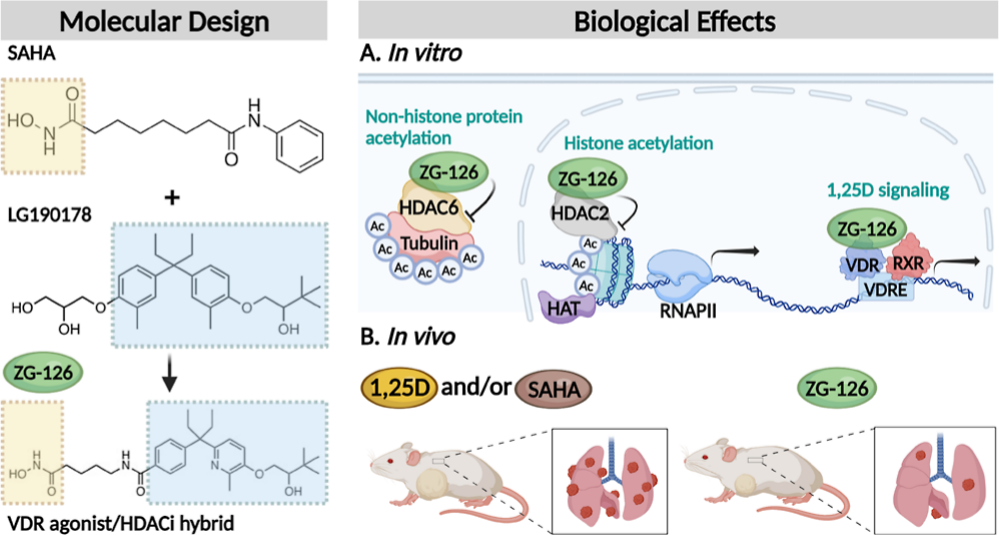

Analogues of hormonal vitamin D, 1,25-dihydroxyvitamin D (1,25D), signal through the nuclear vitamin D receptor (VDR). They have potential in combination therapies with other anticancer agents such as histone deacetylase inhibitors (HDACi’s). Here, we characterize the ZG series of hybrid compounds that combine HDACi within the backbone of a VDR agonist. All display improved solubility, with ZG-126 being the most robustly bifunctional molecule in multiple cell lines. ZG-126 is well tolerated and strongly induces VDR target gene expression in vivo at therapeutic doses. Its antitumor efficacy is superior to 1,25D and the HDACi SAHA, separately or together, in mouse models of melanoma and triple-negative breast cancer (TNBC). Notably, ZG-126 treatment reduces metastases almost 4-fold in an aggressive TNBC model. ZG-126 also reduces total macrophage infiltration and the proportion of immunosuppressive M2-polarized macrophages in TNBC tumors by 2-fold. ZG-126 thus represents a bifunctional and efficacious anticancer agent with improved physicochemical properties.

## Introduction

**Figure 1:**
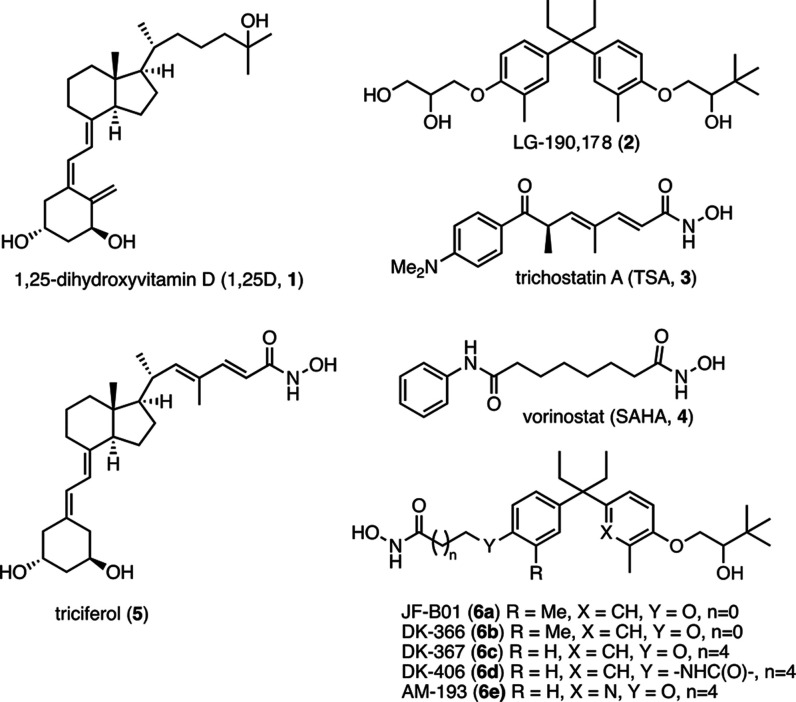
Structures of 1,25D (1); a nonsecosteroidal VDR agonist LG190178 (2); HDACi’s TSA (3) and SAHA (4); and the original secosteroidal hybrid compound triciferol (5). The structure of nonsecosteroidal hybrids JFB01, DK366, DK-367, DK-406, and AM-193 (6a–e) is also shown.

Although initially identified as a cure for nutritional rickets,^1^ a disease of bone growth, vitamin D has attracted extensive interest because of its “non-classical” actions.^2^ Apart from supplementation, vitamin D is obtained from limited dietary sources or from cutaneous exposure to adequate solar ultraviolet B (UVB) irradiation, which induces the photochemical and thermal conversion of the cholesterol precursor, 7-dehydrocholesterol. This cleaves the steroid B ring and generates secosteroidal vitamin D_3_ (cholecalciferol). The active form of vitamin D, 1,25-dihydroxyvitamin D (1,25D, calcitriol; **1**, Figure 1), is produced via sequential hydroxylations, first at the 25 position (25-hydroxyvitamin D, calcidiol) and then in a highly regulated, tissue-specific manner at the 1α position by the enzyme CYP27B1.^2,3^ 1,25D binds to and activates the VDR (vitamin D receptor), a protein that is widely expressed, including in several tissues unrelated to calcium homeostasis. The VDR functions as a ligand-regulated transcription factor and binds to regulatory regions of genes controlling cell cycle regulation, differentiation, and immune function.^3−5^ Notably, there is evidence from clinical data that vitamin D supplementation can prevent cancer.^6^

1,25D analogues have been investigated for their potential in treatment of proliferative disorders, such as cancer and psoriasis.^7^ Indeed, 1,25D and its analogues were antiproliferative in several cancer models, including breast cancer.^8^ In previous work, we found that a secosteroidal 1,25D analogue, EB1089, was efficacious in a head and neck squamous carcinoma (HNSCC) model.^9^ Unfortunately, typical of other cancers, there are also multiple HNSCC models that are resistant to 1,25D.^10^ Consequently, 1,25D analogues have generally failed as monotherapies because of acquired tumor resistance. Importantly, however, vitamin D signaling is often retained in resistant cells, underlying the potential for 1,25D and its analogues in combination therapies.^11^ We and others have found that 1,25D and HDACi’s act synergistically in 1,25D-resistant models.^12−16^ In addition, VDR signaling and the HDACi sodium butyrate cooperate to induce colon cancer cell differentiation.^17^ In the nucleus, HDACs control the acetylation of histones, transcription factors, and cofactors and thus regulate gene transcription. However, they also control the acetylation of other non-nuclear proteins such as tubulin and HSP90.^18−22^ A prototypical HDACi trichostatin A (TSA; **3**, Figure 1), initially isolated for its antifungal activity, also has potent antiproliferative properties. Class I, II, and IV HDACs are zinc metalloenzymes, and TSA is a typical HDACi in that it is composed of a zinc-chelating group (frequently a hydroxamic acid) flanked by a linker and a cap group, which can be highly heterogeneous in structure.^23^ Several HDACi’s, including SAHA (vorinostat; **4**, Figure 1), have been approved for clinical use for cutaneous and peripheral T-cell lymphoma and multiple myeloma,^24^ or are in clinical trials.^20,25^

We have been developing fully integrated bifunctional hybrid molecules that combine VDR agonism with HDACi activity within the backbone of a VDR agonist. They thus represent a form of combination therapy, now the norm in the treatment of cancer, as they regulate distinct but biochemically complementary targets. Initially, we developed triciferol (**5**, Figure 1)^26^ and other secosteroidal hybrids^27^ by replacing the cholesterol-like 1,25D side chain of **1** with the zinc-chelating dienylhydroxamic acid unit of TSA. The hydroxamic acid successfully mimics the 1,25D 25-OH group, while the secosteroid functions as an HDACi cap group. As the synthesis of secosteroidal hybrids required numerous (>25) steps, HDACi activity was then incorporated into the more easily assembled diarylpentane 1,25D analogue LG190178 (**2**; Figure 1), resulting in hybrid JF-B01 (**6a**, Figure 1). Receptor binding of JF-B01 and its analogues was confirmed by fluorescence polarization assays and crystal structures of nonsecosteroidal hybrid/VDR complexes.^28^ Subsequently, we improved the HDACi potency by removal of the methyl group in the aromatic ring adjacent to the HDACi group and lengthening of the side chain^29^ [see DK-366 (**6b**), -367 (**6c**), and -406 (**6d**); Figure 1].

Hybrids were bioavailable and inhibited tumor growth in the mouse 4T1 TNBC model, under conditions where 1,25D and SAHA given alone or in combination were not efficacious.^30^ 4T1 cells are derived from a TNBC cell line in BALB/c mice, form rapidly growing primary tumors, and are aggressively metastatic.^31^ TNBC,^32^ by definition, lacks the markers of other forms of breast cancer (estrogen and progesterone receptors or amplified epidermal growth factor receptor HER2), and represents about 15–20% of all breast malignancies. While results from the 4T1 model were encouraging, the hydrophobic diarylpentane core made solubilizing DK-367 and -406 problematic. Therefore, we replaced one of the benzene rings by a pyridine (e.g., AM-193, **6e**; Figure 1),^33^ which did not substantially compromise the bifunctionality. However, gains in the solubility of AM-193 relative to DK-367, its closest structural analogue, were relatively modest, and, as described below, the efficacy of AM-193 in vivo is limited by its relative toxicity. To further improve the solubility, we have replaced the ether group in the hydroxamic side chain of AM-193 with a more polar amide group, producing the ZG series of hybrids. Here, we show that the ZG compounds are bifunctional and are more soluble and substantially better tolerated in vivo than AM-193. We determined the IC50 values of ZG-126 for all 11 Class I, II, and IV HDACs and found that it induces robust VDR target gene expression in vivo. Moreover, we show that ZG-126 displays efficacious antitumor and antimetastatic activity in mouse models of melanoma and TNBC, both of which are malignancies in need of efficacious therapeutics.

## Results and Discussion

Previously developed hybrids DK-366, DK-367, and DK-406 (Figure 1), with their diarylpentane cores, were found to be bifunctional in vitro, and DK-366 and -406, further tested in vivo, were bioavailable and efficacious in reducing the tumor burden and the number of metastases in the 4T1 TNBC model.^30^ However, the efficacy of DK-406 was limited by its relatively narrow therapeutic window,^30^ and its hydrophobic diarylpentane core limited its solubility.^33^ Pyridyl-substituted AM-193 (**6e**, Figure 1),^33^ with a side-chain ether linker, is bifunctional and moderately more soluble than its closest analogue DK-367, but still less soluble than DK-406, which bears an amide in its side chain. Moreover, AM-193 use is limited by its toxicity in vivo (see Figure 6). To optimize the solubility, we combined the pyridine core and amide linkage into a new set of hybrids, ZG-102, -126, and -132, which differ in their HDACi side-chain lengths.

### Synthesis of ZG Compounds

**Scheme 1:**
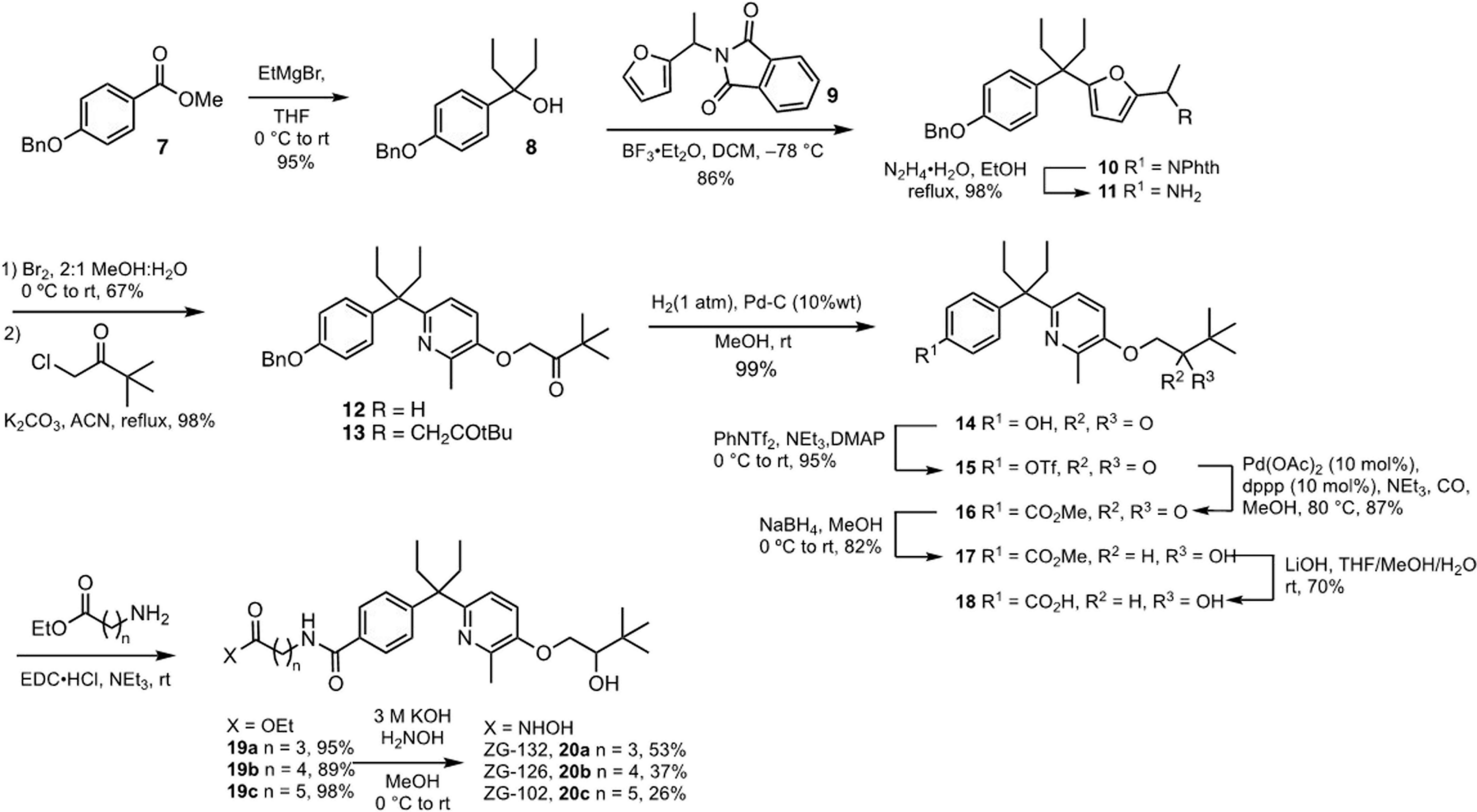
Synthesis of ZG-Series Hybrids

The synthesis of ZG series (Scheme 1) was performed in the route developed for AM compounds featuring a key aza-Achmatowicz rearrangement.^33^ The precursor for the rearrangement was prepared by the addition of ethyl magnesium bromide to methyl 4-benzyloxybenzoate, in 95% yield, followed by Friedel–Crafts alkylation with phthalimide-protected furylamine **9** in the presence of BF_3_·OEt_2_ to afford diarylpentane **10** in 86% yield. The phthalimide protecting group in **10** was removed, in 98% yield using hydrazine, to generate free amine **11**, which is the desired precursor for the aza-Achmatowicz rearrangement. The original Achmatowicz conditions^34^ were applied, using Br_2_ in 2:1 MeOH/H_2_O, to establish the pyridine ring of intermediate **12** in 67% yield. The subsequent O-alkylation of 3-hydroxypyridine with 1-chloropinacolone afforded **13** in 98% yield. After hydrogenolysis of the benzyl group, the resulting phenol **14** was converted to a methyl ester **16** via a sequential triflation and palladium-catalyzed carbonylation in 83% yield. Reduction of the ketone in **16** with NaBH_4_, followed by ester saponification afforded acid **18** in 57% over two steps. With the carboxylic acid in hand, EDC·HCl-catalyzed amide coupling with C4, C5, and C6 amino esters afforded penultimate hybrid precursors **19a**–**c** in 89–98% yields, respectively. Finally, treatment with hydroxylamine and KOH afforded the desired hybrids ZG-132 (**20a**, *n* = 3), ZG-126 (**20b**, *n* = 4), and ZG-102 (**20c**, *n* = 5) in 26–53% yields.

### Solubility and Assessment of Bifunctionality In Vitro

**Figure 2:**
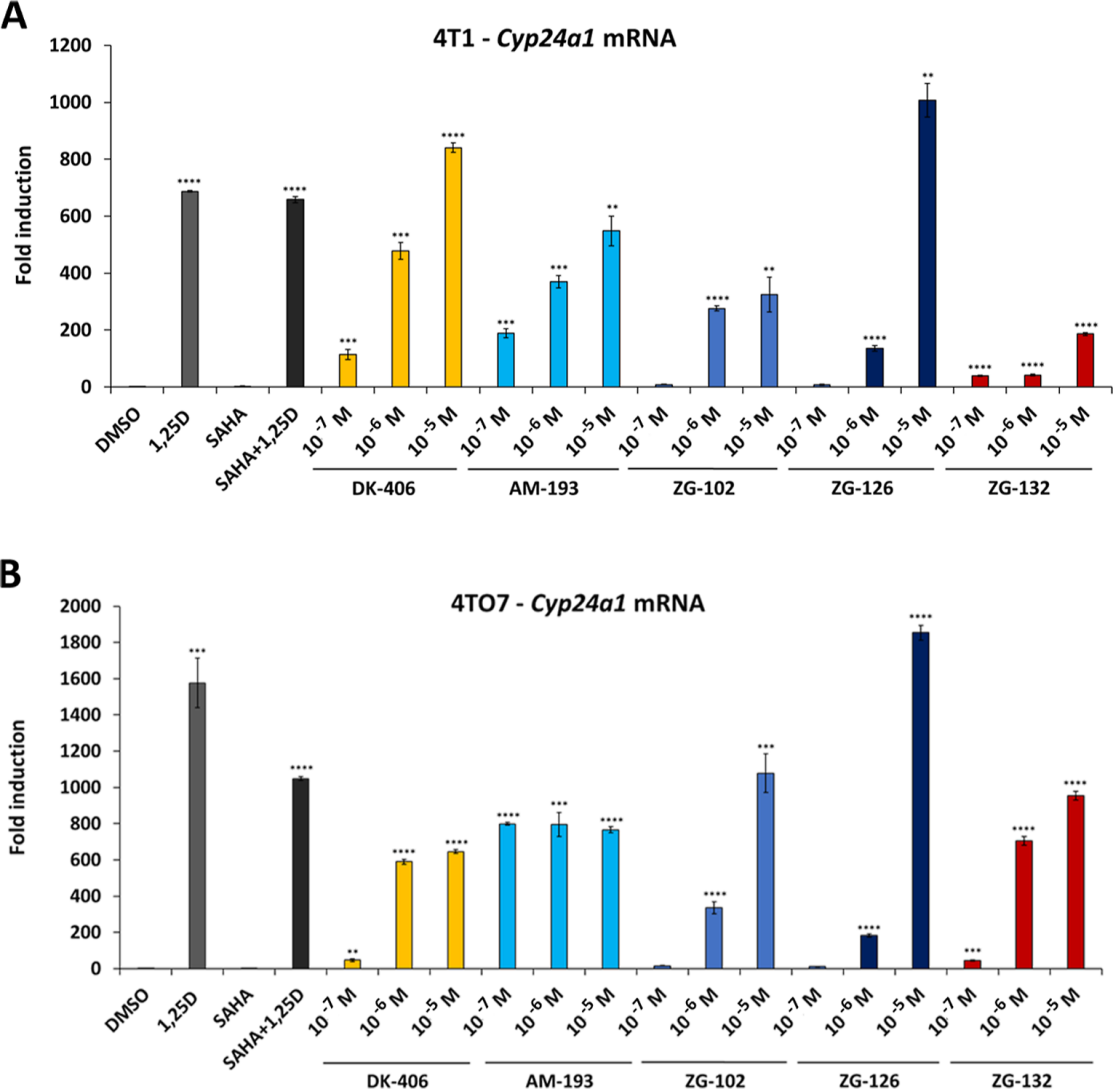
Bifunctionality of ZG compounds in vitro. Analysis of *Cyp24a1* induction by ZG compounds, along with DK-406, AM-193, 1,25D, and SAHA in 4T1 cells (A) and 4TO7 cells (B).

**Figure 3:**
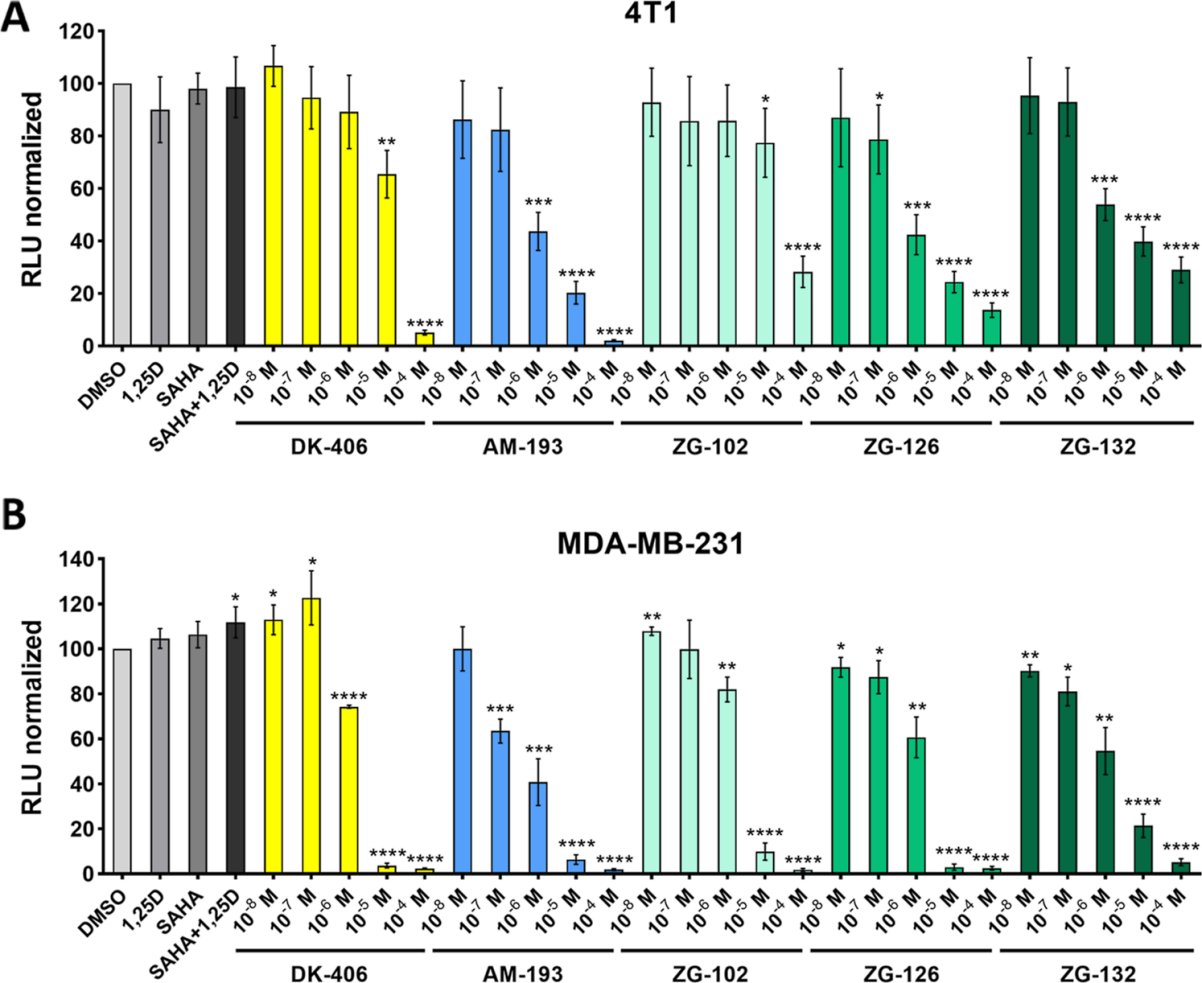
Assessment of cytotoxicity in mouse 4T1 cells (A) and the human TNBC cell line MDA-MB-231 (B) by the RealTime-Glo assay.

We first examined the effect of the modified structures on solubility. The incorporation of amide and pyridine groups in the hybrids improved the solubility of DK-367 (1.91 μg/mL) to 4.40 μg/mL (DK-406) and 2.43 μg/mL (AM-193), respectively. Combining these groups in the ZG hybrids further improved the solubility: 4.45, 9.19, and 10.71 μg/mL, respectively, for ZG-102, -126, and -132. The bifunctionality of ZG compounds was then analyzed in a battery of assays. VDR agonism was determined by evaluating the induction of the *Cyp24a1* gene. *Cyp24a1* encodes the enzyme that initiates the catabolic degradation of 1,25D in a negative-feedback loop, and its gene transcription is exquisitely sensitive to the presence of the agonist-bound VDR. Agonism was assessed initially in mouse 4T1 cells and in related 4TO7 TNBC cells (Figure 2A,B). ZG compounds induced *Cyp24a1* expression in both lines (although ZG-102 and ZG-132 did not appear to be a full agonist in 4T1 cells). Apparent potencies were lower than those of DK-406 and AM-193. However, half-maximal effective concentration (EC50) values could not be determined because of the toxicity of hybrids at concentrations above 10 μM (see Figure 3).

### Cytotoxicity of ZG Compounds In Vitro

Cytotoxicity in 4T1 cells was assessed using a RealTime-Glo cell viability assay, which measures the release of a modified *luciferase* substrate that can only be generated by viable cells. In these assays, the IC50 of ZG-126 was comparable to that of AM-193 (0.89 vs 0.74 μM; Figure 3A, see Table S1 for all IC50 values) and was 5–50-fold lower than those of ZG-132 and ZG-102, respectively (Figure 3A, Table S1). ZG-126 was also more potent than DK-406 (0.89 vs 9.08 μM; Figure 3A, Table S1). For these studies, we also examined human MDA-MB-231 TNBC cells. In these cells, IC50 values for ZG-126 and AM-193 were similar (0.92 vs 0.5 μM; Figure 3B, Table S1), whereas those of DK-406, ZG-102, and ZG-132 were 2.43, 2.18, and 0.95 μM, respectively. Note that in these assays, 1,25D and SAHA alone or in combination did not display significant cytotoxicity at saturating 100 nM^35^ concentrations (Figure 3A,B and data not shown).

**Figure 4:**
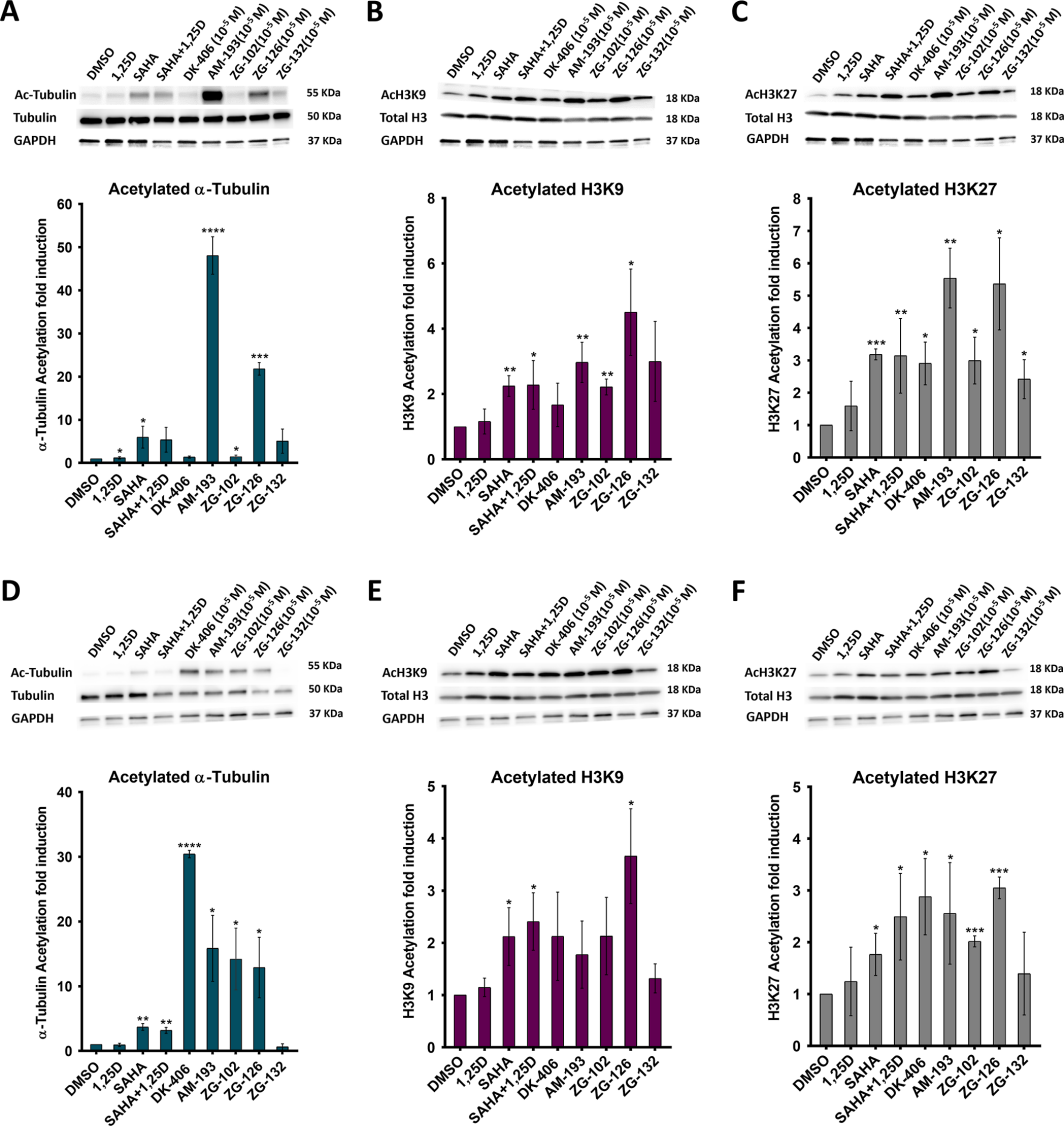
Evidence for HDAC inhibition in 4T1 and 4TO7 TNBC cells. Western blots and quantification of triplicate biological replicates analyzing the effects of 1,25D (100 nM), SAHA (100 nM), and hybrids (10 μM) on hyperacetylation of tubulin (A,D), H3K9 (B,E), and H3K27 (C,F) in 4T1 (A–C) and 4TO7 cells (D–F).

The potential for compounds as HDACi’s can be rapidly tested in cells in culture by monitoring the hyperacetylation of histone H3 and tubulin. H3 acetylation is controlled by multiple class I enzymes, and the class IIb enzyme HDAC6 regulates tubulin acetylation.^36−39^ In 4T1 cells, AM-193 (10 μM) is a highly efficacious inducer of tubulin acetylation. Of the ZG compounds, ZG-126 was by far the most efficacious (Figure 4A) at 10 μM and several-fold more efficacious than a saturating concentration of SAHA (100 nM)^35^ (Figure 4A). ZG-126 also performed well in inducing histone H3 hyperacetylation at both lysine 9 and 27 (H3K9 and H3K27; Figure 4B,C) in 4T1 cells, and was comparable or superior to AM-193. Similarly, in 4TO7 cells, ZG-126 induced higher levels of tubulin and histone hyperacetylation than SAHA and was as efficacious as or more efficacious than ZG-102 and ZG-132 (Figure 4D–F).

### IC50 Values for Inhibition of Purified HDACi’s 1–11 by ZG-126

**Figure 5:**
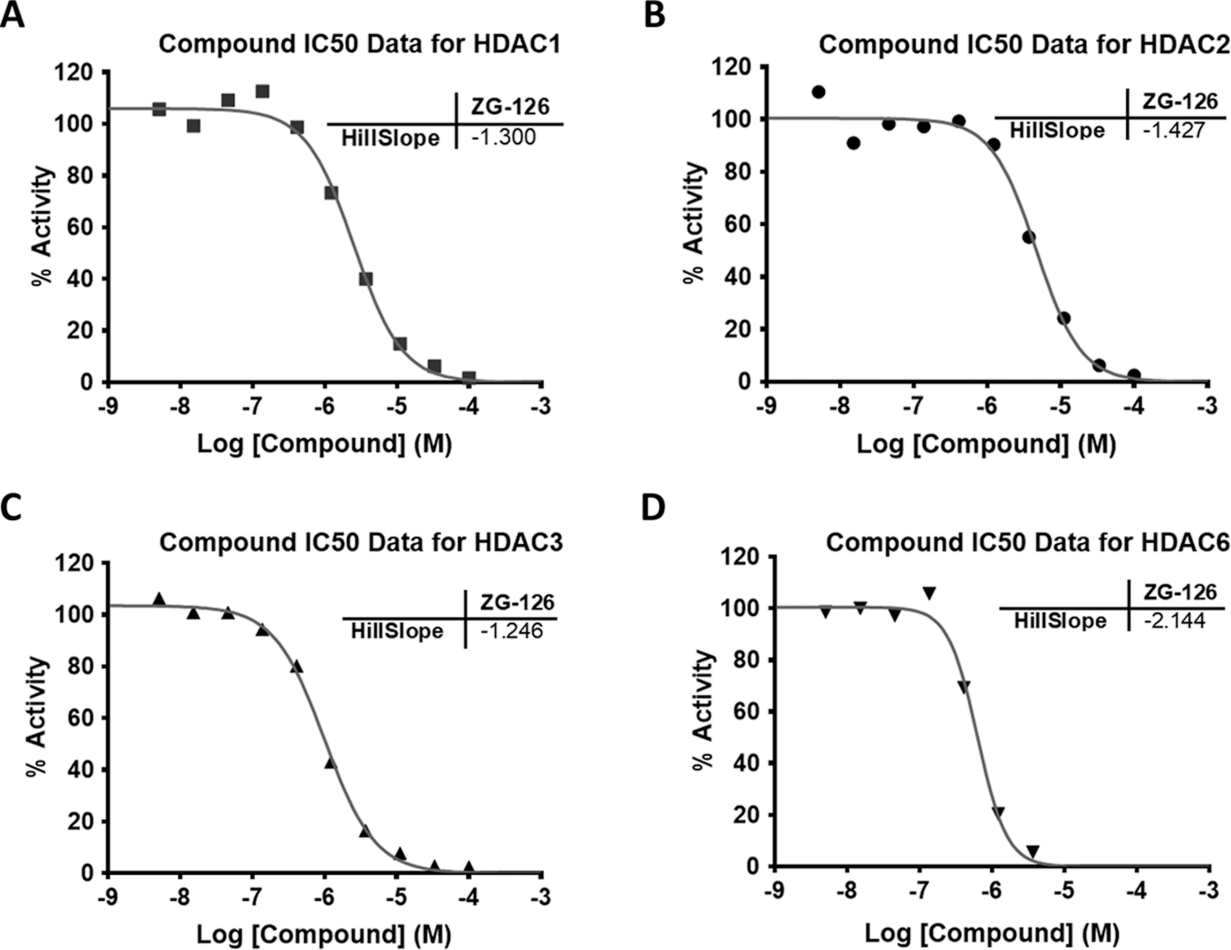
Dose–response profiles for the inhibition of HDAC1 (A), HDAC2 (B), HDAC3 (C), and HDAC6 (D) by ZG-126.

As ZG-126 showed the most favorable bifunctional profile of the ZG compounds, we assessed its HDAC inhibition further by biochemical assays. IC50 values were determined for all 11 class I, class II, or class IV HDACs; i.e., those (unlike sirtuins, class III) that are zinc metalloenzymes. TSA was used as a potent and nonselective inhibitor of class 1 enzymes (HDACs 1–3 and 8), class IIb enzyme HDAC 6, and class IV enzyme HDAC 11. The selective class II inhibitor TMP269 was used for class IIa enzymes HDACs 4, 5, 7, and 9, and quisinostat was used for the HDAC10 assay because it is a potent inhibitor of the enzyme.^40,41^ IC50 values for ZG-126 ranged by 100-fold across different isoforms, from 0.63 μM for HDAC6 to 68 μM for HDAC4 (Table 1). The outlier was HDAC9, where no or very limited inhibition was observed to concentrations as high as 100 μM. Raw data for HDACs 1–3 and HDAC6, chosen because they are key regulators of histone H3 acetylation^36,37,39^ and tubulin acetylation,^38^ respectively, are shown in Figure 5, whereas data for other HDACs are in Figure S1. The sub-μM IC50 for HDAC6 and low μM IC50 values for HDACs 1–3 are consistent with the capacity of ZG-126 to induce tubulin and histone H3 hyperacetylation in living cells. The IC50 of 4.6 μM of ZG-126 for inhibition of HDAC2 represents a modest improvement over that of AM-193 (7.2 μM) observed in previous studies,^33^ whereas the IC50 for HDAC6 inhibition was slightly higher (0.63 vs 0.3 μM). Taken together, these data suggest that, with the exception of HDAC9, ZG-126 acts as a pan-HDAC inhibitor of varying potency. However, data obtained with purified enzymes should be interpreted with care, as the enzymatic activity of HDACs can be modified allosterically by their association with multiple-protein complexes in vivo.^42,43^

**Table 1:** IC50 Values for Inhibition of Purified HDACs 1–11 by ZG-126

HDAC	compound name			
	ZG-126	trichostatin A	TMP269	quisinostat
HDAC1	2.53 × 10^–6^	2.05 × 10^–9^	ND	ND
HDAC2	4.64 × 10^–6^	4.73 × 10^–9^	ND	ND
HDAC3	9.97 × 10^–7^	1.75 × 10^–9^	ND	ND
HDAC4	6.76 × 10^–5^	ND	1.90 × 10^–7^	ND
HDAC5	5.94 × 10^–5^	ND	2.19 × 10^–7^	ND
HDAC6	6.33 × 10^–7^	1.02 × 10^–9^	ND	ND
HDAC7	5.54 × 10^–5^	ND	4.68 × 10^–8^	ND
HDAC8	3.31 × 10^–6^	3.38 × 10^–7^	ND	ND
HDAC9		ND	9.63 × 10^–9^	ND
HDAC10	1.39 × 10^–5^	ND	ND	6.75 × 10^–9^
HDAC11	2.02 × 10^–6^	3.59 × 10^–6^	ND	ND

### Bifunctionality of ZG-126 In Vitro in the B16-F10 Melanoma Model

ZG-126 was tested further in vitro, along with previously developed hybrids in mouse B16-F10 melanoma cells, derived from tumors in C57BL/6 mice.^44^ B16-F10 cells are responsive to 1,25D analogues^45^ and HDACi^46,47^ and are therefore widely used in preclinical studies of melanoma solid tumor growth and metastases.^48^ Although curable when treated early, melanoma is fatal when invasive. The American Cancer Society projected 97,610 new invasive melanoma cases in the US in 2023 and 7990 deaths. Current treatment options include chemotherapy, surgery, immunotherapy, or single agents targeting mutated genes such as B-RAF or C-KIT.^49^ We first characterized the bifunctionality of ZG-126 in B16-F10 cells. In contrast to 4T1 and 4TO7 cells, 1,25D and SAHA combined superinduced *Cyp24a1* expression in the B16-F10 model, an effect that was mimicked by ZG-126 treatment (Figure S2A). Unlike in TNBC cells (Figure 4), DK-406 induced very high levels of tubulin acetylation and histone hyperacetylation. Hyperacetylation observed in the presence of ZG-126 was comparable to or greater than that induced by a saturating concentration of SAHA (100 nM) (Figure S2B,C), and its cytotoxicity was similar to that of AM-193 (Figure S2D).

### Efficacy of ZG-126 In Vivo in the B16-F10 Melanoma Model

**Figure 6:**
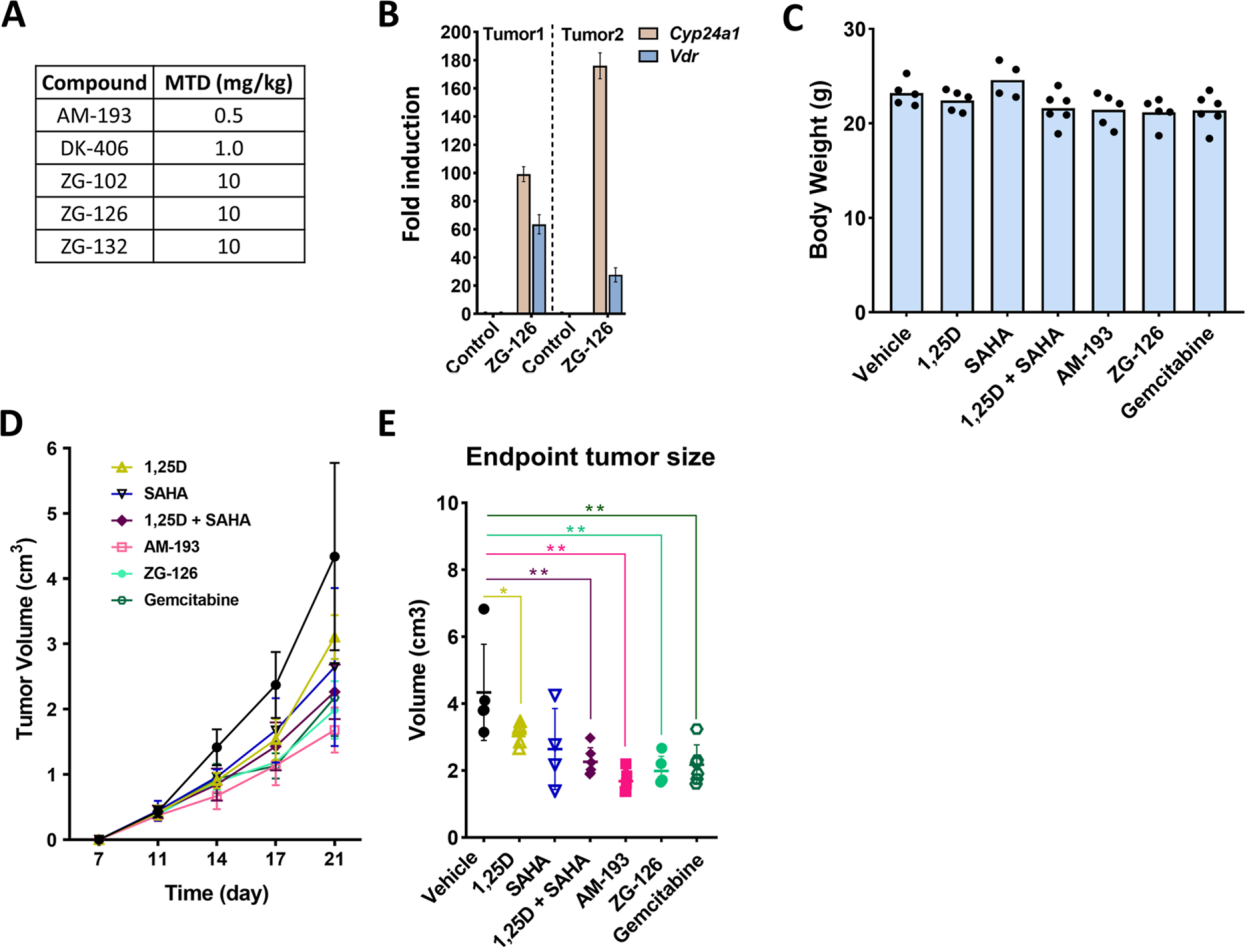
Efficacy of ZG-126 in vivo in the B16-F10 melanoma model. (A) Maximal tolerated daily doses of hybrids as indicated. (B) *Cyp24a1* induction in B16-F10 tumors treated with 2 doses of 5 mg/kg ZG-126, 24 h apart. (C) Body weights after 28 d. (D) Effects of 1,25D (0.25 mg/kg), SAHA (80 mg/kg), AM-193 (0.2 mg/kg), ZG-126 (5 mg/kg), or gemcitabine (50 mg/kg) on B16 tumor growth. (E) End-point tumor sizes from the study shown in (D).

The maximum tolerated dose (MTD) of hybrids was tested by daily administration to BALB/c mice. Under this dosing regimen, the MTD of DK-406 was limited to 1.0 mg/kg (Figure 6A), whereas AM-193 proved to be less well tolerated, with an MTD of 0.5 mg/kg. In contrast, the MTDs of all three ZG compounds were at least 10 mg/kg.

We were then interested in comparing the efficacy of ZG compounds and other hybrids in tumor models in vivo. We focused initially on the B16-F10 melanoma which is syngeneic with C57BL/6 mice. The efficacy of ZG-126 in vivo in this model was tested in two experiments. First, as IC50 values for several HDACs were in the sub- to low-μM range while the optimal induction of *Cyp24a1* (VDR agonism) in vitro was observed at 10 μM, it is important to determine if a potentially therapeutic dose of ZG-126 would induce VDR agonism in vivo. Thus, B16-F10 cells were implanted subcutaneously and, when tumors became palpable, were treated with two doses of ZG-126 (5 mg/kg) 24 h apart prior to harvesting RNA. Remarkably, in duplicate tumors from two animals, this led to robust induction of *Cyp24a1* as well as gene encoding the VDR itself, which is autoregulated (Figure 6B), confirming the VDR agonism of ZG-126 in vivo at a therapeutic dose. In a second experiment, we compared the antitumor efficacy of hybrids at ∼0.5 MTD doses. ZG-126 (5 mg/kg daily) and AM-193 (0.2 mg/kg daily) were tested along with 1,25D (0.25 μg/kg)^9^ and SAHA (80 mg/kg) alone or in combination, along with gemcitabine (50 mg/kg daily)^50^ as a positive control. All treatments were well tolerated, and body weights were unaffected (Figure 6C). Hybrids 1,25D and SAHA together and gemcitabine all produced a statistically significant reduction in tumor burden, with hybrids being at least as efficacious as the gemcitabine positive control (Figure 6D,E).

### Antitumor and Antimetastatic Efficacy of ZG-126 in the 4T1 TNBC Model

**Figure 7:**
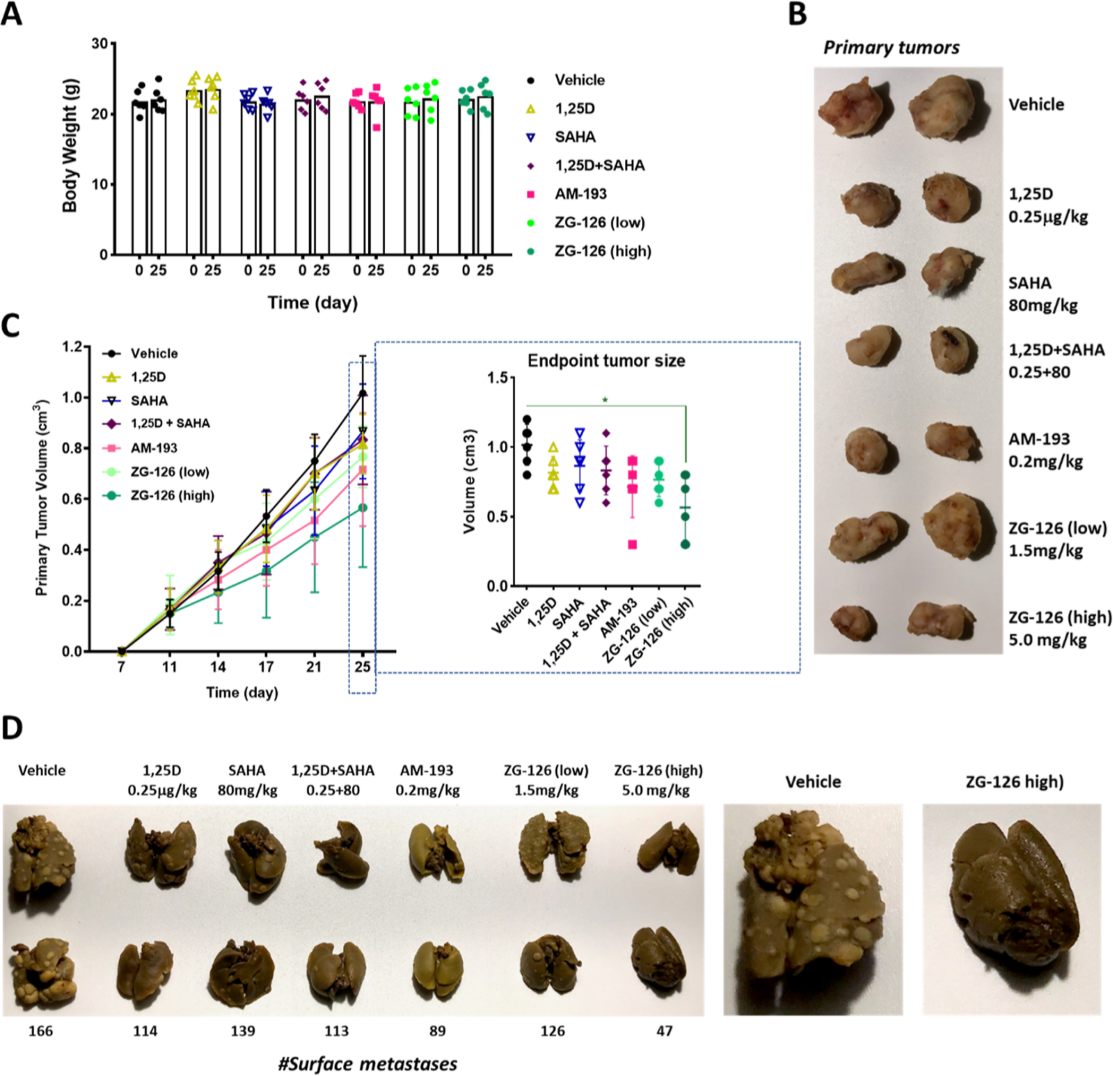
ZG-126 is an efficacious antimetastatic agent in the 4T1 TNBC model. (A) None of the treatments tested in the antitumor efficacy study induces weight loss in BALB/c mice. (B) Images of primary 4T1 tumors from control and treated mice. (C) Effects on 4T1 tumor growth in BALB/c mice treated as indicated. (D) Left: Images of lungs and numbers of lung surface metastases in BALB/c mice implanted with 4T1 cells. Right: Magnified images of lungs from animals treated with vehicle or high-dose ZG-126.

We then compared the efficacy of AM-193 at 0.2 mg/kg daily with that of ZG-126 at low and high doses (1.5 and 5 mg/kg, respectively) in the mouse 4T1 TNBC model, and with that of 1,25D (0.25 μg/kg) and SAHA (80 mg/kg) alone or in combination. As 4T1 cells are aggressively metastatic, we can also assess the antimetastatic activity in the same experiment. All treatments were well tolerated, and, as in the B16-F10 model, none induced weight loss or other signs of stress over the course of the experiment (Figure 7A). Treatments reduced the primary tumor burden to varying degrees. The combination of 1,25D and SAHA did not appear to be more efficacious than either compound alone, producing a nonsignificant reduction in the tumor volume of ∼20%. Notably, unlike in the B16-F10 model above, AM-193 (0.2 mg/kg) was less efficacious than the high dose of ZG-126 (5 mg/kg), which was the only treatment that produced a statistically significant reduction in tumor burden (∼50%; Figure 7B,C).

The antimetastatic activity was determined by counting the number of surface lung metastases of duplicate samples using a stereomicroscope. Here again, high-dose ZG-126 proved to be the most efficacious, substantially reducing the size and reducing the surface metastases by almost 4-fold (Figure 7D). This degree of efficacy is remarkable, as in previous experiments, DK-406 reduced the metastatic burden by ∼2-fold.^30^ The magnitude of the antimetastatic effect of ZG-126 in these experiments is comparable to those observed with other therapeutic agents, such as cisplatin,^51^ and suggests that it may be efficacious clinically in a neoadjuvant setting in reducing TNBC metastases. This result is significant because, in the clinic, compounds like ZG-126 would likely be used after surgical removal of the primary tumor.

### ZG-126 Reduces the Infiltration of Anti-inflammatory M2 Macrophages in 4T1 Tumors

**Figure 8:**
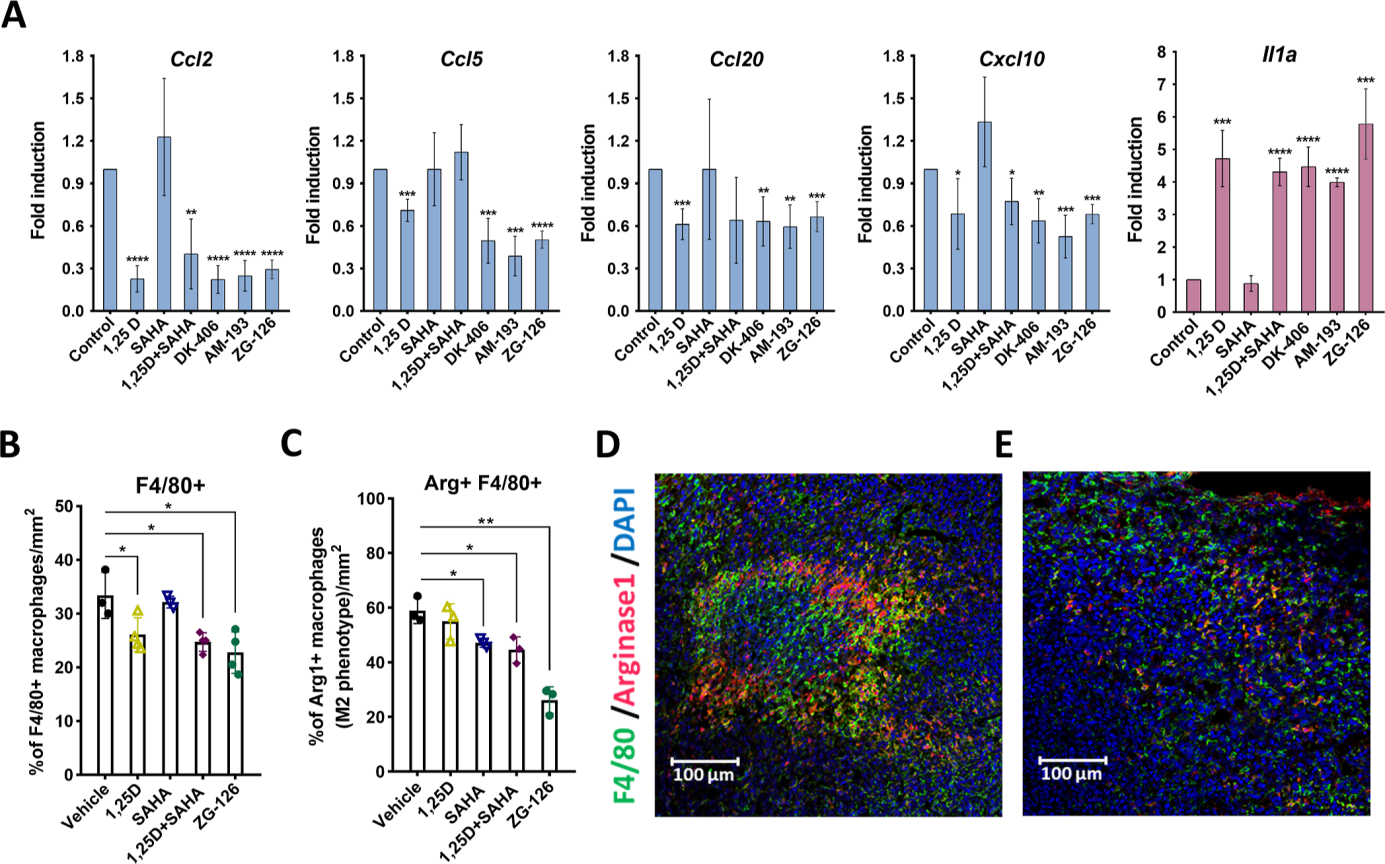
ZG-126 reduces the concentration of M2 macrophages in 4T1 tumors. (A) Gene expression profiles of cytokines and chemokines expressed in 4T1 cells treated as indicated in vitro. (B,C) Quantification of the density of macrophages as judged by F4/80 staining (B) and ratio of Arg1-positive macrophages to total macrophages (C). (D,E). Immunofluorescence analysis of 4T1 tumors stained for a macrophage marker F4/80 and an M2 macrophage-specific marker Arg1. Tumors from animals treated with vehicle (D) or ZG-126 (E) are shown. See Figure S3 for examples of tumors from other treated animals.

RNA-seq analysis performed in 4T1 cells in our previous study^33^ revealed that 1,25D and hybrids induced numerous changes in gene expression profiles. One of the signaling pathways enriched in treated cells was implicated in the regulation of myeloid cell infiltration into the tumor microenvironment (TME), which is particularly relevant to TNBC.^52^ Expression of genes controlling macrophage migration was downregulated in hybrid-treated cells compared to their untreated counterparts.^33^ Here, we analyzed the effect of ZG-126 on the regulation of the expression of genes implicated in the recruitment and polarization of macrophages. Tumor-associated macrophages (TAMs) are among the first to infiltrate and the most frequent immune cells in the TME.^53−55^ Cancer cells tend to secrete chemokines and cytokines that favor the differentiation of anti-inflammatory M2-polarized macrophages in the TME, which are immunosuppressive.^54^ These include *Ccl2*, *Ccl5*, *Ccl20,* and *Cxcl10*, all of which have been implicated in promoting breast tumor growth or metastasis^56−61^ and promoting the polarization of tumor-associated macrophages to an M2 anti-inflammatory phenotype.^62,63^ Treatment with either 1,25D or hybrids inhibited the expression of *Ccl2*, *Ccl5*, *Ccl20,* and *Cxcl10* (Figure 8A). In contrast, 1,25D and hybrids induced the expression of the gene encoding interleukin-1α (Figure 8A), a cytokine that polarizes macrophages to an inflammatory M1 phenotype.^64,65^

To further probe the effects of 1,25D and SAHA, alone or in combination, or ZG-126 on macrophage infiltration into 4T1 tumors, we performed an immunofluorescence (IF) imaging of tumor samples. Treatment with 1,25D or ZG-126, but not SAHA, significantly reduced total macrophage infiltration into 4T1 tumors, as measured by F4/80 staining (Figure 8B), consistent with effects of these compounds on gene expression. Moreover, ZG-126 treatment was the most efficacious at reducing the percentage of anti-inflammatory M2 macrophages, as measured by Arginase 1 (Arg1) staining (Figures 8C–E and S3). Thus, our results strongly suggest that ZG-126 induces the formation of a less immunosuppressive microenvironment in 4T1 tumors. This targeted effect raises the possibility that ZG-126 would be efficacious when combined with immunotherapeutics. For example, cotargeting TAMs with ZG-126 and phagocytosis checkpoint blockades^66,67^ may reshape the TME and enhance the reprogramming of TAMs toward a more tumoricidal phenotype. Notably, TAM-targeted strategies restore the antitumor effects of CD8+ T lymphocytes in TME by two means, first by suppressing the secretion of chemokines (CCL2, CCL18, and CCL22), cytokines (IL-4, IL-10, and TGFβ), and enzymes (COX2, MMPs, and ARG1) that negatively regulate T-cell cytotoxicity^68^ and second by suppressing signaling by TAM-expressed ligands such as CD80/CD86 and PD-L1, which attenuate immune responses.^69^ Inhibition of PD-L1 and CD80/CD86 is directly achieved through the immune checkpoint blockade of their receptors using anti-CTLA-4 and anti-PD-1 antibodies.^69,70^ Therefore, the combination of ZG-126 with immune checkpoint inhibitors could be an option to boost the immune function against tumor growth and metastasis.

## Conclusions

In summary, we have synthesized and tested in vitro and in vivo the ZG series of bifunctional VDR agonist/HDACi hybrid molecules. Replacing the nonpolar ether linker in the hydroxamic side chain of the AM series of hybrids^33^ with an amide substantially improved the solubility without sacrificing the bifunctionality. Indeed, ZG-126 is a full or superagonist of the VDR and generally induced robust tubulin or histone hyperacetylation in multiple cell lines. ZG compounds were substantially better tolerated in vivo than AM-193 or DK-406, and we provided evidence that ZG-126 can induce robust VDR target gene expression in vivo. A high-dose regimen of ZG-126 displayed antitumor activity in mouse melanoma and TNBC models along with impressive antimetastatic activity in the mouse 4T1 TNBC model. Finally, ZG-126 treatment inhibited macrophage infiltration into 4T1 tumors and diminished by 2-fold the proportion of M2-polarized immunosuppressive macrophages. ZG-126 therefore appears to be worthy of further testing as an antitumor and an antimetastatic agent.

## Experimental Section

### Chemical Procedures

All compounds were >95% pure, as determined by HPLC analysis. Unless otherwise stated, reactions were conducted under an argon atmosphere, and glassware was oven-dried prior to use. Tetrahydrofuran and diethyl ether were purified by distillation from sodium under a nitrogen atmosphere. Toluene, dichloromethane, and triethylamine were purified by distillation from calcium hydride under a nitrogen atmosphere. Deuterated chloroform was stored over activated 4 Å molecular sieves. All commercial reagents and solvents were used as purchased without further purification. Thin-layer chromatography (TLC) was carried out on glass-backed Ultrapure silica TLC plates (extra hard layer, 60 Å, thickness: 250 μm, saturated with F-254 indicator) purchased from SiliCycle Inc. Flash column chromatography was carried out on 230–400 mesh silica gel (SiliCycle Inc.) using reagent-grade solvents. Proton and carbon nuclear magnetic resonance spectra were obtained on a Varian 500 or Bruker 500 and 800 MHz spectrometer. Chemical shifts (δ) were internally referenced to the residual proton resonance, including but not limited to CDCl_3_ (δ 7.26 ppm), CD_3_OD (δ 3.31 ppm), and (CD_3_)_2_SO (δ 2.50 ppm). Coupling constants (*J*) are reported in Hertz (Hz). HRMS spectra were obtained by Dr. Nadim Saadeh or Dr. Alexander S. Wahba at McGill University Department of Chemistry. 1,25D (BML-DM200) and SAHA (#10009929) were purchased from Enzo Life Sciences and Cayman Chemical, respectively. They were both used at a final concentration of 100 nM.

#### 1-((6-(3-(4-Hydroxyphenyl)pentan-3-yl)-2-methylpyridin-3-yl)oxy)-3,3-dimethylbutan-2-one (**14**)

Pd/C (97 mg, 10 wt %) was added to the solution of **13**(33) (966 mg, 2.11 mmol) in methanol. The resulting slurry was placed under a 1 atm H_2_ gas atmosphere and stirred vigorously for 14 h at room temperature (RT). The reaction mixture was filtered through a short pad of Celite, and the filtrate was concentrated to give a white solid **14** (740 mg, 2.10 mmol) in 99% yield. The resulting solid was directly used in the next step without further purification. ^1^H NMR (500 MHz, CDCl_3_): δ 7.48 (s, 1H), 7.04 (d, *J* = 8.6 Hz, 1H), 6.84–6.76 (m, 3H), 6.35–6.31 (m, 2H), 4.88 (s, 2H), 2.53 (s, 3H), 2.25 (dd, *J* = 13.6, 7.2 Hz, 2H), 2.05–2.00 (m, 2H), 1.27 (s, 10H), 0.57 (t, *J* = 7.3 Hz, 6H). ^13^C NMR (126 MHz, CDCl_3_): δ 167.2, 157.2, 154.0, 150.5, 146.8, 129.0, 127.9, 127.3, 120.6, 117.6, 77.3, 77.0, 76.7, 69.6, 52.0, 51.9, 33.6, 28.6, 26.0, 19.8, 14.2, 8.3. HRMS: calcd for C_23_H_32_NO_3_ (M + H)^+^, 370.23767; found, 370.23879.

#### 4-(3-(5-(3,3-Dimethyl-2-oxobutoxy)-6-methylpyridin-2-yl)pentan-3-yl)phenyl Trifluoromethanesulfonate (**15**)

Phenol **14** (727 mg, 2.07 mmol) was dissolved in dry DCM (16 mL, 0.13 M) and cooled to 0 °C. Triethylamine (4.13 mmol, 2 equiv) and DMAP (1.03 mmol, 0.5 equiv) were added to the solution, followed by PhNTf_2_ (2.27 mmol, 1.1 equiv). The reaction mixture was stirred for 2 h at 0 °C and then gradually warmed to ambient temperature over 3 h. The reaction was quenched by addition of 1 M HCl until the aqueous layer was pH = 6. The resulting mixture was extracted with DCM (3 × 10 mL). The combined organic layers were washed with brine, dried over solid Na_2_SO_4_, filtered, and concentrated. The residue was purified by flash chromatography on silica gel with 10:90 EtOAc/hexanes to afford a pale-yellow oil **15** (985 mg, 1.96 mmol) in 95% yield. ^1^H NMR (500 MHz, CDCl_3_): δ 7.27–7.24 (m, 2H), 7.17–7.13 (m, 2H), 6.76 (q, *J* = 8.6 Hz, 2H), 4.87 (s, 2H), 2.52 (s, 3H), 2.27 (dq, *J* = 14.7, 7.3 Hz, 2H), 2.21–2.10 (m, 2H), 1.27 (s, 9H), 0.63 (t, *J* = 7.4 Hz, 6H). ^13^C NMR (126 MHz, CDCl_3_): δ 209.3, 157.2, 150.1, 148.9, 147.4, 147.3, 129.6, 120.3, 117.6, 77.3, 77.0, 76.7, 69.2, 51.5, 43.2, 29.7, 28.8, 26.3, 19.8, 8.3. HRMS: calcd for C_24_H_31_F_3_NO_5_S (M + H)^+^, 502.1865; found, 502.18856.

#### Methyl 4-(3-(5-(3,3-Dimethyl-2-oxobutoxy)-6-methylpyridin-2-yl)pentan-3-yl)benzoate (**16**)

Phenyl trifluoromethanesulfonate **15** (1120 mg, 2.23 mmol) was dissolved in dry MeOH (8 mL, 0.28 M). Dry DMSO (4 mL) was added to the solution, followed by Pd(OAc)_2_ (0.22 mmol, 10 mol %) and dppp (0.22 mmol, 10 mol %). The resulting mixture was stirred for 5 min at ambient temperature. Triethylamine was then added dropwise, and the mixture was brought to 80 °C. The reaction was placed under 1 atm CO and stirred vigorously for 24 h. The reaction mixture was quenched with 1 M HCl until the aqueous layer was pH = 6. The resulting mixture was extracted with DCM (3 × 25 mL). The combined organic layers were washed with brine, dried over solid Na_2_SO_4_, filtered, and concentrated. The resulting residue was purified by flash chromatography on silica gel using 15:85 EtOAc/hexanes to afford a yellow oil **16** (799 mg, 1.94 mmol) in 87% yield. ^1^H NMR (500 MHz, CDCl_3_): δ 7.94–7.88 (m, 2H), 7.27–7.24 (m, 2H), 6.76–6.70 (m, 2H), 4.85 (s, 2H), 3.89 (s, 3H), 2.51 (s, 3H), 2.28 (dq, *J* = 14.6, 7.4 Hz, 2H), 2.23–2.12 (m, 2H), 1.25 (s, 9H), 0.62 (t, *J* = 7.4 Hz, 6H). ^13^C NMR (126 MHz, CDCl_3_): δ 209.4, 167.2, 157.6, 153.9, 150.1, 147.3, 129.0, 128.0, 127.3, 120.4, 117.6, 77.3, 77.0, 76.8, 69.3, 52.0, 51.9, 43.2, 28.7, 26.3, 19.8, 8.3. HRMS: calcd for C_25_H_34_NO_4_ (M + H)^+^, 412.24824; found, 412.24907.

#### Methyl 4-(3-(5-(2-Hydroxy-3,3-dimethylbutoxy)-6-methylpyridin-2-yl)pentan-3-yl)benzoate (**17**)

Ketone **16** (43 mg, 0.10 mmol) was dissolved in dry MeOH (2 mL, 0.05 M) at 0 °C. NaBH_4_ (0.15 mmol, 1.5 equiv) was then added portionwise with vigorous stirring. The reaction mixture was allowed to warm to ambient temperature and stirred for 2 h. 1 M HCl was added to acidify the reaction mixture until pH = 6. The resulting mixture was extracted with DCM (3 × 3 mL). The combined organic layers were washed with brine, dried over solid Na_2_SO_4_, filtered, and concentrated. The resulting residue was purified by flash chromatography on silica gel using 15:85 EtOAc/hexanes to give a pale-yellow oil **17** (42 mg, 0.10 mmol) in 82% yield. ^1^H NMR (500 MHz, CDCl_3_): δ 7.93–7.88 (m, 2H), 7.25 (d, *J* = 8.4 Hz, 2H), 6.94 (d, *J* = 8.5 Hz, 1H), 6.79 (d, *J* = 8.5 Hz, 1H), 4.06 (dd, *J* = 9.2, 2.6 Hz, 1H), 3.88 (s, 3H), 3.84 (t, *J* = 8.9 Hz, 1H), 3.71 (dt, *J* = 8.8, 2.5 Hz, 1H), 2.44 (s, 3H), 2.35 (d, *J* = 3.1 Hz, 1H), 2.34–2.12 (m, 4H), 1.01 (s, 9H), 0.61 (t, *J* = 7.3 Hz, 6H). ^13^C NMR (126 MHz, CDCl_3_): δ 167.2, 157.2, 154.0, 150.5, 146.8, 129.0, 127.9, 127.3, 120.6, 117.6, 77.3, 77.0, 76.7, 69.6, 52.0, 51.9, 33.6, 28.6, 26.0, 19.8, 14.2, 8.3. HRMS: calcd for C_25_H_36_NO_4_ (M + H)^+^, 414.26389; found, 414.26437.

#### 4-(3-(5-(2-Hydroxy-3,3-dimethylbutoxy)-6-methylpyridin-2-yl)pentan-3-yl)benzoic Acid (**18**)

Methyl benzoate **17** (571 mg, 1.38 mmol) was dissolved in a mixture of THF/MeOH/H_2_O (1:1:1, v/v/v, 12 mL). LiOH was then added to the solution in one portion. The reaction mixture was stirred vigorously at ambient temperature for 14 h. 1 M HCl was added to the reaction mixture until pH = 1. The resulting mixture was extracted with EtOAc (4 × 10 mL). The combined organic layers were washed with brine, dried over solid Na_2_SO_4_, filtered, and concentrated. The resulting residue was purified by flash chromatography on silica gel using 40:60:1 EtOAc/hexanes/AcOH to afford a white solid **18** (353 mg, 0.88 mmol) in 64% yield. ^1^H NMR (500 MHz, CD_3_OD): δ 7.93–7.86 (m, 2H), 7.27–7.23 (m, 2H), 7.22 (d, *J* = 8.6 Hz, 1H), 7.08 (d, *J* = 8.6 Hz, 1H), 4.17 (dd, *J* = 10.0, 2.8 Hz, 1H), 3.90 (dd, *J* = 10.0, 7.9 Hz, 1H), 3.64 (dd, *J* = 7.9, 2.7 Hz, 1H), 2.41 (s, 3H), 2.36–2.26 (m, 2H), 2.21 (dq, *J* = 14.4, 7.3 Hz, 2H), 1.01 (s, 9H), 0.63 (t, *J* = 7.3 Hz, 6H). ^13^C NMR (126 MHz, CD_3_OD): δ 173.9, 156.2, 153.7, 151.3, 147.4, 128.7, 127.7, 120.5, 117.5, 77.1, 69.8, 51.6, 48.1, 47.9, 47.7, 47.6, 47.4, 47.2, 47.1, 33.7, 28.0, 25.1, 17.7, 7.3. HRMS: calcd for C_24_H_34_NO_4_ (M + H)^+^, 400.24824; found, 400.24826.

#### Methyl 4-(4-(3-(5-(2-Hydroxy-3,3-dimethylbutoxy)-6-methylpyridin-2-yl)pentan-3-yl)benzamido)butanoate (**19a**)

To a flame-dried round-bottom flask were added benzoic acid **18** (41 mg, 0.10 mmol) and dry DCM (10 mL, 0.01 M). Methyl 4-aminobutyrate (0.20 mmol, 2 equiv) was then added to the solution in one portion with vigorous stirring, followed by EDC·HCl (0.20 mmol, 2 equiv), DMAP (0.01 mmol, 10 mol %), and triethylamine (0.26 mmol, 2.5 equiv). The reaction mixture was stirred vigorously at ambient temperature for 16 h. 1 M HCl was added to quench the reaction until the aqueous layer was pH = 6. The resulting mixture was extracted with EtOAc (3 × 10 mL). The combined organic layers were washed with brine, dried over solid Na_2_SO_4_, filtered, and concentrated. The resulting residue was purified by flash chromatography on silica gel using 50:50 EtOAc/hexanes to give a pale-yellow oil **19a** (49 mg, 0.10 mmol) in 95% yield. ^1^H NMR (800 MHz, CD_3_OD): δ 7.73–7.69 (m, 2H), 7.27–7.23 (m, 2H), 7.22 (d, *J* = 8.6 Hz, 1H), 7.08 (d, *J* = 8.5 Hz, 1H), 4.18 (dd, *J* = 10.0, 2.8 Hz, 1H), 3.91 (dd, *J* = 10.0, 7.9 Hz, 1H), 3.66 (s, 4H), 3.42 (t, *J* = 6.9 Hz, 2H), 2.45–2.41 (m, 5H), 2.32 (dqd, *J* = 14.8, 7.3, 1.9 Hz, 2H), 2.21 (dt, *J* = 13.7, 7.3 Hz, 2H), 1.92 (p, *J* = 7.2 Hz, 2H), 1.03 (s, 9H), 0.63 (t, *J* = 7.4 Hz, 6H). ^13^C NMR (201 MHz, CD_3_OD): δ 174.1, 168.9, 156.3, 152.2, 151.3, 147.3, 131.3, 127.8, 126.2, 120.5, 117.5, 77.1, 69.8, 51.5, 50.7, 48.1, 48.0, 47.9, 47.8, 47.7, 47.6, 47.5, 47.4, 47.3, 38.8, 33.7, 30.8, 28.0, 25.1, 24.4, 17.8, 7.3. HRMS: calcd for C_29_H_43_N_2_O_5_ (M + H)^+^, 49.31665; found, 49.31537.

#### 4-(3-(5-(2-Hydroxy-3,3-dimethylbutoxy)-6-methylpyridin-2-yl)pentan-3-yl)-*N*-(4-(hydroxyamino)-4-oxobutyl)benzamide (**20a**)

Methyl ester **19a** (43 mg, 0.09 mmol) was dissolved in a mixture of THF/MeOH (1:1, v/v, 1.6 mL, 0.05 M) at 0 °C. Precooled 50 wt % NH_2_OH solution (43.31 mmol, 500 equiv) was added dropwise with vigorous stirring over 5 min, followed by 3 M KOH solution (202 μL), sufficient to keep the pH of the reaction mixture between 9 and 10. The reaction was then allowed to warm to ambient temperature and stirred vigorously for 16 h. The organic solvent was evaporated, and the resulting aqueous residue was acidified with 1 M HCl until pH = 7 to give a white slurry. The resulting slurry was extracted with EtOAc (3 × 3 mL). The combined organic layers were washed with brine, dried over solid Na_2_SO_4_, filtered, and concentrated. The residue was purified by flash chromatography on C18 silica gel using 65–75% MeOH/H_2_O. Collected fractions were concentrated and freeze-dried on a lyophilizer to afford a white powder **20a** (23 mg, 0.05 mmol) in 53% yield. ^1^H NMR (800 MHz, CD_3_OD): δ 7.62–7.58 (m, 2H), 7.15–7.11 (m, 2H), 7.10 (d, *J* = 8.6 Hz, 1H), 6.96 (d, *J* = 8.6 Hz, 1H), 4.06 (dd, *J* = 10.0, 2.8 Hz, 1H), 3.79 (dd, *J* = 10.0, 7.9 Hz, 1H), 3.53 (dd, *J* = 7.9, 2.8 Hz, 1H), 3.29 (t, *J* = 6.9 Hz, 2H), 2.29 (s, 3H), 2.20 (dqd, *J* = 14.8, 7.3, 1.8 Hz, 2H), 2.12–2.05 (m, 4H), 1.80 (p, *J* = 7.1 Hz, 2H), 0.91 (s, 9H), 0.52 (t, *J* = 7.3 Hz, 6H). ^13^C NMR (201 MHz, CD_3_OD): δ 171.0, 168.9, 156.3, 152.2, 151.3, 147.3, 131.3, 127.8, 126.2, 120.5, 117.5, 77.1, 69.8, 51.5, 47.9, 47.8, 47.7, 47.6, 47.5, 47.4, 47.3, 38.9, 33.7, 29.9, 28.0, 25.3, 25.1, 17.8, 7.3. HRMS: calcd for C_28_H_42_N_3_O_5_ (M + H)^+^, 500.31190; found, 500.31204.

#### Methyl 5-(4-(3-(5-(2-Hydroxy-3,3-dimethylbutoxy)-6-methylpyridin-2-yl)pentan-3-yl)benzamido)pentanoate (**19b**)

To a flame-dried round-bottom flask were added benzoic acid **18** (42 mg, 0.10 mmol) and dry DCM (10 mL, 0.01 M). Methyl 5-aminopentanoate (0.21 mmol, 2 equiv) was then added to the solution in one portion with vigorous stirring, followed by EDC·HCl (0.21 mmol, 2 equiv), DMAP (0.01 mmol, 10 mol %), and triethylamine (0.26 mmol, 2.5 equiv). The reaction mixture was stirred vigorously at ambient temperature for 16 h. 1 M HCl was added to quench the reaction until the aqueous layer was pH = 6. The resulting mixture was extracted with EtOAc (3 × 10 mL). The combined organic layers were washed with brine, dried over solid Na_2_SO_4_, filtered, and concentrated. The resulting residue was purified by flash chromatography on silica gel using 50:50 EtOAc/hexanes to give a pale-yellow oil **19b** (47 mg, 0.09 mmol) in 89% yield. ^1^H NMR (500 MHz, CD_3_OD): δ 8.38 (t, *J* = 5.8 Hz, 1H), 7.73–7.67 (m, 2H), 7.27–7.22 (m, 2H), 7.22 (s, 1H), 7.07 (d, *J* = 8.6 Hz, 1H), 4.17 (dd, *J* = 10.0, 2.8 Hz, 1H), 3.90 (dd, *J* = 10.0, 7.9 Hz, 1H), 3.67–3.61 (m, 4H), 3.42–3.35 (m, 2H), 2.42–2.36 (m, 5H), 2.31 (ddd, *J* = 13.6, 7.3, 1.1 Hz, 2H), 2.20 (dq, *J* = 14.4, 7.3 Hz, 2H), 1.74–1.59 (m, 4H), 1.02 (s, 9H), 0.62 (t, *J* = 7.3 Hz, 6H). ^13^C NMR (126 MHz, CD_3_OD): δ 174.3, 168.8, 156.3, 152.1, 151.3, 147.3, 131.4, 127.8, 126.2, 120.5, 117.5, 77.1, 69.8, 51.5, 50.6, 39.0, 33.7, 32.9, 28.5, 28.0, 25.1, 21.9, 17.8, 7.3. HRMS: calcd for C_30_H_45_N_2_O_5_ (M + H)^+^, 513.33230; found, 513.33226.

#### 4-(3-(5-(2-Hydroxy-3,3-dimethylbutoxy)-6-methylpyridin-2-yl)pentan-3-yl)-*N*-(5-(hydroxyamino)-5-oxopentyl)benzamide (**20b**)

Methyl ester **19b** (42 mg, 0.08 mmol) was dissolved in a mixture of THF/MeOH (1:1, v/v, 1.6 mL, 0.05 M) at 0 °C. A precooled 50 wt % NH_2_OH solution (40.96 mmol, 500 equiv) was added dropwise with vigorous stirring over 5 min, followed by 3 M KOH solution (191 μL), sufficient to keep the pH of the reaction mixture between 9 and 10. The reaction was then allowed to warm to ambient temperature and stirred vigorously for 16 h. The organic solvent was evaporated, and the resulting aqueous residue was acidified with 1 M HCl until pH = 7 to give a white slurry. The resulting slurry was extracted with EtOAc (3 × 3 mL). The combined organic layers were washed with brine, dried over solid Na_2_SO_4_, filtered, and concentrated. The residue was purified by flash chromatography on C18 silica gel using 65–75% MeOH/H_2_O. Collected fractions were concentrated and freeze-dried on a lyophilizer to afford a white powder **20b** (15 mg, 0.03 mmol) in 15% yield. ^1^H NMR (800 MHz, CD_3_OD): δ 7.61–7.57 (m, 2H), 7.15–7.11 (m, 2H), 7.10 (d, *J* = 8.6 Hz, 1H), 6.96 (d, *J* = 8.5 Hz, 1H), 4.06 (dd, *J* = 10.0, 2.8 Hz, 1H), 3.79 (dd, *J* = 10.0, 7.9 Hz, 1H), 3.53 (dd, *J* = 7.9, 2.8 Hz, 1H), 3.27 (*t*, *J* = 6.9 Hz, 2H), 2.29 (s, 3H), 2.20 (dqd, *J* = 14.9, 7.4, 2.0 Hz, 2H), 2.12–2.06 (m, 2H), 2.04 (*t*, *J* = 7.3 Hz, 2H), 1.58 (dp, *J* = 11.0, 6.9 Hz, 2H), 1.52 (tdd, *J* = 10.2, 6.9, 5.4 Hz, 2H), 0.91 (s, 9H), 0.51 (*t*, *J* = 7.3 Hz, 6H). ^13^C NMR (201 MHz, CD_3_OD): δ 171.3, 168.8, 156.4, 152.1, 151.3, 147.3, 131.4, 127.8, 126.2, 120.5, 117.5, 77.1, 69.8, 51.5, 39.0, 33.7, 31.9, 28.5, 28.0, 25.1, 22.7, 17.8, 7.3. HRMS calcd for C_29_H_44_N_3_O_5_ (M + H)^+^: 514.32755; found, 514.32727.

#### Methyl 6-(4-(3-(5-(2-Hydroxy-3,3-dimethylbutoxy)-6-methylpyridin-2-yl)pentan-3-yl)benzamido)hexanoate (**19c**)

To a flame-dried round-bottom flask were added benzoic acid **18** (36 mg, 0.09 mmol) and dry DCM (9 mL, 0.01 M). Methyl 6-aminohexanoate (0.18 mmol, 2 equiv) was then added to the vigorously stirred solution in one portion, followed by EDC·HCl (0.18 mmol, 2 equiv), DMAP (0.01 mmol, 10 mol %), and triethylamine (0.23 mmol, 2.5 equiv). The reaction mixture was stirred vigorously at ambient temperature for 16 h. 1 M HCl was added to quench the reaction until the aqueous layer was pH = 6. The resulting mixture was extracted with EtOAc (3 × 10 mL). The combined organic layers were washed with brine, dried over solid Na_2_SO_4_, filtered, and concentrated. The resulting residue was purified by flash chromatography on silica gel using 50:50 EtOAc/hexanes to give a pale-yellow oil **19c** (47 mg, 0.09 mmol) in 98% yield. ^1^H NMR (800 MHz, CD_3_OD): δ 7.72–7.68 (m, 2H), 7.26–7.23 (m, 2H), 7.21 (d, *J* = 8.6 Hz, 1H), 7.09–7.06 (m, 1H), 4.18 (dd, *J* = 10.0, 2.8 Hz, 1H), 3.91 (dd, *J* = 9.9, 7.9 Hz, 1H), 3.65 (s, 4H), 3.37 (t, *J* = 7.1 Hz, 2H), 2.42 (s, 3H), 2.36 (d, *J* = 7.4 Hz, 1H), 2.32 (dqd, *J* = 14.8, 7.4, 6.4, 2.2 Hz, 2H), 2.21 (dq, *J* = 14.4, 7.3 Hz, 2H), 1.66 (ddt, *J* = 29.7, 14.9, 7.3 Hz, 4H), 1.45–1.39 (m, 2H), 1.03 (s, 9H), 0.63 (t, *J* = 7.4 Hz, 6H). ^13^C NMR (201 MHz, CD_3_OD): δ 174.5, 174.3, 168.8, 156.4, 152.1, 151.3, 147.3, 131.5, 127.8, 126.2, 120.5, 117.5, 77.1, 69.8, 51.5, 50.6, 48.1, 47.9, 47.8, 47.7, 47.6, 47.5, 47.4, 47.3, 39.3, 33.7, 33.3, 28.7, 28.0, 26.1, 25.1, 24.3, 19.7, 17.8, 7.3. HRMS: calcd for C_31_H_47_N_2_O_5_ (M + H)^+^, 527.34795; found, 527.34753.

#### 4-(3-(5-(2-Hydroxy-3,3-dimethylbutoxy)-6-methylpyridin-2-yl)pentan-3-yl)-*N*-(6-(hydroxyamino)-6-oxohexyl)benzamide (**20c**)

Methyl ester **19c** (42 mg, 0.08 mmol) was dissolved in a mixture of THF/MeOH (1:1, v/v, 1.6 mL, 0.05 M) at 0 °C. A precooled 50 wt % NH_2_OH solution (39.39 mmol, 500 equiv) was added dropwise with vigorous stirring over 5 min, followed by 3 M KOH solution (183 μL), sufficient to keep the pH of the reaction mixture between 9 and 10. The reaction was then allowed to warm to ambient temperature and stirred vigorously for 16 h. The organic solvent was evaporated, and the resulting aqueous residue was acidified with 1 M HCl until pH = 7 to give a white slurry. The resulting slurry was extracted with EtOAc (3 × 3 mL). The combined organic layers were washed with brine, dried over solid Na_2_SO_4_, filtered, and concentrated. The residue was purified by flash chromatography on C18 silica gel using 65–75% MeOH/H_2_O. Collected fractions were concentrated and freeze-dried on a lyophilizer to afford a white powder **20c** (11 mg, 0.02 mmol) in 26% yield. ^1^H NMR (800 MHz, CD_3_OD): δ 7.58 (d, *J* = 8.6 Hz, 2H), 7.14–7.11 (m, 2H), 7.10 (d, *J* = 8.6 Hz, 1H), 6.96 (d, *J* = 8.6 Hz, 1H), 4.06 (dd, *J* = 10.0, 2.8 Hz, 1H), 3.79 (dd, *J* = 10.0, 7.9 Hz, 1H), 3.53 (dd, *J* = 7.9, 2.8 Hz, 1H), 2.30 (s, 3H), 2.20 (dqd, *J* = 14.9, 7.5, 1.8 Hz, 2H), 2.09 (dq, *J* = 14.4, 7.3 Hz, 2H), 2.00 (t, *J* = 7.4 Hz, 2H), 1.58–1.51 (m, 4H), 1.30 (tt, *J* = 10.0, 6.6 Hz, 2H), 0.91 (s, 10H), 0.52 (t, *J* = 7.4 Hz, 7H). ^13^C NMR (201 MHz, CD_3_OD): δ 171.5, 168.8, 156.4, 152.1, 151.3, 147.3, 131.5, 127.8, 126.2, 120.5, 117.5, 77.1, 69.8, 51.5, 39.3, 33.7, 32.3, 28.8, 28.0, 26.1, 25.1, 25.0, 17.8, 7.3. HRMS: calcd for C_30_H_46_N_3_O_5_ (M + H)^+^, 528.34320; found, 528.34333.

### Biological Evaluation

#### Cell Culture

Mouse 4T1 and 4TO7 TNBC cells and B16-F10 mouse melanoma cells were cultured in Dulbecco’s modified Eagle medium (DMEM; 319-005-CL; WISENT Inc.) supplemented with 10% heat-inactivated fetal bovine serum (FBS; 098150; WISENT Inc.). Human TNBC cells, MDA-MB-231, were cultured in Leibovitz’s L-15 medium (323-050-CL; WISENT Inc.) with 10% heat-inactivated FBS in the absence of CO_2_. Cells were then treated with DMSO (vehicle), 1,25D, SAHA, DK-406, AM-193, or ZG hybrids (102, 126, or 132) for 6 or 24 h.

#### RealTime-Glo Cell Viability Assay

To determine the cytotoxicity of hybrid molecules, the assay was performed at 24 h after treatment of 4T1, MDA-MB-231, or B16-F10 cells according to the manufacturer’s instructions (Promega-#G9712). Luminescence was measured using a 1420 Luminescence Counter Victor Light (PerkinElmer), normalized to control, and plotted. All samples were run in triplicate.

#### RNA Extraction, Reverse Transcription, and qPCR

RNA extraction was performed with the FavorPrep tissue total RNA mini kit (FAVORGEN Biotech Corporation FATRK 001) as per manufacturer’s instructions. cDNA was obtained from 1 μg of RNA using 5× All-in-One RT MasterMix (Applied Biological Materials (abm) Inc. G485) and diluted 5 times. qPCR was performed with BrightGreen 2× q-PCR MasterMix (abm MasterMix-LR-XL) on a Roche LightCycler 96 system. Expression of targeted genes was normalized to 18 s. All primers are listed in Table S2.

#### Biochemical Assays for HDAC Inhibition

These assays were performed by Reaction Biology (Malvern, PA, USA) using standard in-house protocols. Briefly, ZG-126 was tested in 10-dose IC50 mode, in singlet, with 3-fold serial dilution starting at 100 μM. The HDACi reference compound Trichostatin A (TSA) was tested in a 10-dose IC50 mode, with 3-fold serial dilution starting at 10 or 1 μM, depending on the HDAC tested. The HDAC reference compound TMP269 was tested in a 10-dose IC50 mode, with 3-fold serial dilution starting at 10 μM. The HDAC reference compound quisinostat was tested in a 10-dose IC50 mode with 3-fold serial dilution starting at 1 μM. For HDACs 1, 2, 3, and 6, a fluorogenic peptide from p53 residues 379–382 (RHKK(Ac)AMC) was used as a substrate. For HDACs 4, 5, 7, 9, and 11, a fluorogenic HDAC Class IIa substrate trifluoroacetyl lysine was used. For HDAC8, a fluorogenic peptide from p53 residues 379–382 [RHK(Ac)K(Ac)AMC] was used as a substrate, whereas Ac-spermidine-AMC was the substrate for HDAC10 assays.

#### Western Blotting and Protein Analyses

After 6 h treatments, 4T1, 4T07, or B16-F10 cells were solubilized in lysis buffer (20 mM Tris, pH 8, 150 mM NaCl, 1% Triton X-100, 3.5 mM SDS, and 13 mM deoxycholic acid), and proteins were separated on a 4–15% Tris/glycine/SDS gel (Bio-Rad). Then, the proteins were transferred to a nitrocellulose membrane, followed by blocking in 5% skim milk in Tris-buffered saline (TBS, 1×) solution. After blocking, the membranes were incubated overnight at 4 °C with the primary antibodies against acetylated tubulin (Sigma-Aldrich—#T7451), tubulin (Sigma-Aldrich—#T9026), acetyl-histone H3(Lys9) (Merck Millipore—#07-352), acetyl-histone H3 (Lys27) (Abcam—#ab4729), total histone H3 (Cell Signaling Technology (CST)—#3638), and GAPDH (Abcam—#ab8245). Then, the membranes were incubated with antimouse (CST—#7076) or antirabbit (CST—#7074) IgG HRP-linked secondary antibodies at recommended concentrations. Finally, signals were detected using Clarity ECL substrates (Bio-Rad) and a ChemiDoc Imaging System. Changes in protein levels were quantified relative to control using Image Lab software (version 6.0.1) after normalization to GAPDH. Western blot experiments were carried out three times.

#### In Vivo Efficacy and MTD Studies

All animal experiments were carried out according to McGill University Animal Care guidelines by Dr. Krikor Bijian at the Lady Davis Institute. Briefly, 4T1 cells (1 × 10^6^ cells per mouse) or B16-F10 cells (2.5 × 10^5^ per mouse) were implanted in the mammary fat pad of female Balb/c mice or subcutaneously in female C57BL/6 mice, respectively. As soon as the tumors became palpable (days 7–14), treatments began by an intraperitoneal (IP) injection of DMSO (vehicle), 1,25D, and/or SAHA or the hybrid molecules every second day, as indicated in the studies (250 μL total volume). Throughout the course of treatment (18 days), the primary tumor size was measured 5 times using a caliper, and the tumor volume was calculated as (length × width^2^)/2. Finally, mice were sacrificed, and lungs of 4T1-bearing mice were removed to evaluate the efficiency of treatments on lung metastases. As such, lungs were fixed in 10% Bouin’s fixative, and surface lung metastases were counted using a stereomicroscope (Optimax; Leica). For MTD studies, Balb/c mice were given increasing daily doses of indicated compounds IP (0.5, 1.0, 1.5, 2.5, 5, and 10 mg/kg). Once mice demonstrated any sign of discomfort (poor oral intake, lethargy, weight loss, inability to close mouth, drooling, and pawing at mouth), the study was terminated.

#### Immunofluorescence Microscopy

Primary tumor tissues were fixed using 4% paraformaldehyde and subsequently dehydrated in sucrose solutions ranging from 10 to 30% for approximately 24 h each. The tissues were then embedded in base molds (Fisherbrand—#22363553) containing an optimal cutting temperature (OCT) compound (Fisher Healthcare—#4585). The tissue blocks were placed immediately on dry ice until the OCT compound solidified, and then they were stored at −80 °C. Prior to IF staining, sections of 5–10 μm were obtained using a Leica CM3050 S cryostat and SuperFrost Plus slides (Fisherbrand—#1255015). The tumor sections were then incubated with blocking buffer [phosphate-buffered saline containing 2% bovine serum albumin, 0.3% Triton X-100, 1% FBS, and 10% goat serum] for 1 h at RT. To detect macrophages in the TME, the sections were incubated with a pan-macrophage rat monoclonal antibody F4/80 (Abcam—#6640) diluted in blocking buffer overnight at 4 °C. Sixteen hours later, tissues were washed 3 times with 100 mM Tris–HCl and then incubated with a secondary goat antirat antibody (Invitrogen—#A11081) for 1 h at RT. The washing step was repeated, followed by incubation with an M2 macrophage marker, Alexa-647 Arg1 (CST—#43279) for 90 min at RT. Subsequently, 4′,6-diamidino-2-phenylindole (DAPI; Invitrogen—#D3571) was used to counterstain the nuclei for 5 min at RT. Finally, the slides were mounted using Fluoromount-G medium (Invitrogen—#00495802) and coverslips (Fisher Scientific—#12545E) and imaged with a Zeiss LSM 710 confocal microscope with ×20 objective. Quantification was carried out by QuPath software (version 0.3.2). F4/80+ macrophages and F4/80+ Arg1+ M2 macrophages were classified based on their fluorescence signal.

#### Statistical Analyses

Statistical calculations were performed using GraphPad Prism (version 8). A parametric unpaired *t* test was used for comparison of two groups in all in vitro experiments, while a two-way ANOVA was used to examine the differences in primary tumor volume in the in vivo experiment. Statistical significance is indicated as follows: *p < 0.05, **p < 0.01, ***p < 0.001, and ****p < 0.0001.

## Supporting Information Available

Additional figures illustrating NMR spectra, HRMS, and HPLC reports from synthesized compounds; dose–response profile for the inhibition of different HDACs by ZG-126; in vitro effect of ZG-126 in B16-F10 cells; representative IF images showing the macrophage population in 4T1 tumors under different conditions; details of primer sequences used for RT-qPCR; and compound IC50 values extracted from the in vitro cell viability assay (PDF)
Molecular formula strings (CSV)

## Abbreviations

125D: 1α,25-dihydroxyvitamin D3
ANOVA: analysis of variance
Arg1: arginase 1
Ccl2: chemokine (C–C motif) ligand 2
Ccl5: chemokine (C–C motif) ligand 5
CCL18: chemokine (C–C motif) ligand 18
Ccl20: chemokine (C–C motif) ligand 20
CCL22: chemokine (C–C motif) ligand 22
CD80/CD86: cluster of differentiation 80/86
C NMR: carbon nuclear magnetic resonance
COX2: cyclooxygenase 2
CTLA-4: cytotoxic T-lymphocyte-associated protein 4
Cxcl10: C-X-C motif chemokine ligand 10
CYP24A1: cytochrome P450 family 24 subfamily A member 1
CYP27B1: cytochrome P450 family 27 subfamily B member 1
DAPI: 4′,6-diamidino-2-phenylindole
DMEM: Dulbecco’s modified Eagle medium
ECL: enhanced chemiluminescence
FBS: fetal bovine serum
GAPDH: glyceraldehyde 3-phosphate dehydrogenase
HDACi: histone deacetylase inhibitor
HER2: human epidermal growth factor receptor 2
^1^H NMR: proton nuclear magnetic resonance
HNSCC: head and neck squamous carcinoma
HRP: horseradish peroxidase
HSP90: heat shock protein 90
IF: immunofluorescence
Il1α: interleukin-1α
IL-4: interleukin-4
IL-10: interleukin-10
LSM: laser scanning microscopy
MMP: matrix metalloproteinase
OCT: optimal cutting temperature
PD-1: programmed cell death protein 1
PD-L1: programmed death ligand 1
qPCR: quantitative polymerase chain reaction
TBS: Tris-buffered saline
TGFβ: transforming growth factor beta
TME: tumor microenvironment
*TNBC*: triple-negative breast cancer
TSA: Trichostatin A
VDR: vitamin D receptor
