# Supplementary material for: Bifunctionality and Antitumor Efficacy of ZG-126, a Vitamin D Receptor Agonist/Histone Deacetylase Inhibitor Hybrid Molecule
Source: J Med Chem. 2024 Jun 21;67(13):11182–96. doi: 10.1021/acs.jmedchem.4c00706 (PMC11249012; doi:10.1021/acs.jmedchem.4c00706)
Supplement: Supplementary file 1 — jm4c00706_si_001.pdf [file jm4c00706_si_001.pdf]

Supporting Information

Bifunctionality and anti-tumor efficacy of ZG-126, a vitamin D receptor agonist/ histone deacetylase inhibitor hybrid molecule.

Fatemeh Sarmadi[a], Zhizhong Gao[c], Jie Su[d], Camille Barbier[a], Patricio Artusa[a], Krikor Bijian[d]*, James L. Gleason[c]*, John H. White[a,b]*

[a] *Department of Physiology, McGill University, 3655 Promenade Sir William Osler, Montreal, QC, H3G 1Y6, Canada*
[b] *Department of Medicine, McGill University, 3655 Promenade Sir William Osler, Montreal, QC, H3G 1Y6, Canada*
[c] *Department of Chemistry, McGill University, 801 Sherbrooke W., Montreal, QC, H3A 0B8, Canada*
[d] *Segal Cancer Center and Lady Davis Institute for Medical Research, 3755 Cote Ste-Catherine, Montreal, QC, H3T 1E2, Canada*

* Address correspondence to J.H.W. for molecular/cell biology: john.white@mcgill.ca
Address correspondence to J.L.G. for chemistry: jim.gleason@mcgill.ca
Address correspondence to K.B. for animal studies: krikor.bijian@mail.mcgill.ca

Contents

NMR spectra and HRMS for all new compounds synthesized S2
HPLC reports of 20a (ZG-132), 20b (ZG-126), and 20c (ZG-102) S35
Dose response profiles for inhibition of different HDACs by ZG-126. .... S41
Bifunctionality of ZG-126 in mouse melanoma B16-F10 cells *in vitro*. S42
Effect of ZG-126 on recruitment and polarization of macrophages in 4T1 tumors S43
Table S1. Hybrid molecules IC50s extracted from the *in vitro* cell viability assay. S44
Table S2. Primer sequences for RT-qPCR S45

NMR spectra and HRMS for all new compounds synthesized

14 ^{1}H NMR (500 MHz, $CDCl_3$)

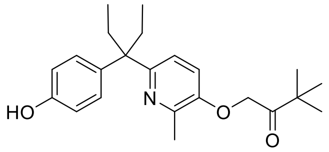

14

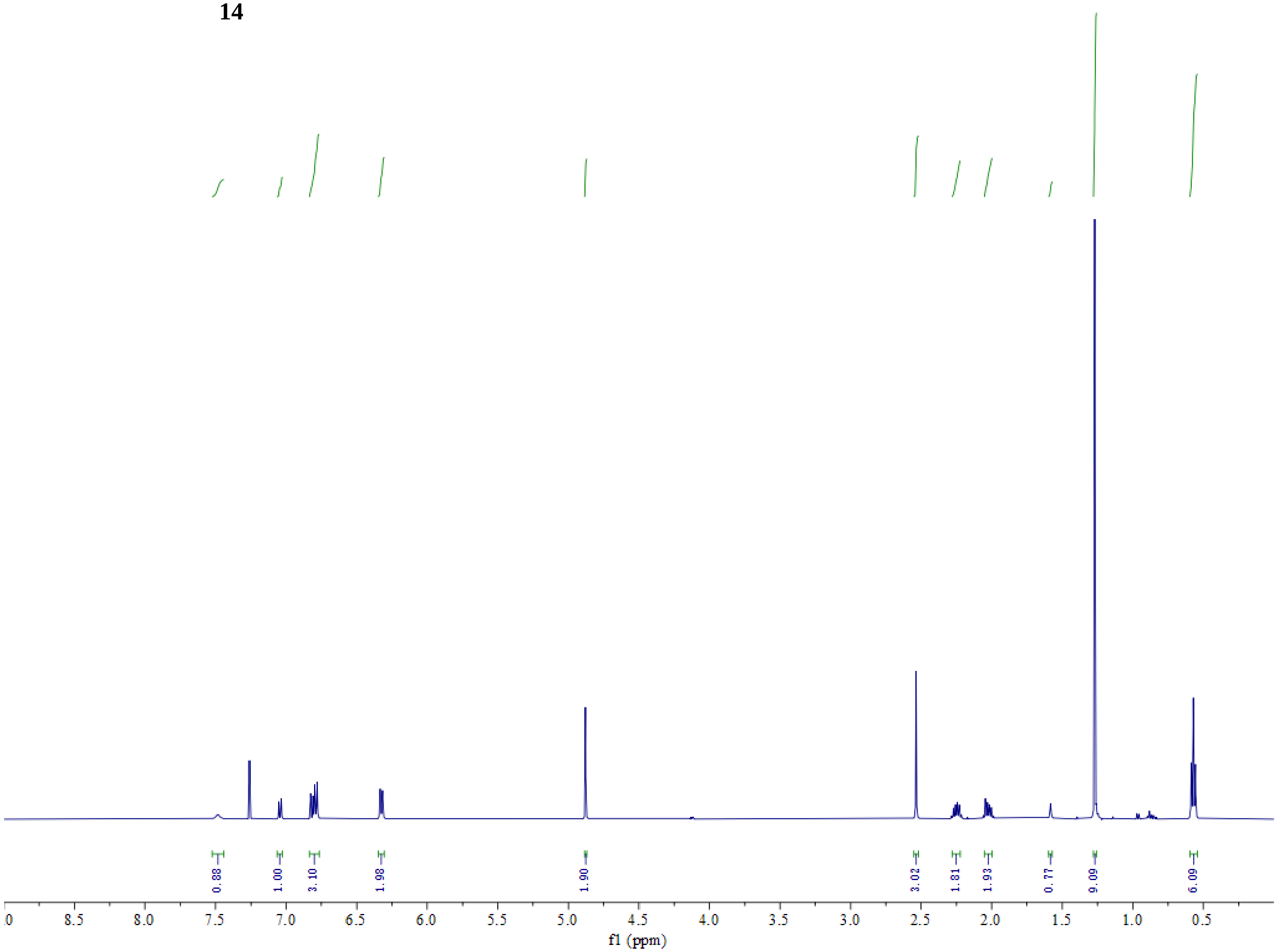

14 ^{13}C NMR (126 MHz, CDCl$_3$)

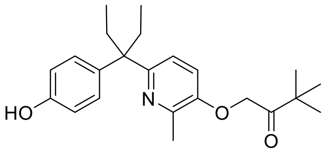

14

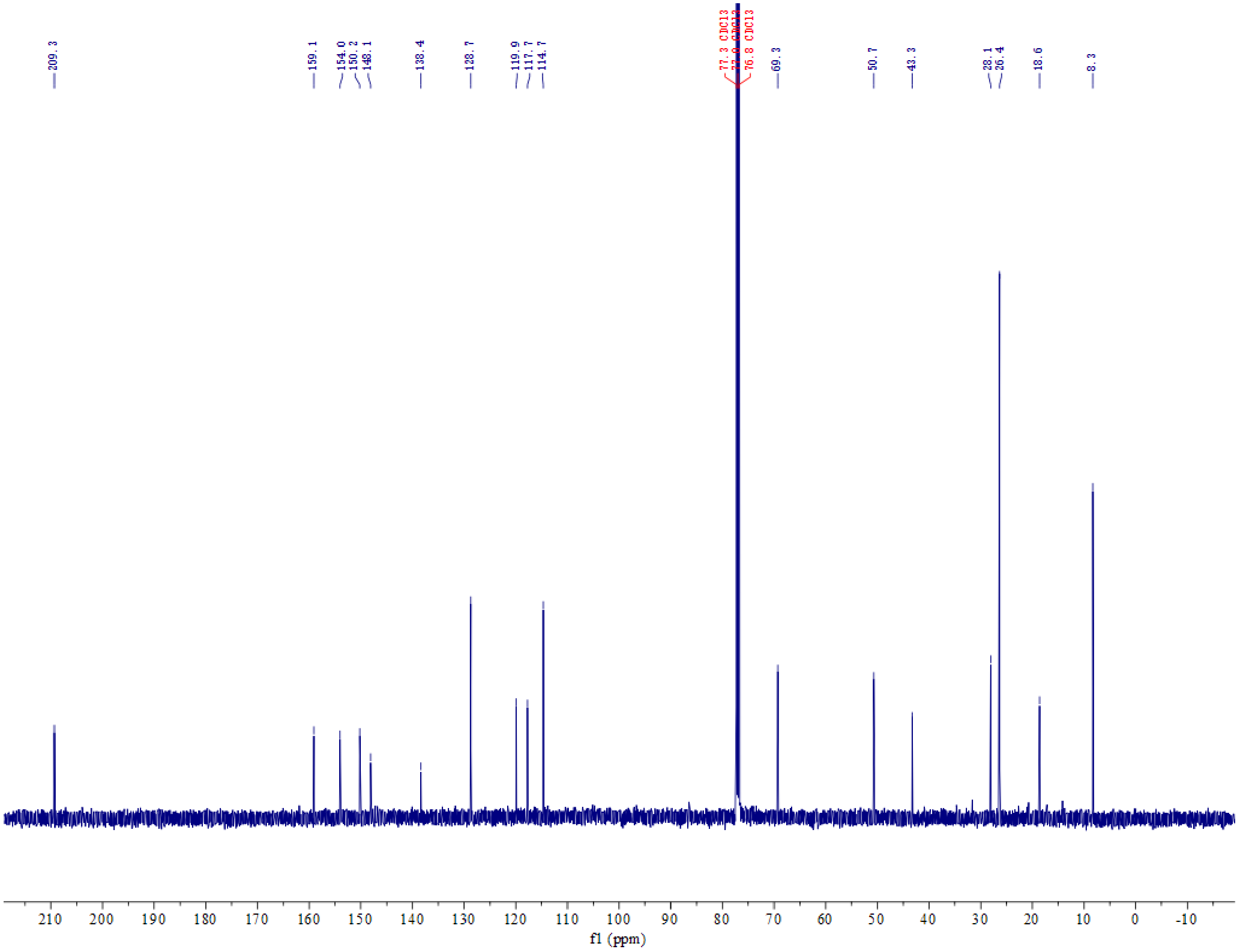

14 HRMS

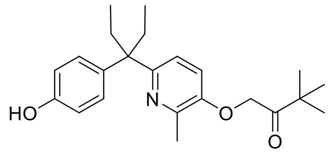

14

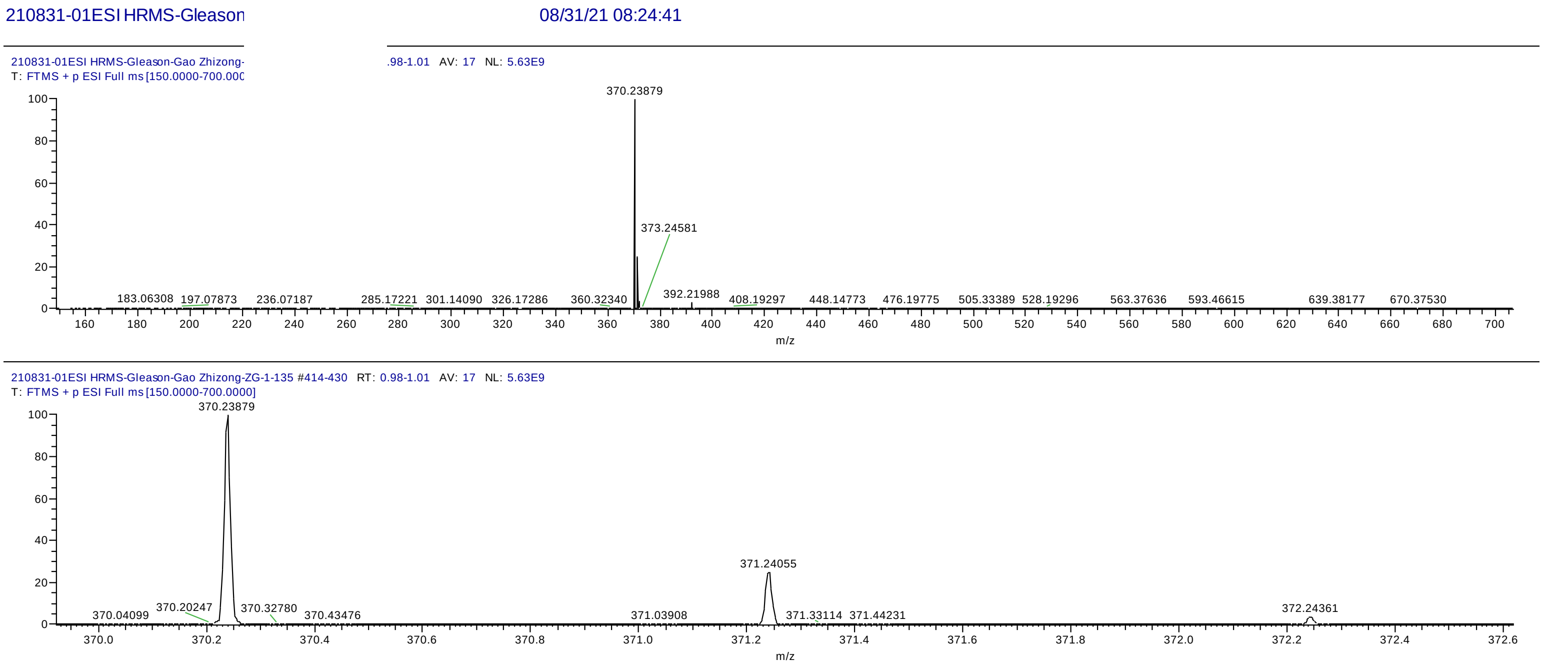

210831-01ESI HRMS-Gleason-Gao Zhizong-ZG-1-135#414-430 RT: 0.98-1.01 AV: 17
T: FTMS + p ESI Full ms [150.0000-700.0000]
m/z= 370.22046-370.24934

m/z	Intensity	Relative	Resolution	Charge	Theo. Mass	Delta (ppm)	RDB equiv.	Composition
370.23879	5713825792.0	100.00	28149.39	1.00	370.23767	3.03	8.5	$C_{23}H_{32}O_3N$

15 ^{1}H NMR (500 MHz, $CDCl_3$)

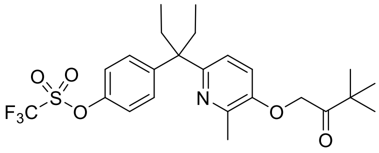

15

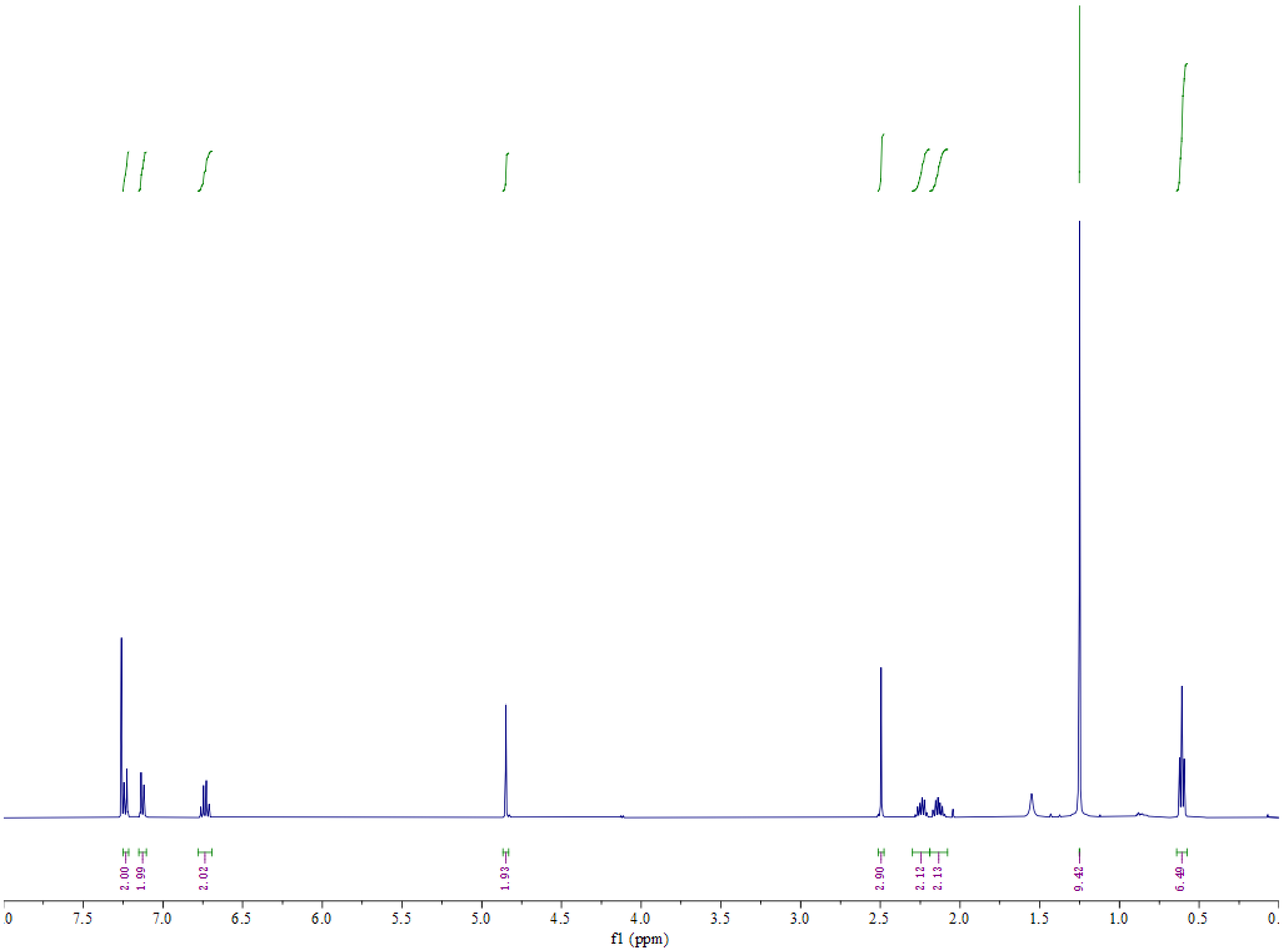

15 ^{13}C NMR (126 MHz, $CDCl_3$)

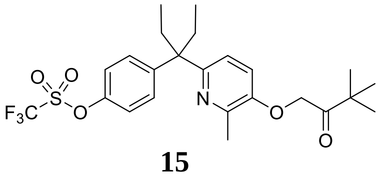

15

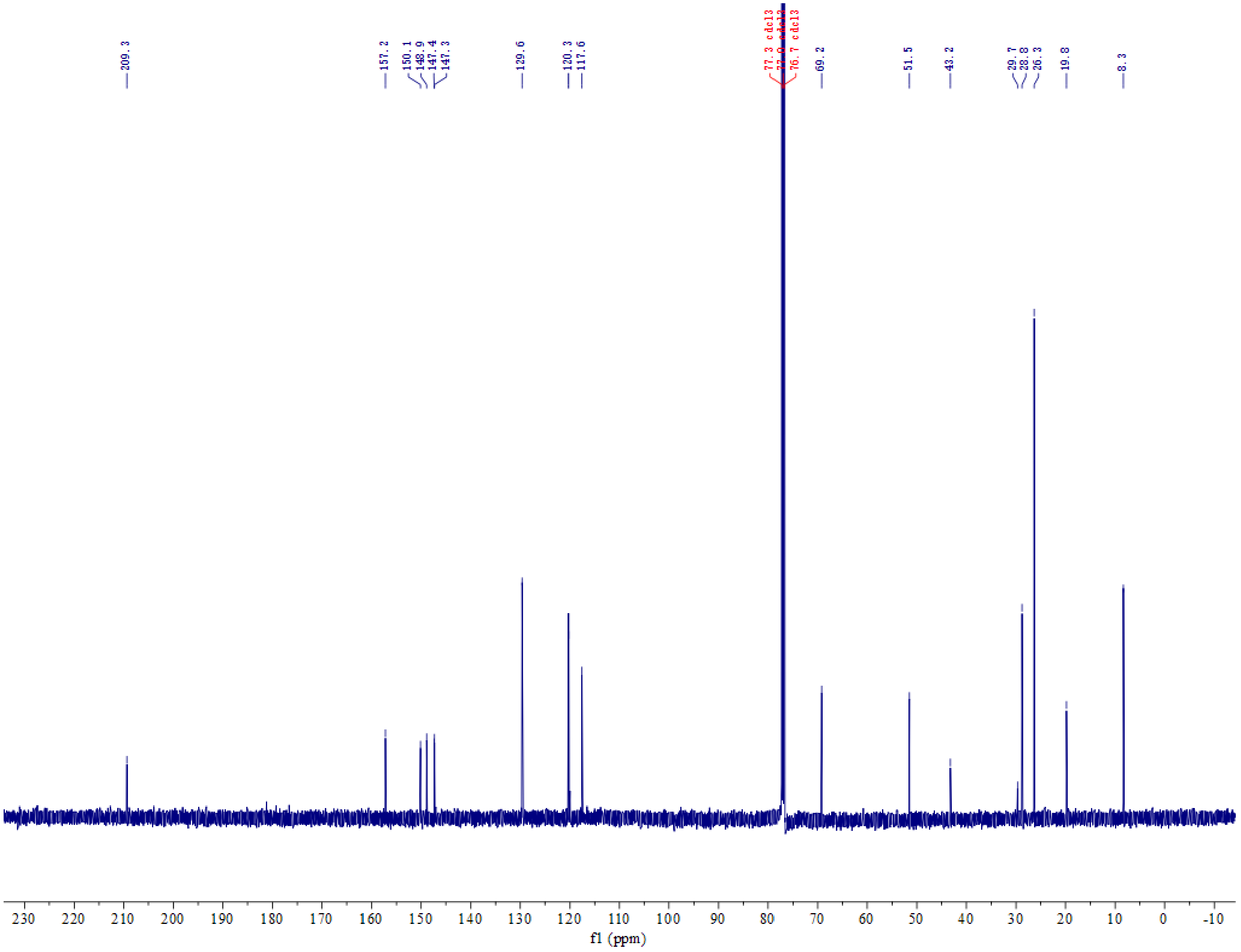

15 HRMS

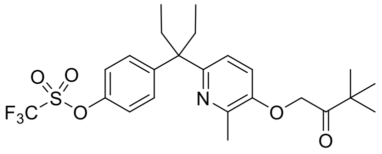

15

210831-02ESI HRMS-Gleason 08/31/21 08:33:00

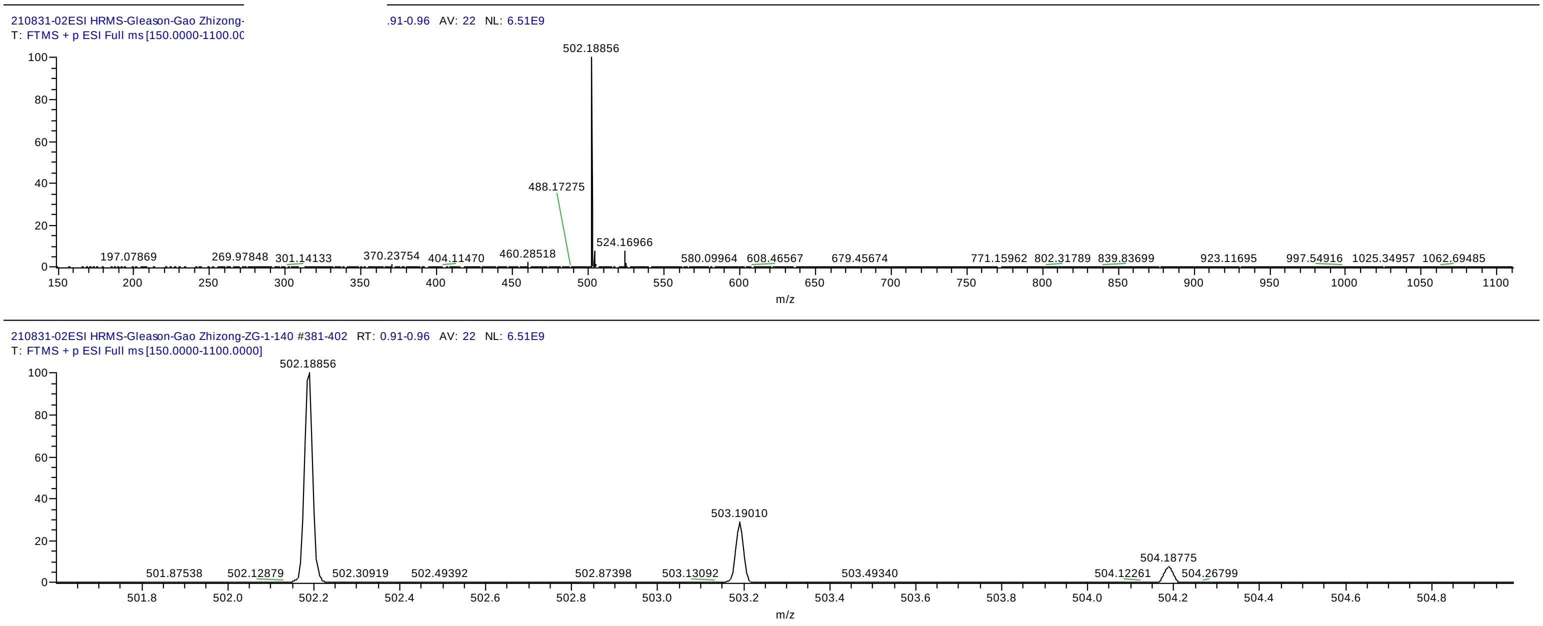

210831-02ESI HRMS-Gleason-Gao Zhizong-ZG-1-140#381-402 RT: 0.91-0.96 AV: 22
T: FTMS + p ESI Full ms [150.0000-1100.0000]
m/z= 502.16269-502.19898

m/z	Intensity	Relative	Resolution	Charge	Theo. Mass	Delta (ppm)	RDB equiv.	Composition
502.18856	6686204416.0	100.00	23390.66	1.00	502.18695	3.20	8.5	$C_{24}H_{31}O_5NF_3S$

16 ^{1}H NMR (500 MHz, $CDCl_3$)

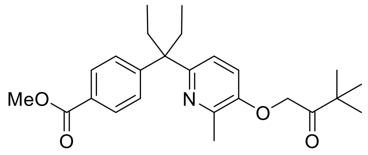

16

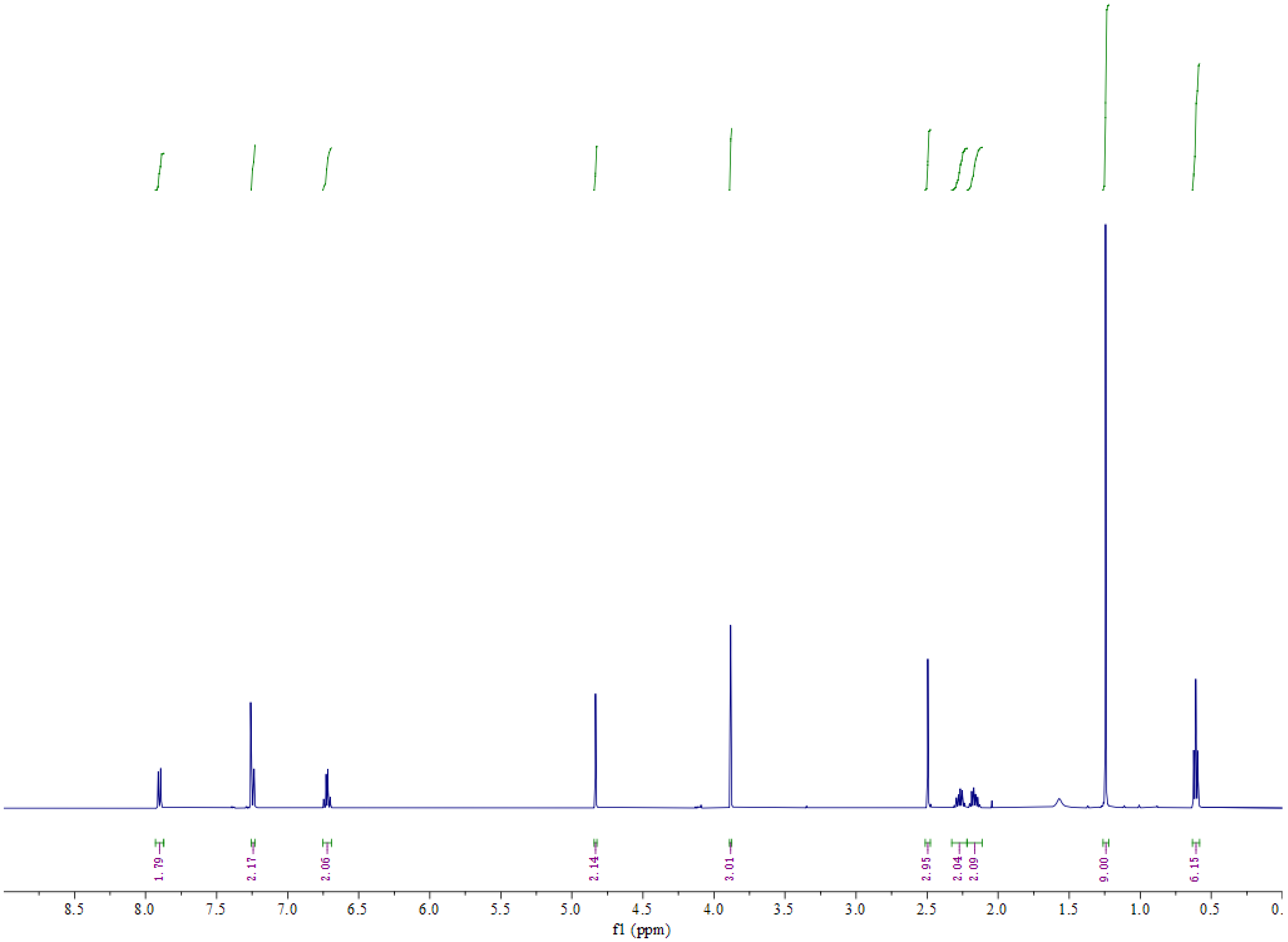

16 ^{13}C NMR (126 MHz, $CDCl_3$)

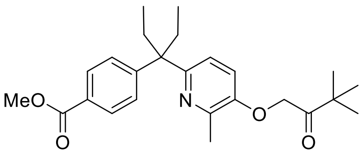

16

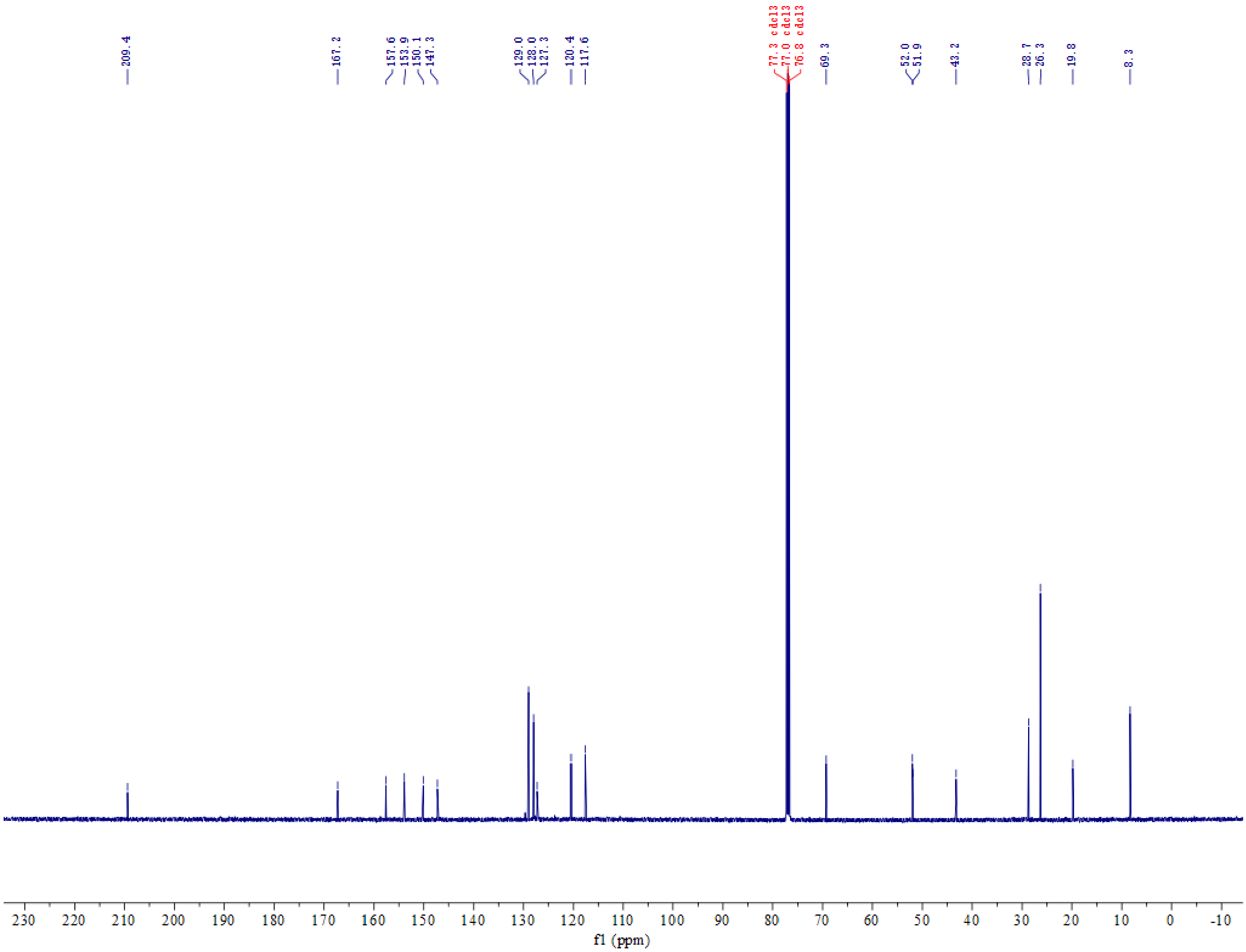

16 HRMS

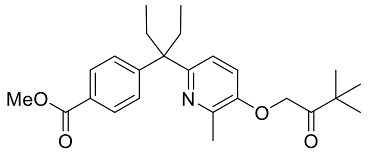

16

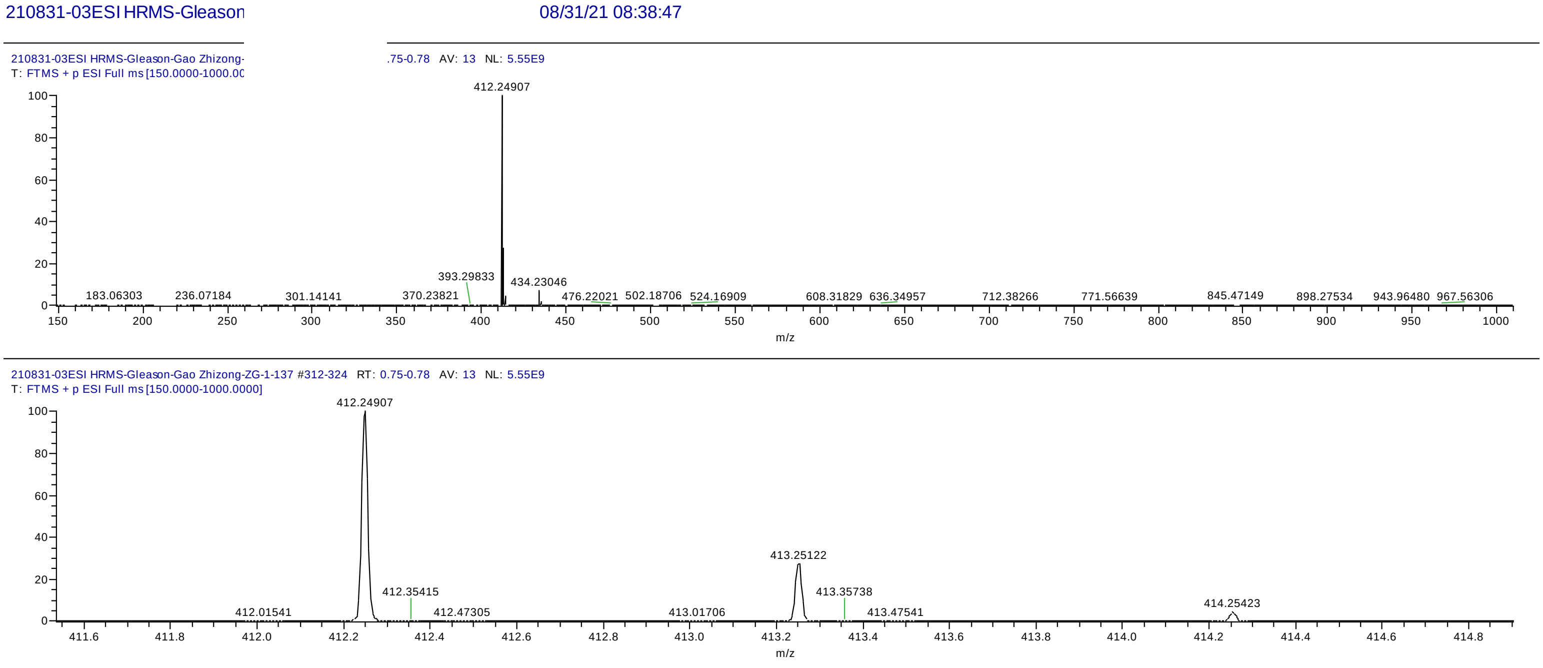

210831-03ESI HRMS-Gleason-Gao Zhizong-ZG-1-137#312-324 RT: 0.75-0.78 AV: 13
T: FTMS + p ESI Full ms [150.0000-1000.0000]
m/z= 412.22785-412.28709

m/z	Intensity	Relative	Resolution	Charge	Theo. Mass	Delta (ppm)	RDB equiv.	Composition
412.24907	5726193664.0	100.00	25840.36	1.00	412.24824	2.02	9.5	$C_{25}H_{34}O_4N$

17 ^{1}H NMR (500 MHz, $CDCl_3$)

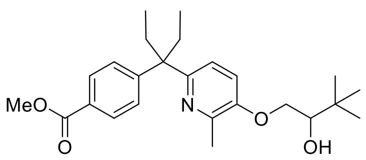

17

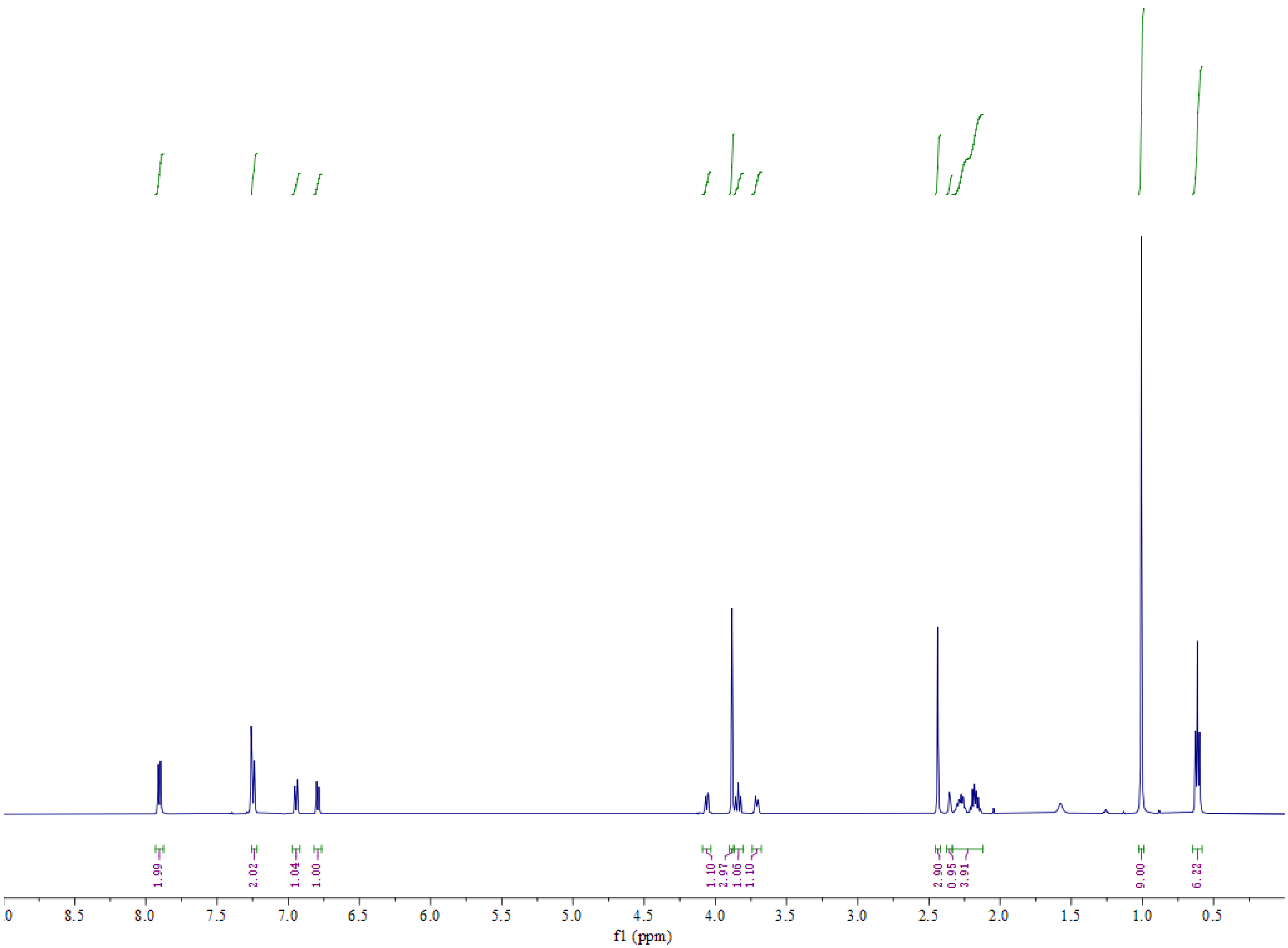

17 ^{13}C NMR (126 MHz, $CDCl_3$)

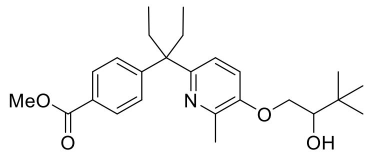

17

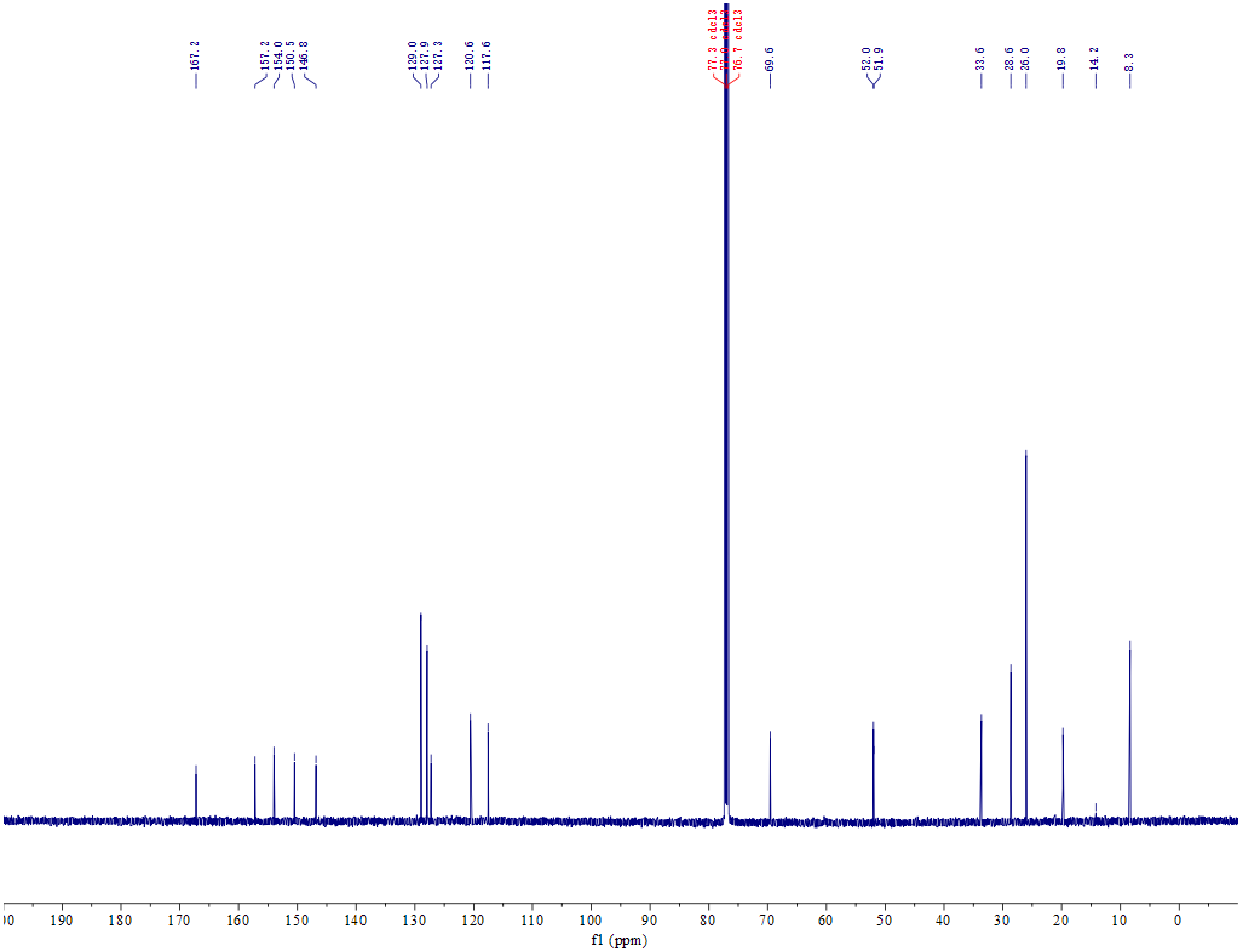

17 HRMS

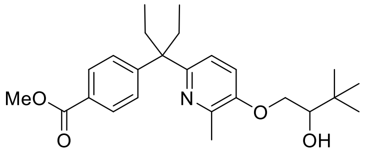

17

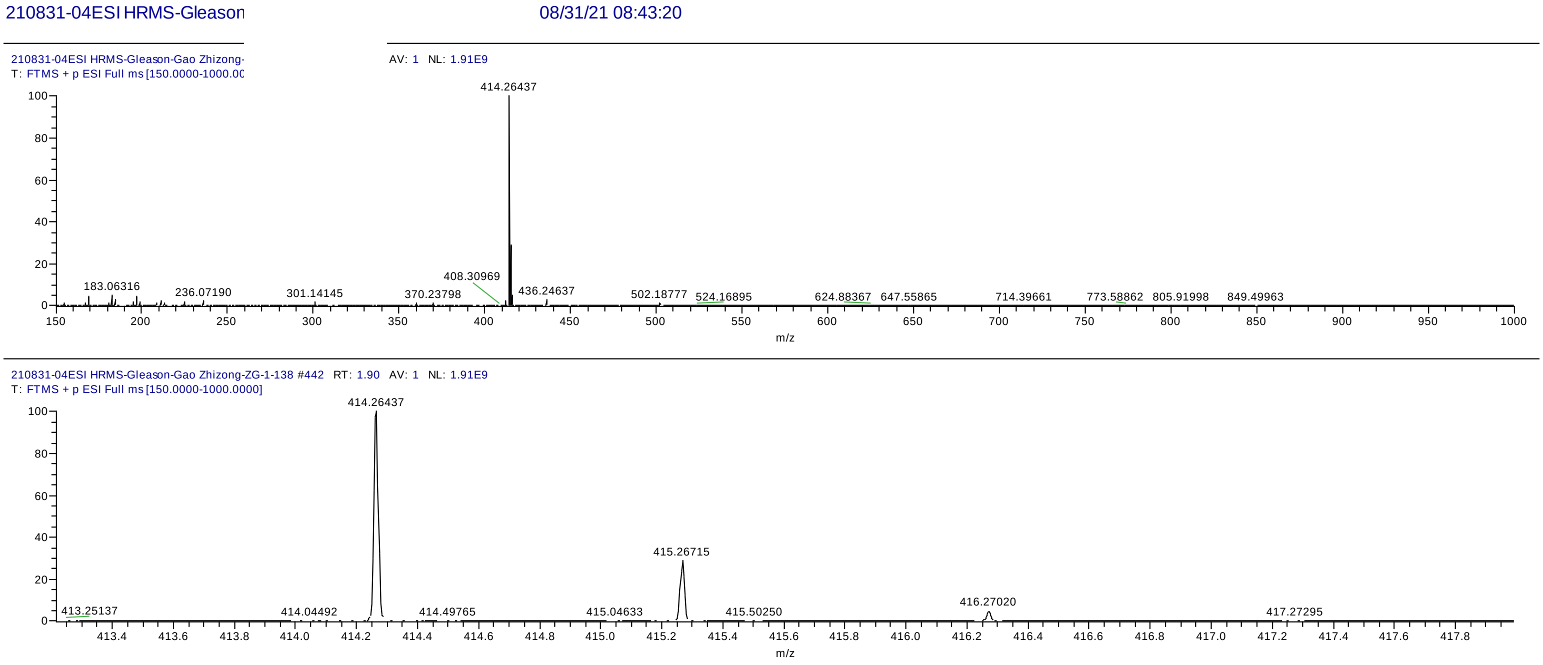

210831-04ESI HRMS-Gleason-Gao Zhizong-ZG-1-138#442 RT: 1.90
T: FTMS + p ESI Full ms [150.0000-1000.0000]
m/z= 414.24104-414.28851

m/z	Intensity	Relative	Resolution	Charge	Theo. Mass	Delta (ppm)	RDB equiv.	Composition
414.26437	2018112000.0	100.00	28206.00	1.00	414.26389	1.18	8.5	$C_{25}H_{36}O_4N$

18 ^{1}H NMR (500 MHz, CD_3OD)

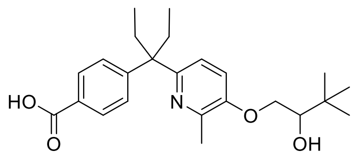

18

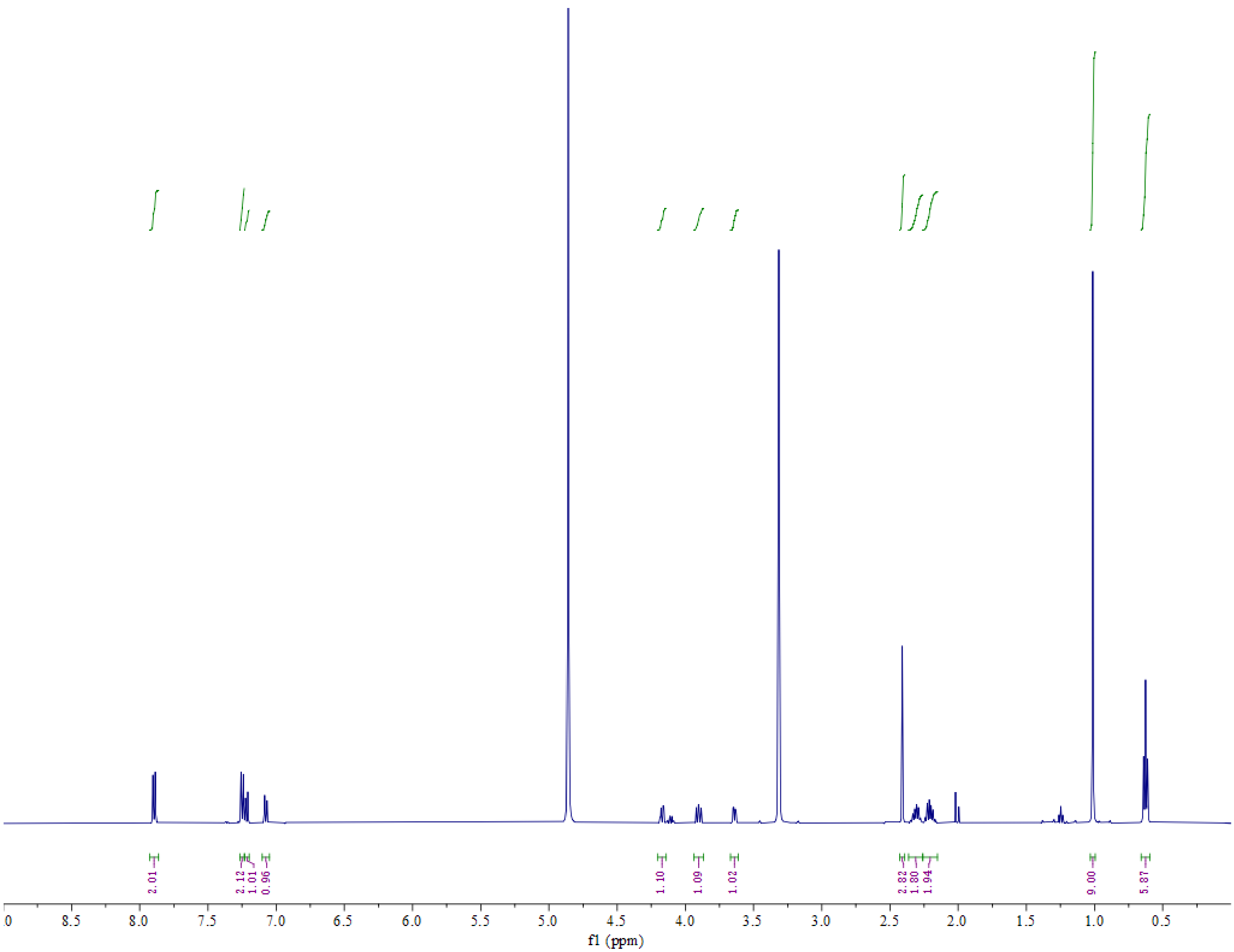

18 ^{13}C NMR (126 MHz, CD_3OD)

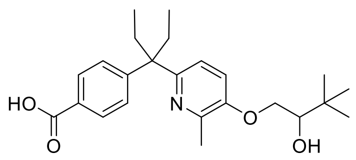

18

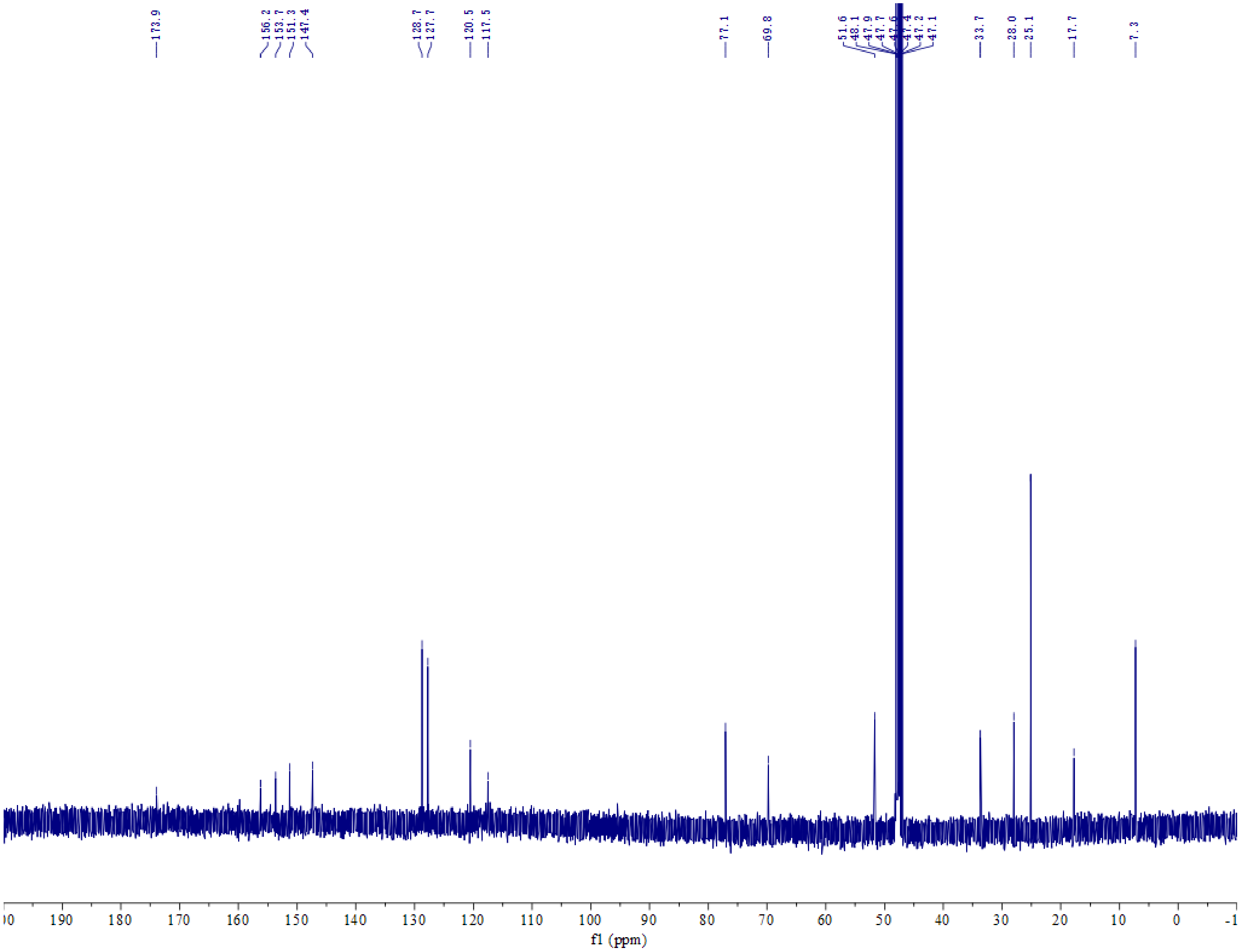

18 HRMS

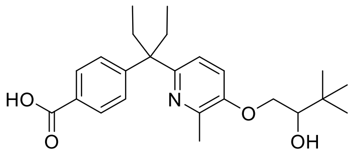

18

210719-01ESI HR-Gleason-Ga   07/19/21 09:08:28

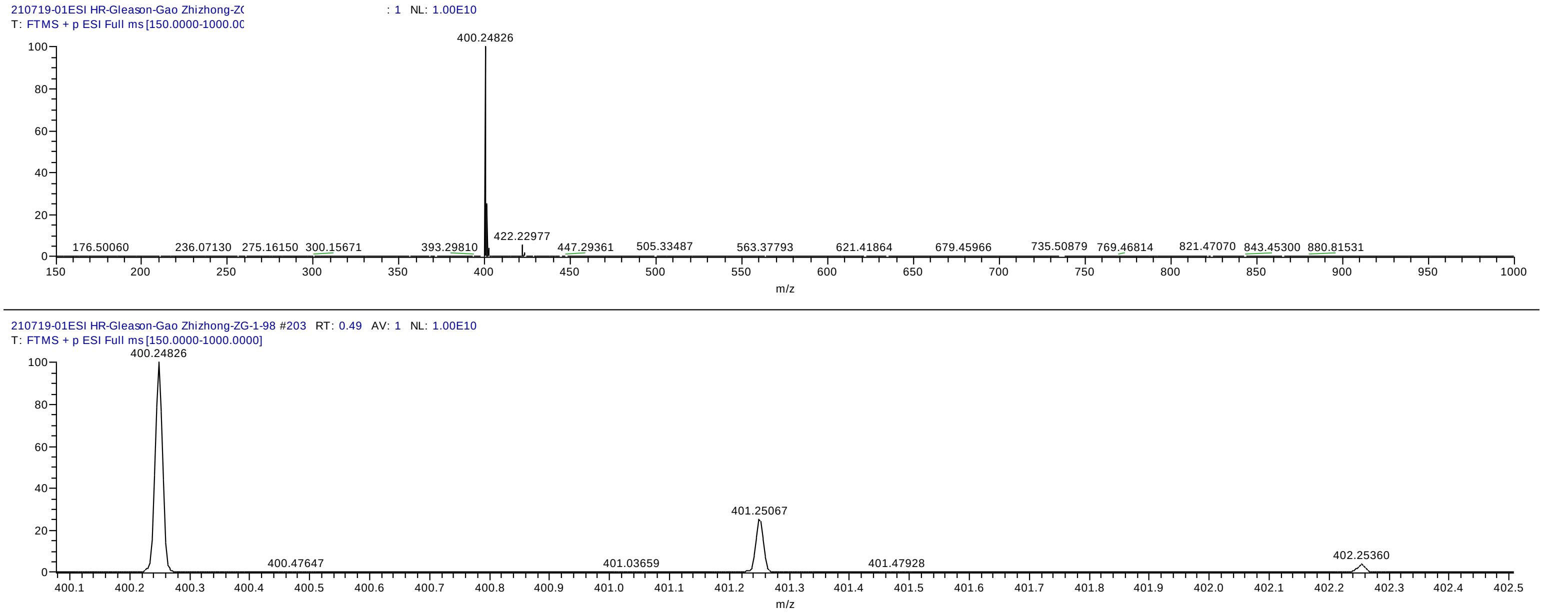

210719-01ESI HR-Gleason-Gao Zhizhong-ZG-1-98#203 RT: 0.49
T: FTMS + p ESI Full ms [150.0000-1000.0000]
m/z= 400.23669-400.26829

m/z	Intensity	Relative	Resolution	Charge	Theo. Mass	Delta (ppm)	RDB equiv.	Composition
400.24826	10009442304.0	100.00	28406.00	1.00	400.24824	0.06	8.5	$C_{24}H_{34}O_4N$

19a ^{1}H NMR (800 MHz, CD_3OD)

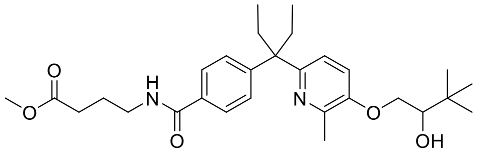

19a

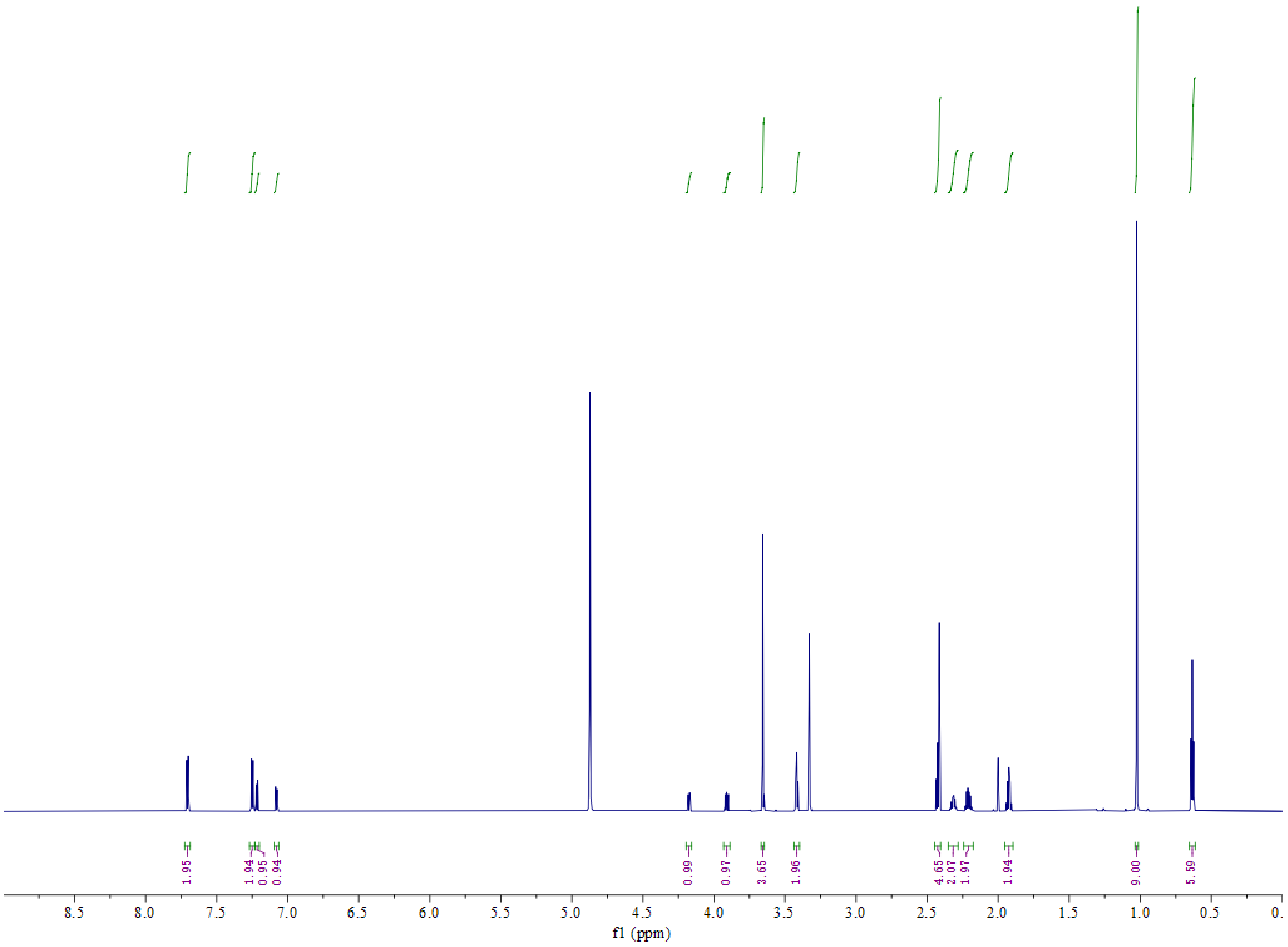

19a ^{13}C NMR (201 MHz, CD_3OD)

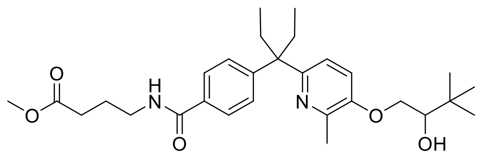

19a

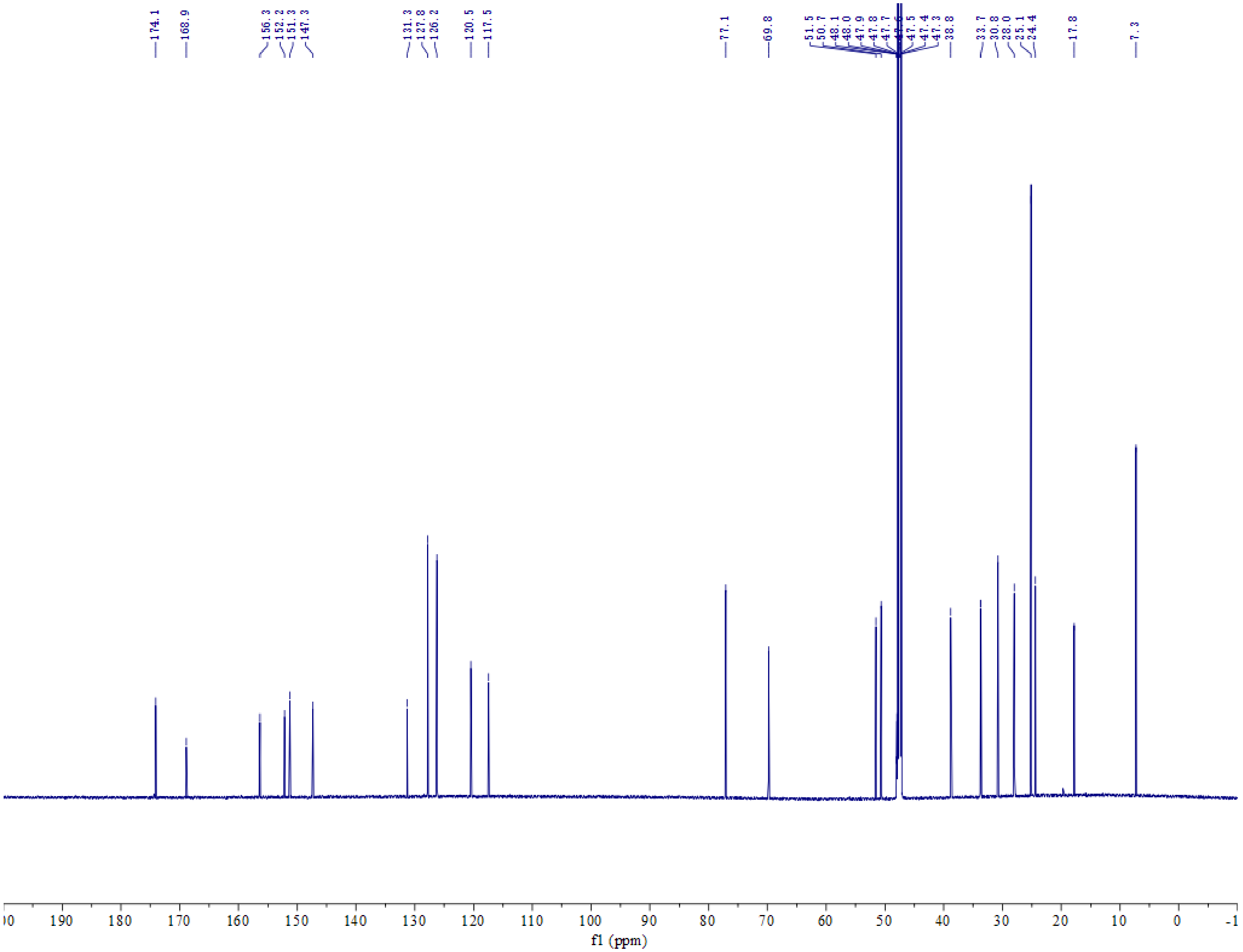

19a HRMS

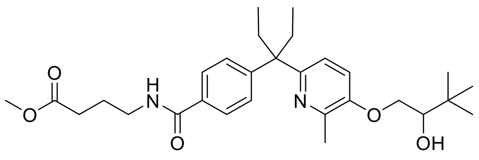

19a

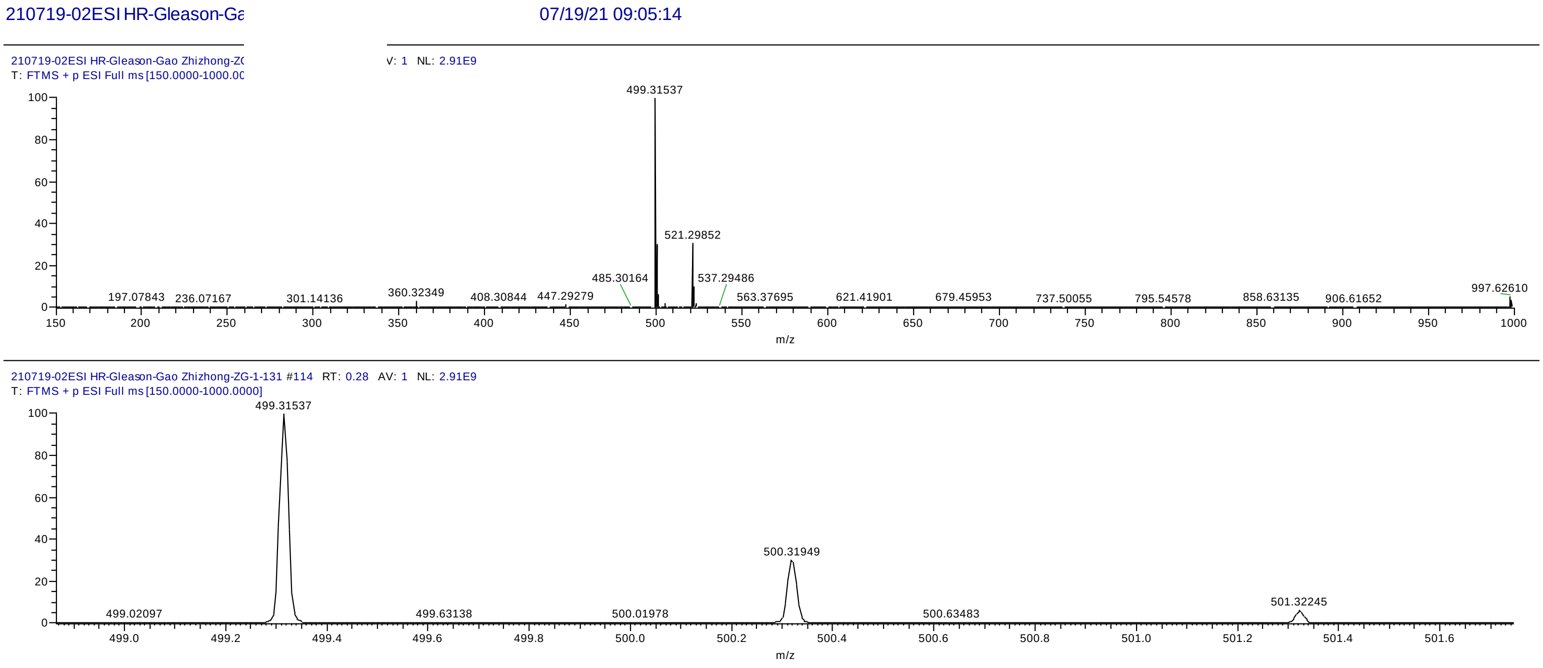

210719-02ESI HR-Gleason-Gao Zhizhong-ZG-1-131#114 RT: 0.28
T: FTMS + p ESI Full ms [150.0000-1000.0000]
m/z= 499.28533-499.33600

m/z	Intensity	Relative	Resolution	Charge	Theo. Mass	Delta (ppm)	RDB equiv.	Composition
499.31537	2914056960.0	100.00	25406.00	1.00	499.31665	-2.56	9.5	$C_{29}H_{43}O_5N_2$

20a (ZG-132) ^{1}H NMR (800 MHz, CD_3OD)

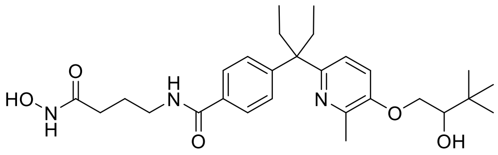

20a (ZG-132)

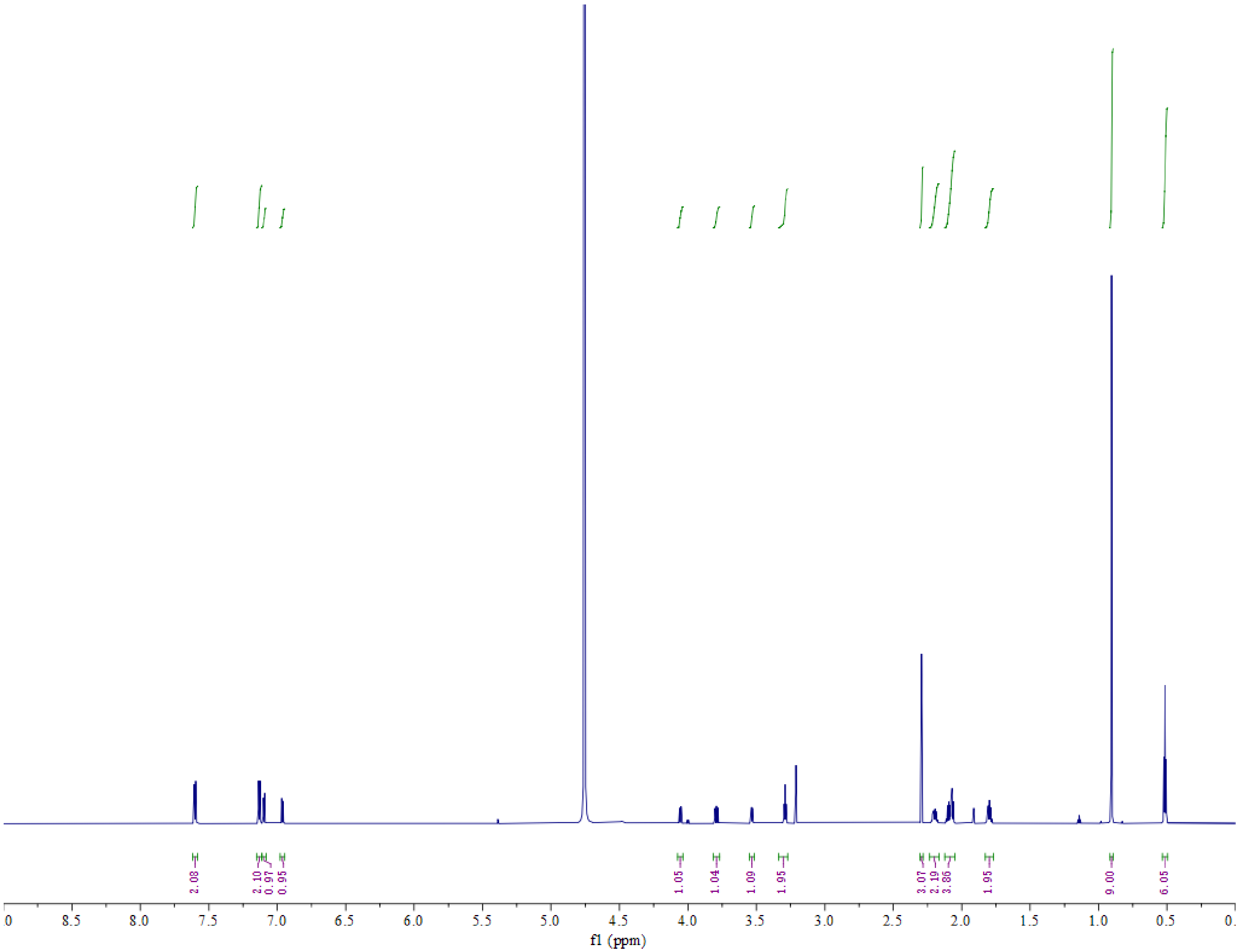

20a (ZG-132) ^{13}C NMR (201 MHz, CD_3OD)

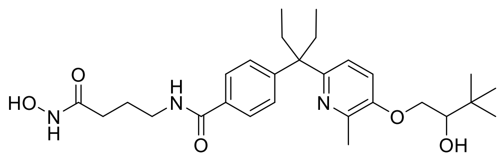

20a (ZG-132)

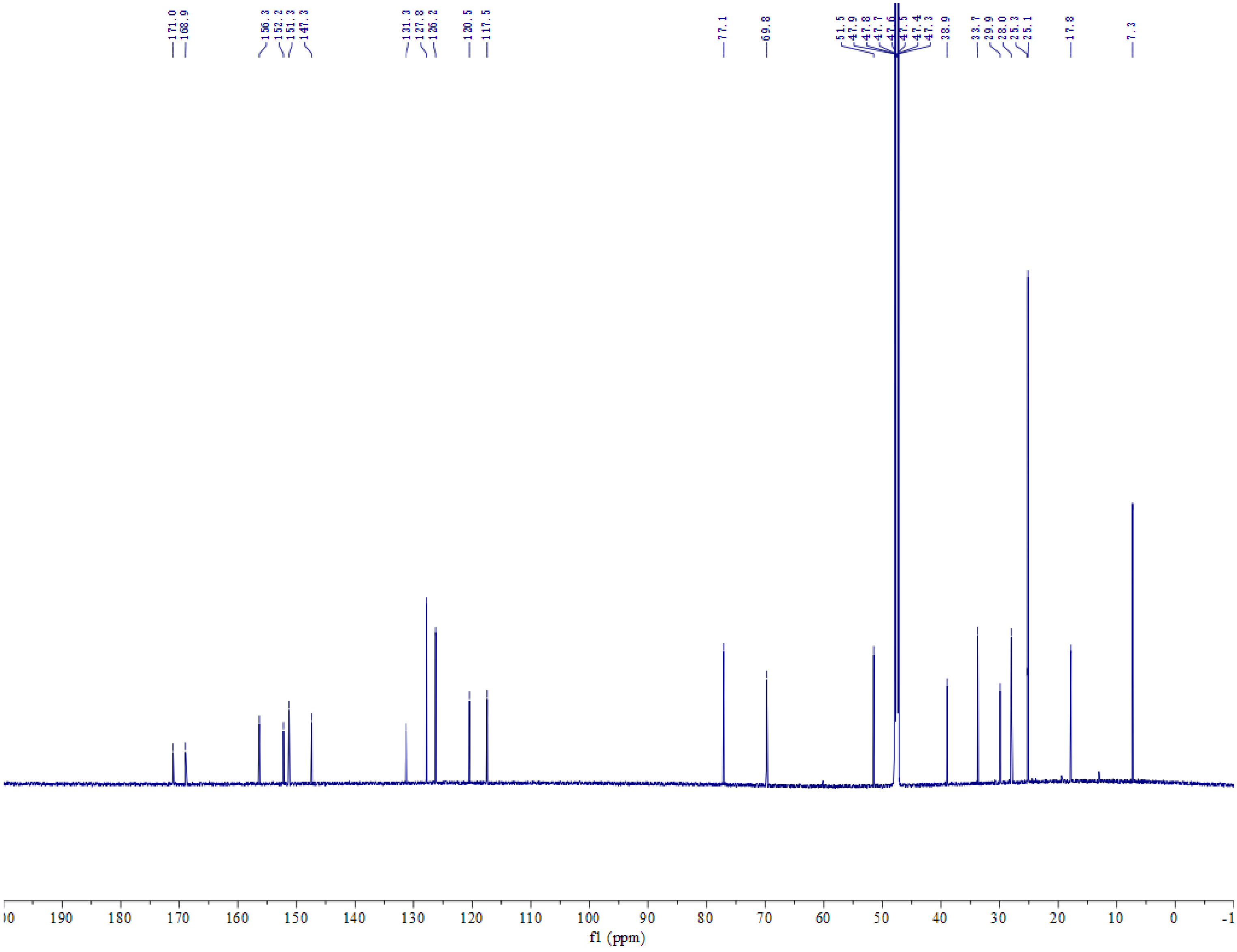

20a (ZG-132) HRMS

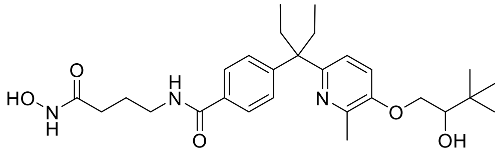

20a (ZG-132)

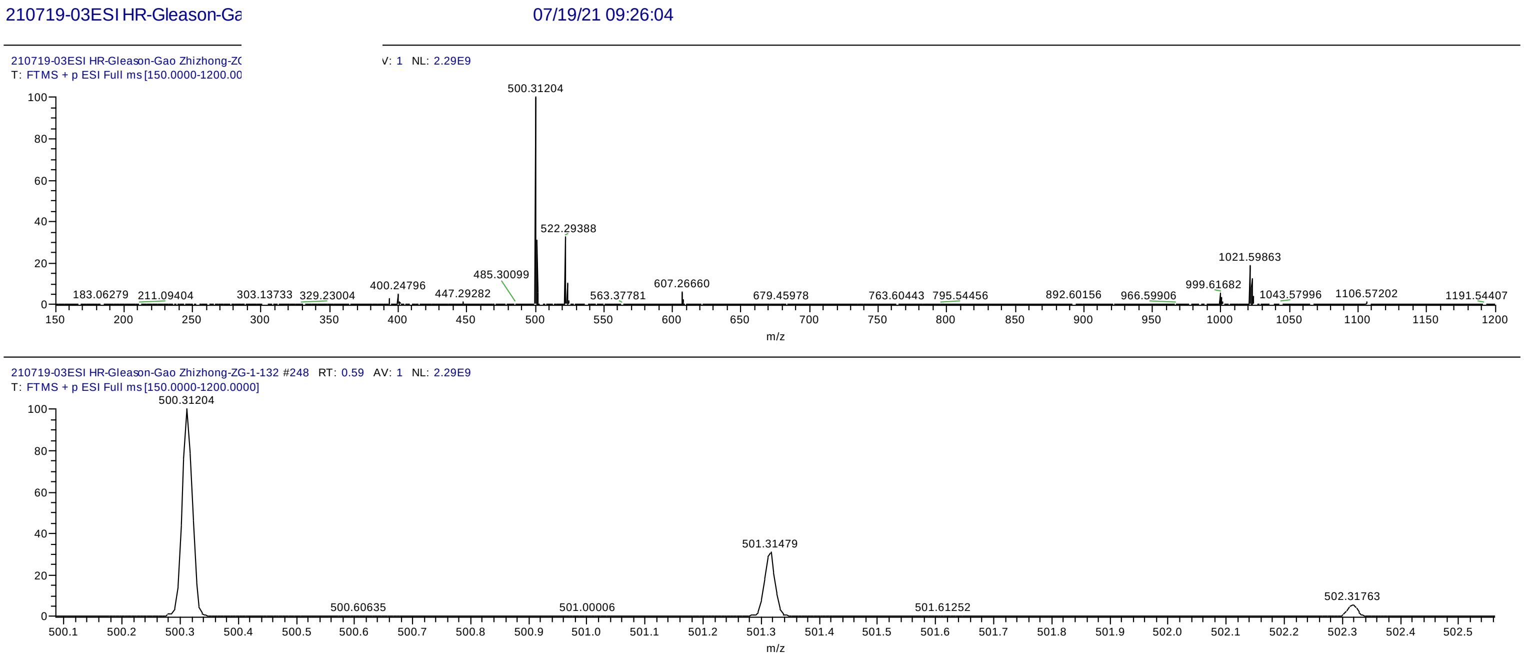

210719-03ESI HR-Gleason-Gao Zhizhong-ZG-1-132#248 RT: 0.59
T: FTMS + p ESI Full ms [150.0000-1200.0000]
m/z= 500.29346-500.32945

m/z	Intensity	Relative	Resolution	Charge	Theo. Mass	Delta (ppm)	RDB equiv.	Composition
500.31204	2289824512.0	100.00	25306.00	1.00	500.31190	0.29	9.5	$C_{28}H_{42}O_5N_3$
					500.31324	-2.40	9.0	$C_{30}H_{44}O_6$

19b ^{1}H NMR (500 MHz, CD_3OD)

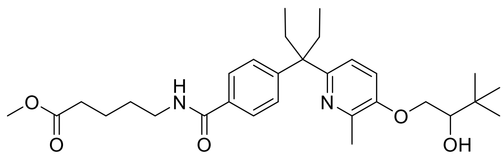

19b

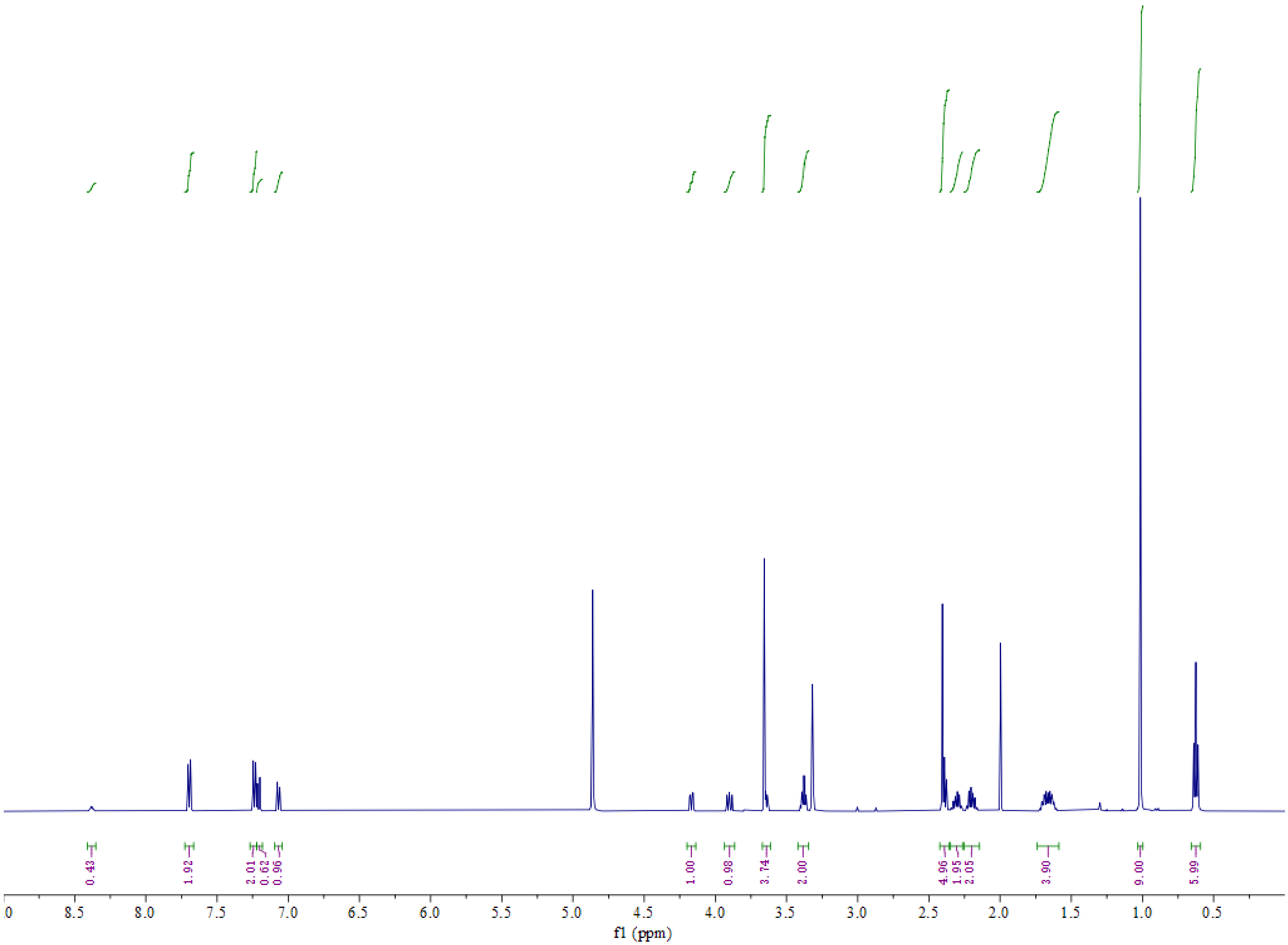

19b ^{13}C NMR (126 MHz, CD_3OD)

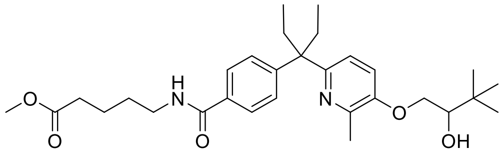

19b

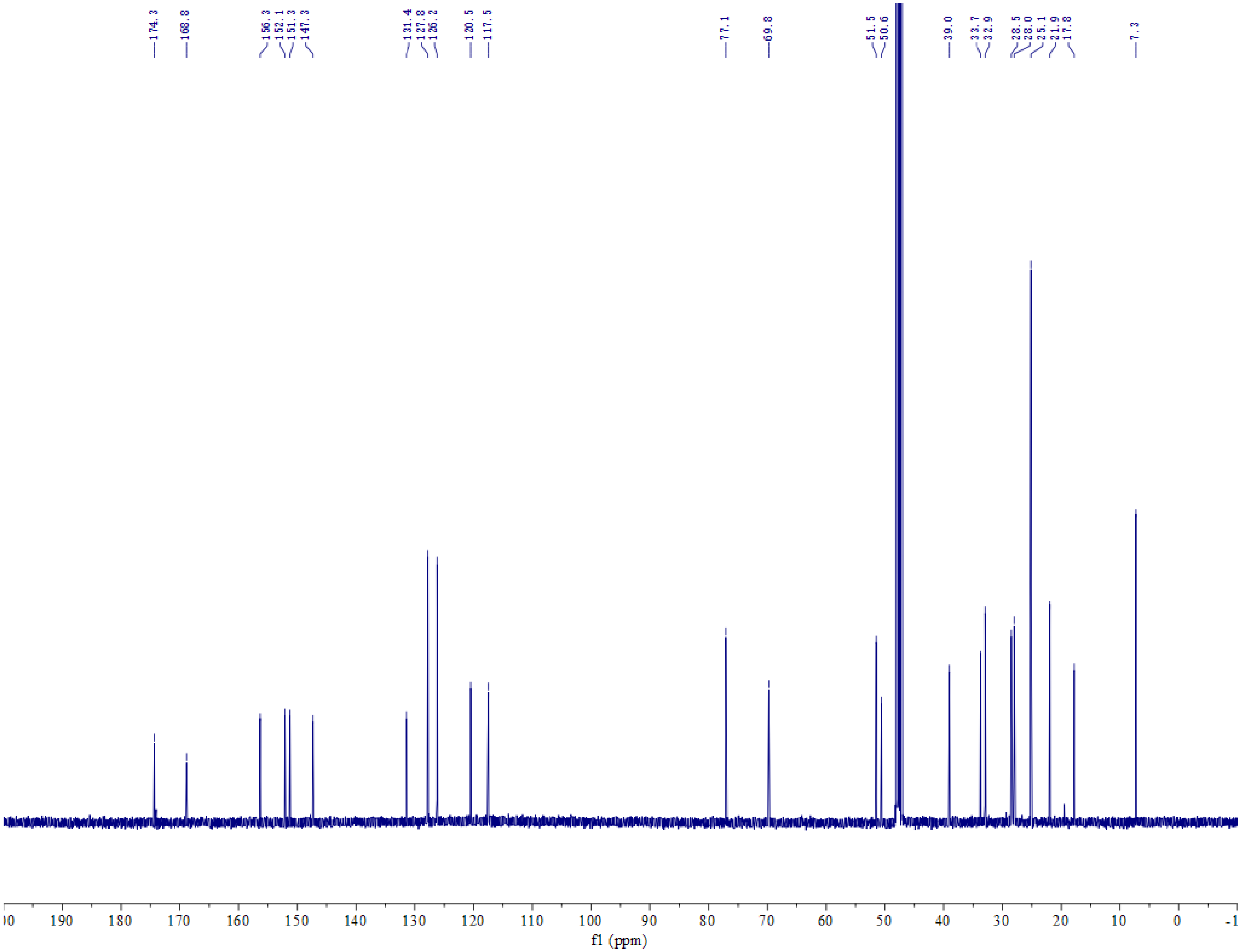

19b HRMS

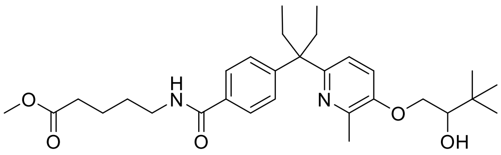

19b

210719-04ESI HR-Gleason-Ga 07/19/21 09:29:37

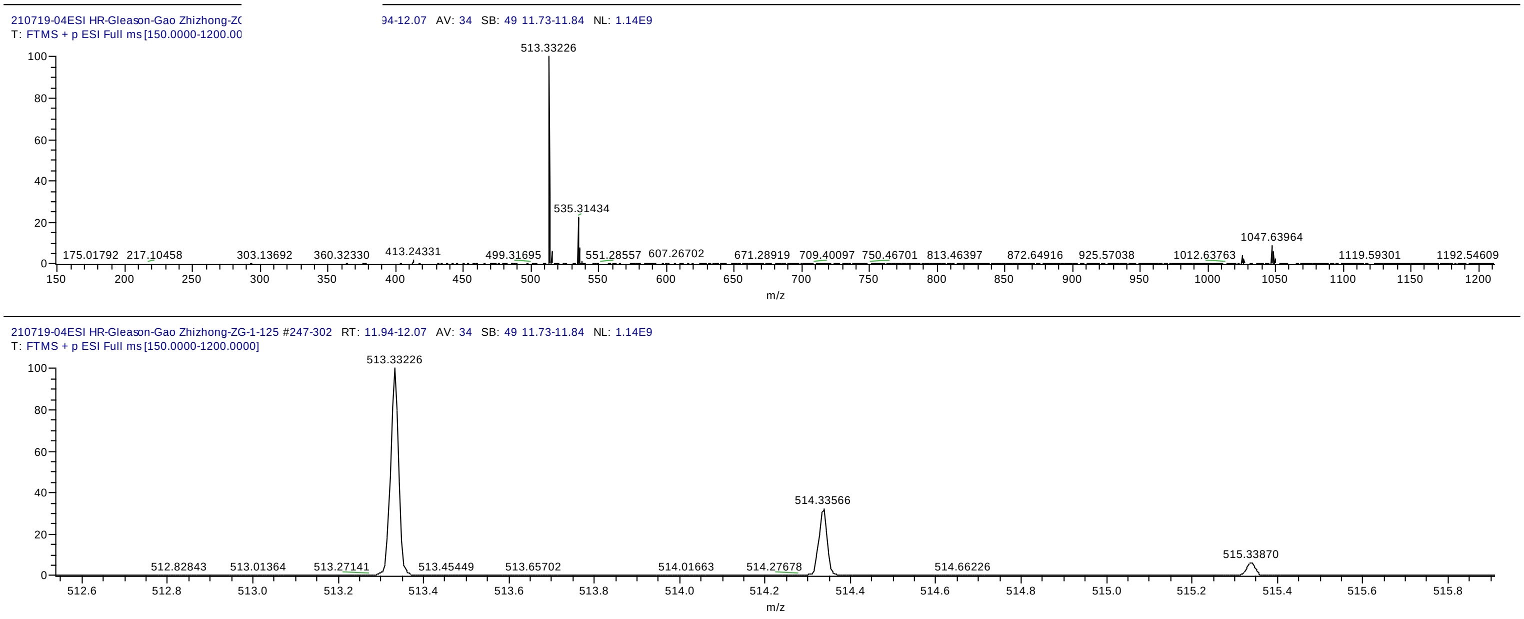

210719-04ESI HR-Gleason-Gao Zhizhong-ZG-1-125#247-302 RT: 11.94-12.07 AV: 34
SB: 49 11.73-11.84
T: FTMS + p ESI Full ms [150.0000-1200.0000]
m/z= 513.30561-513.35976

m/z	Intensity	Relative	Resolution	Charge	Theo. Mass	Delta (ppm)	RDB equiv.	Composition
513.33226	1139266048.0	100.00	24307.41	1.00	513.33230	-0.08	9.5	$C_{30}H_{45}O_5N_2$

20b (ZG-126) ^{1}H NMR (800 MHz, CD_3OD)

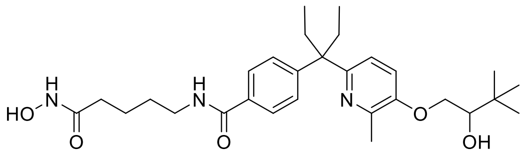

20b (ZG-126)

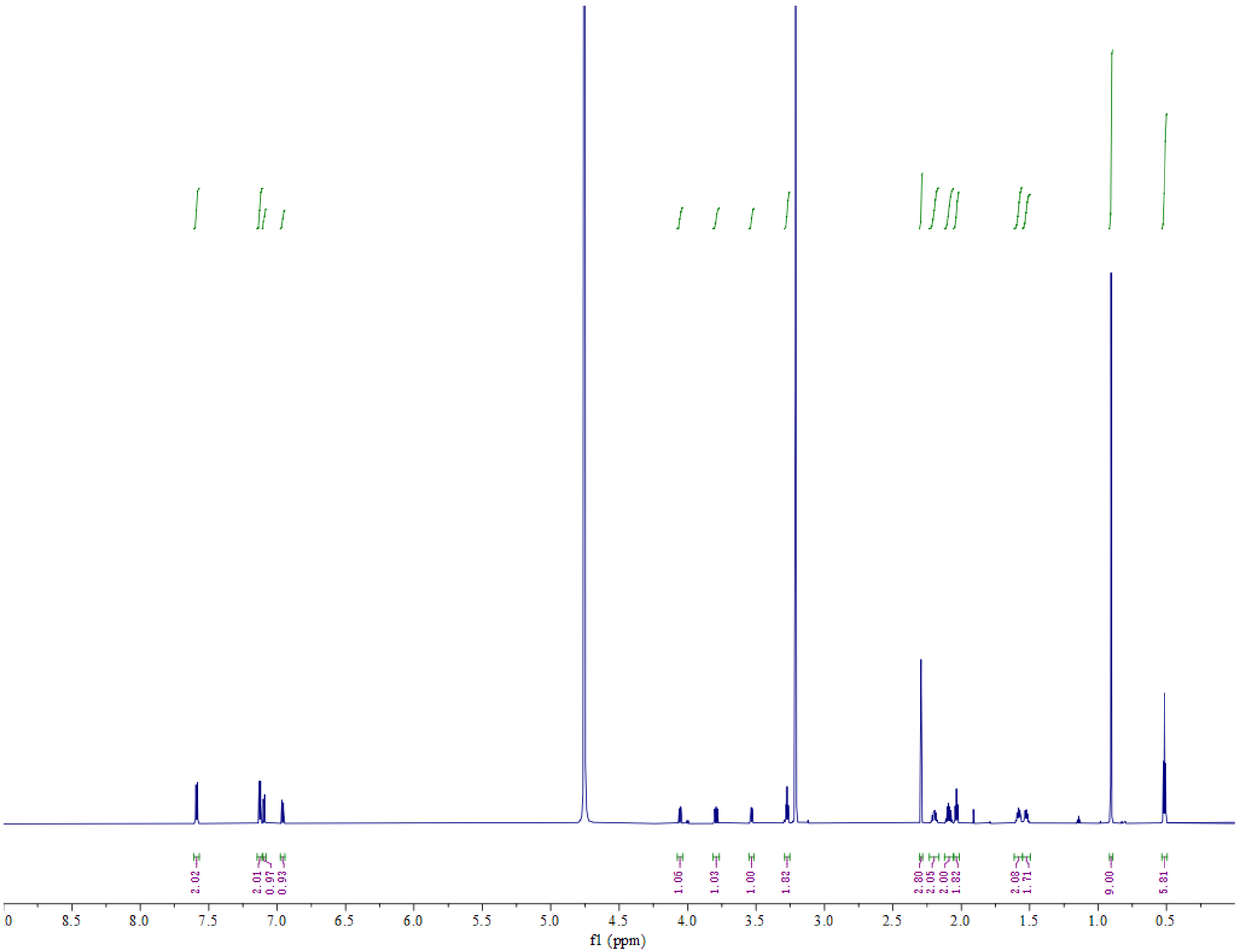

20b (ZG-126) ^{13}C NMR (201 MHz, CD_3OD)

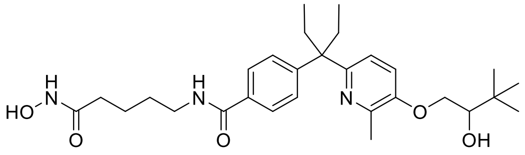

20b (ZG-126)

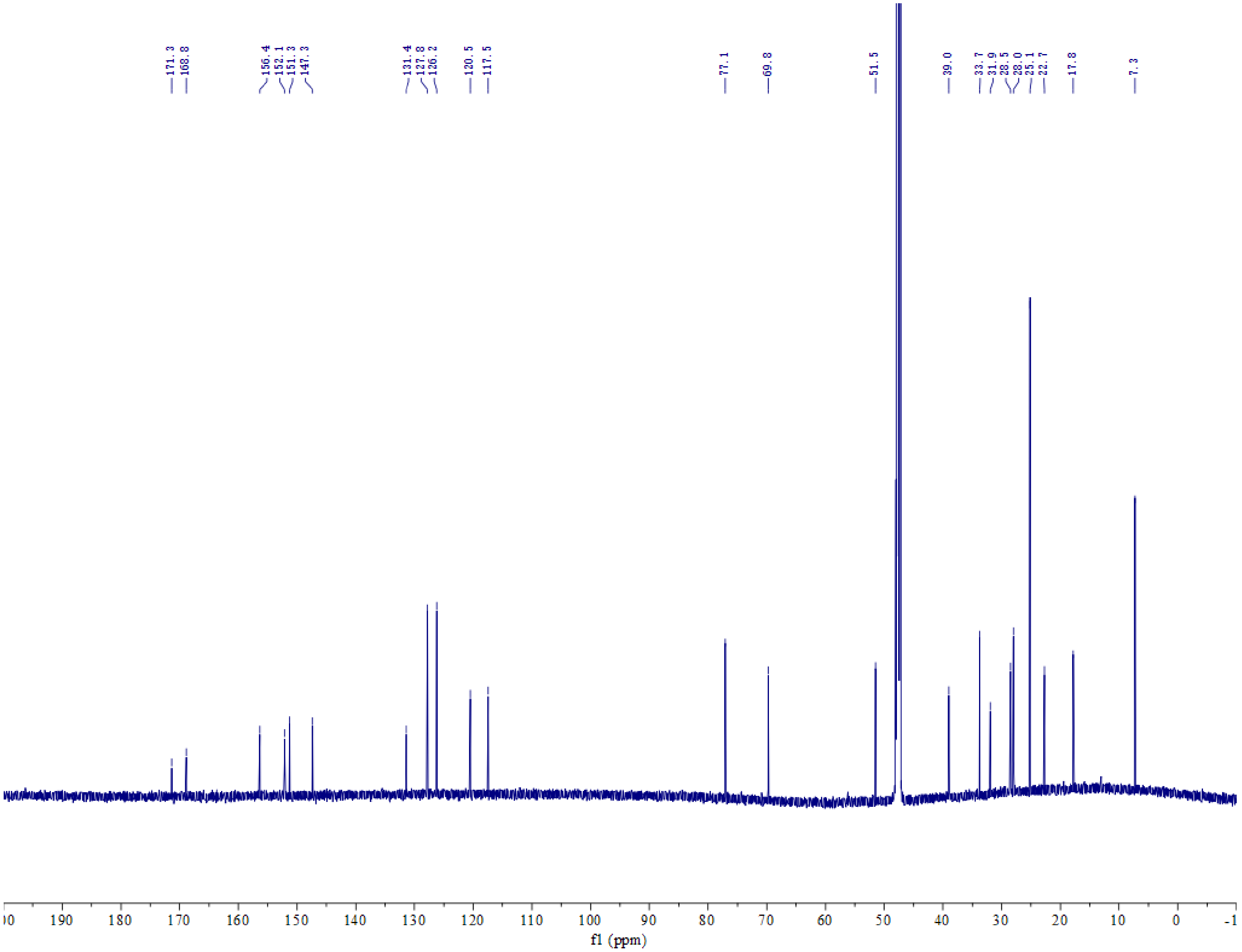

20b (ZG-126) HRMS

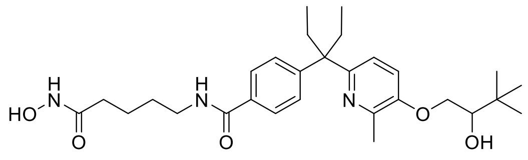

20b (ZG-126)

210719-05ESI HR-Gleason-Ga 07/19/21 09:56:30

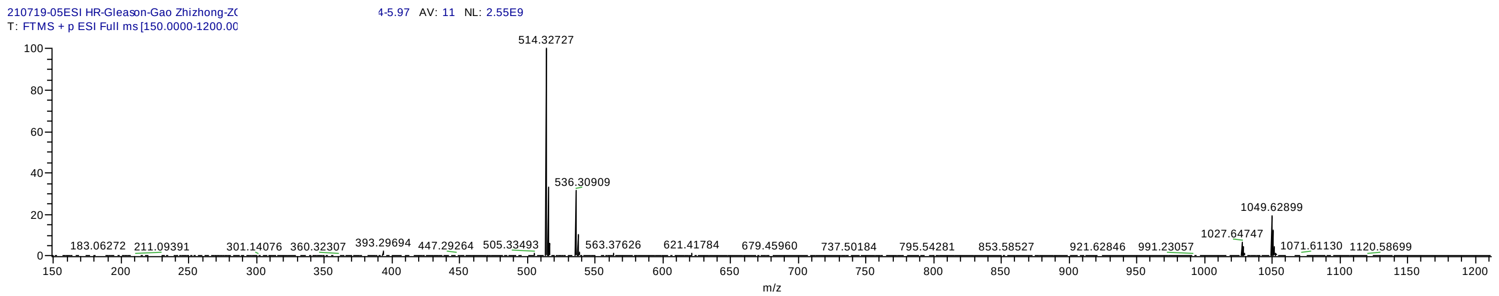

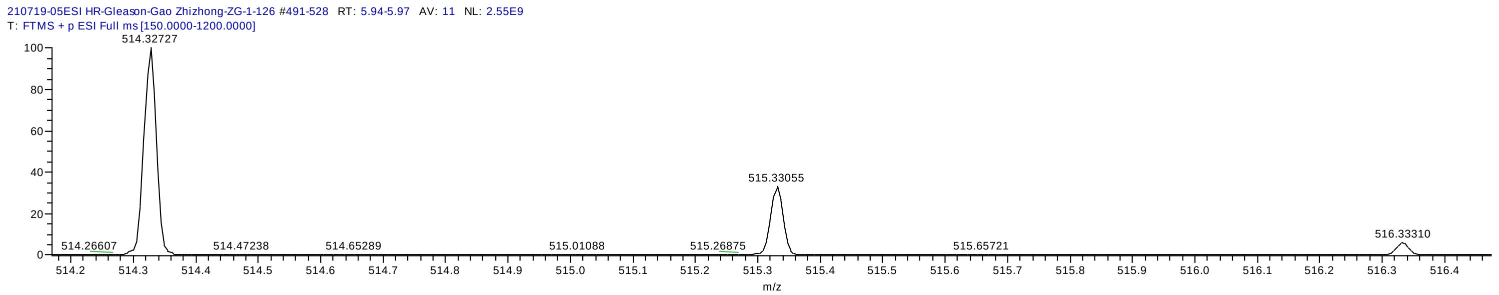

210719-05ESI HR-Gleason-Gao Zhizhong-ZG-1-126#491-501 RT: 5.94-5.97 AV: 11
T: FTMS + p ESI Full ms [150.0000-1200.0000]
m/z= 514.29940-514.34701

m/z	Intensity	Relative	Resolution	Charge	Theo. Mass	Delta (ppm)	RDB equiv.	Composition
514.32727	2557204224.0	100.00	23651.71	1.00	514.32755	-0.54	9.5	$C_{29}H_{44}O_5N_3$
					514.32889	-3.15	9.0	$C_{31}H_{46}O_6$

19c ^{1}H NMR (800 MHz, CD_3OD)

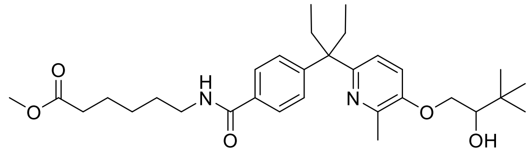

19c

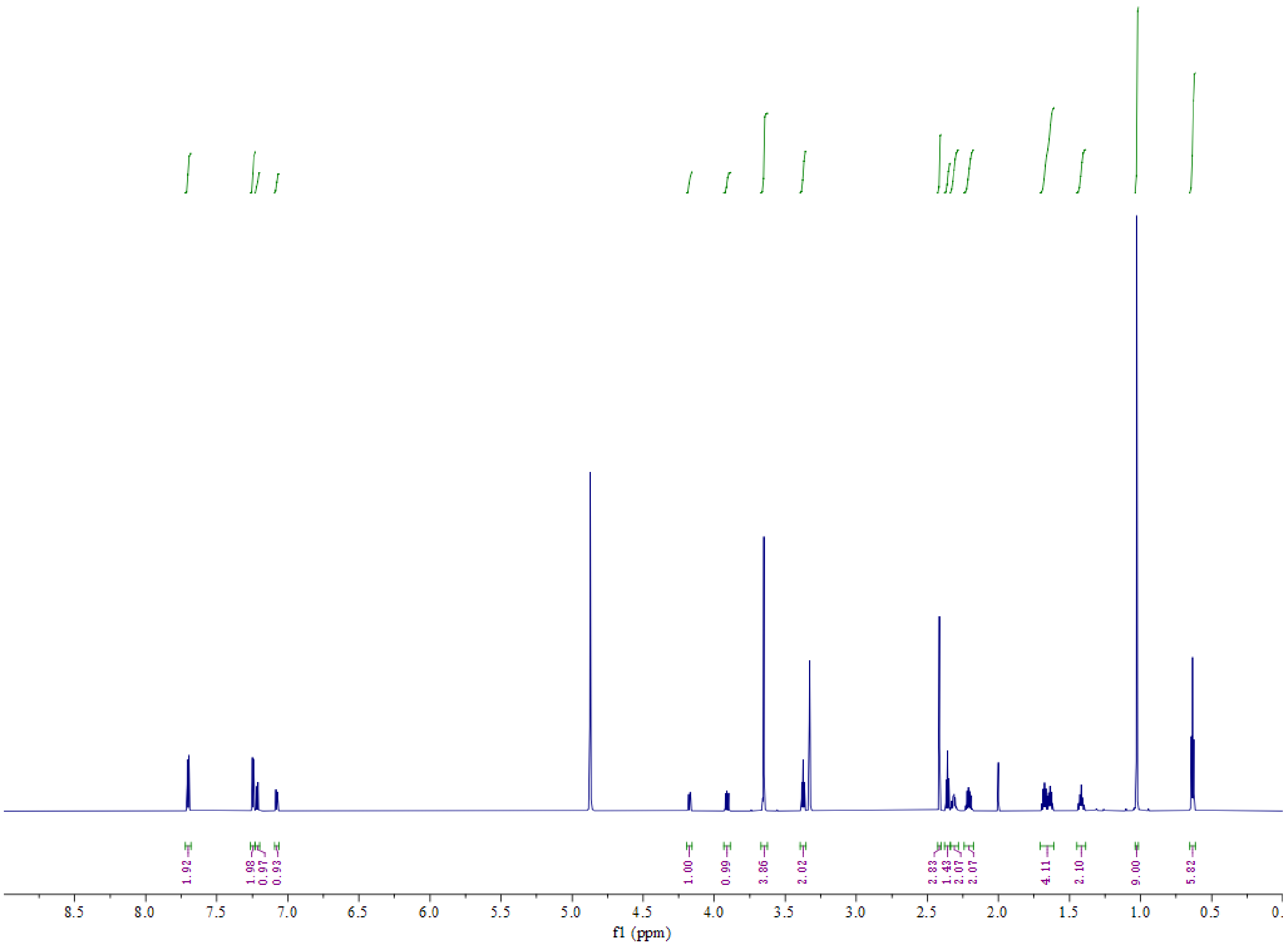

19c ^{13}C NMR (201 MHz, CD_3OD)

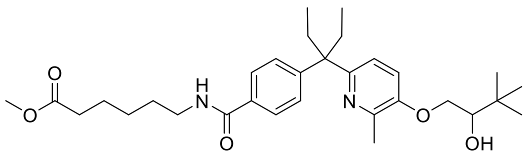

19c

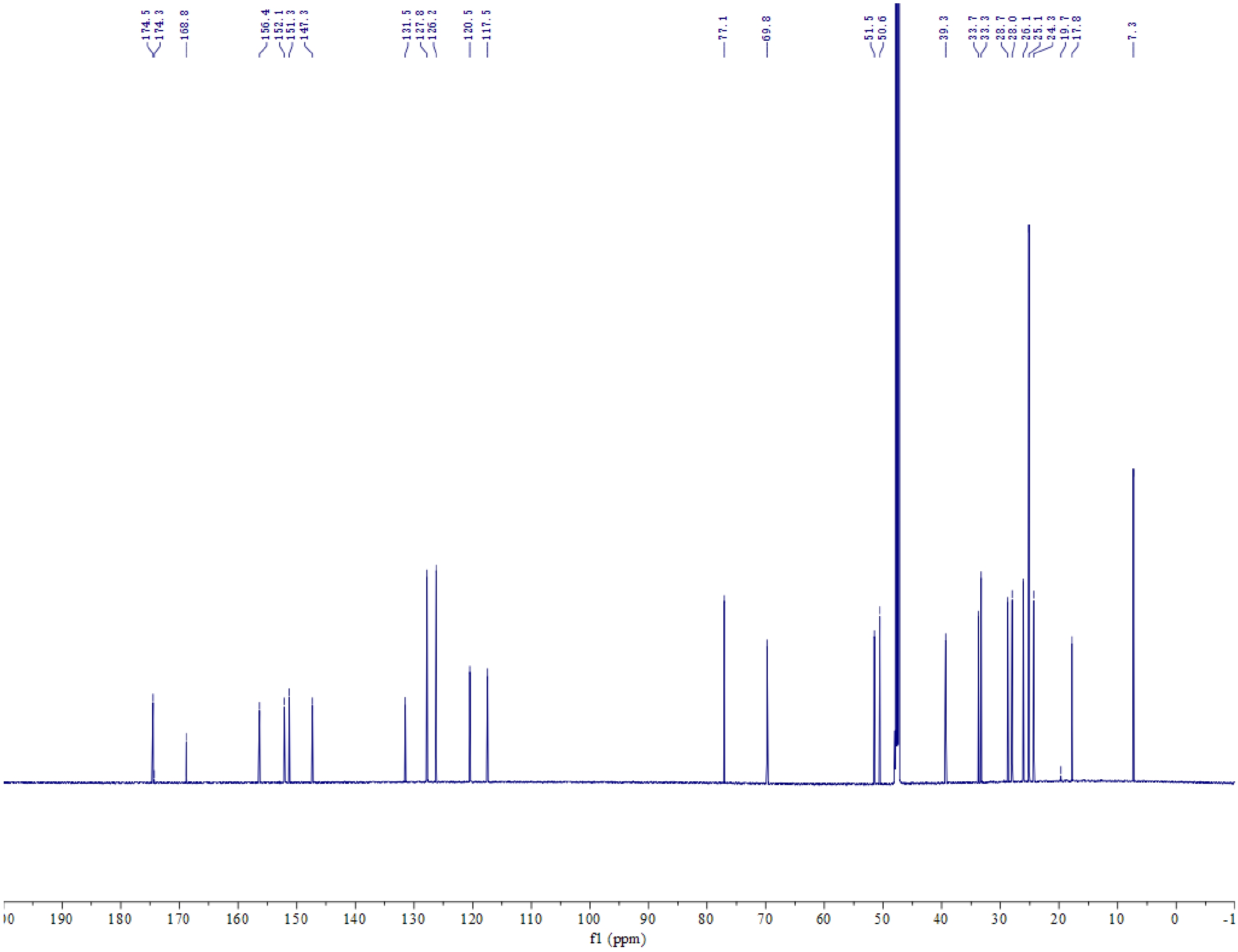

19c HRMS

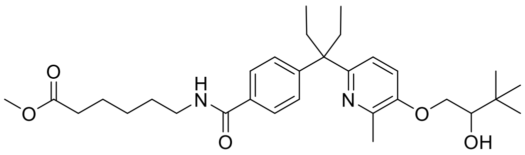

19c

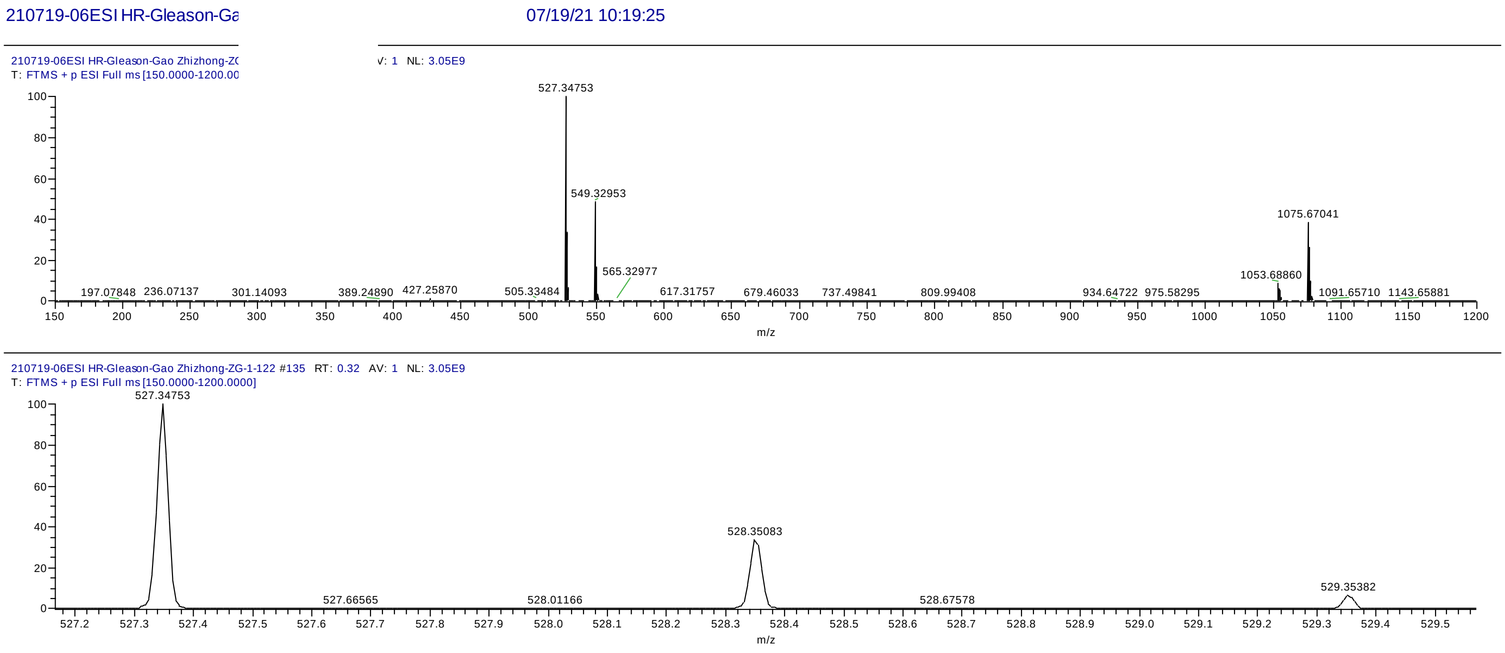

210719-06ESI HR-Gleason-Gao Zhizhong-ZG-1-122#135 RT: 0.32
T: FTMS + p ESI Full ms [150.0000-1200.0000]
m/z= 527.32714-527.37125

m/z	Intensity	Relative	Resolution	Charge	Theo. Mass	Delta (ppm)	RDB equiv.	Composition
527.34753	3057937920.0	100.00	24706.00	1.00	527.34795	-0.79	9.5	$C_{31} H_{47} O_5 N_2$

20c (ZG-102) ^{1}H NMR (800 MHz, CD_3OD)

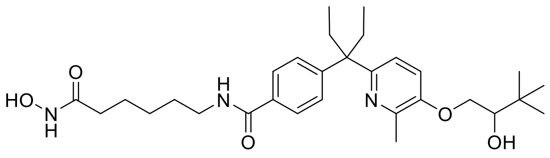

20c (ZG-102)

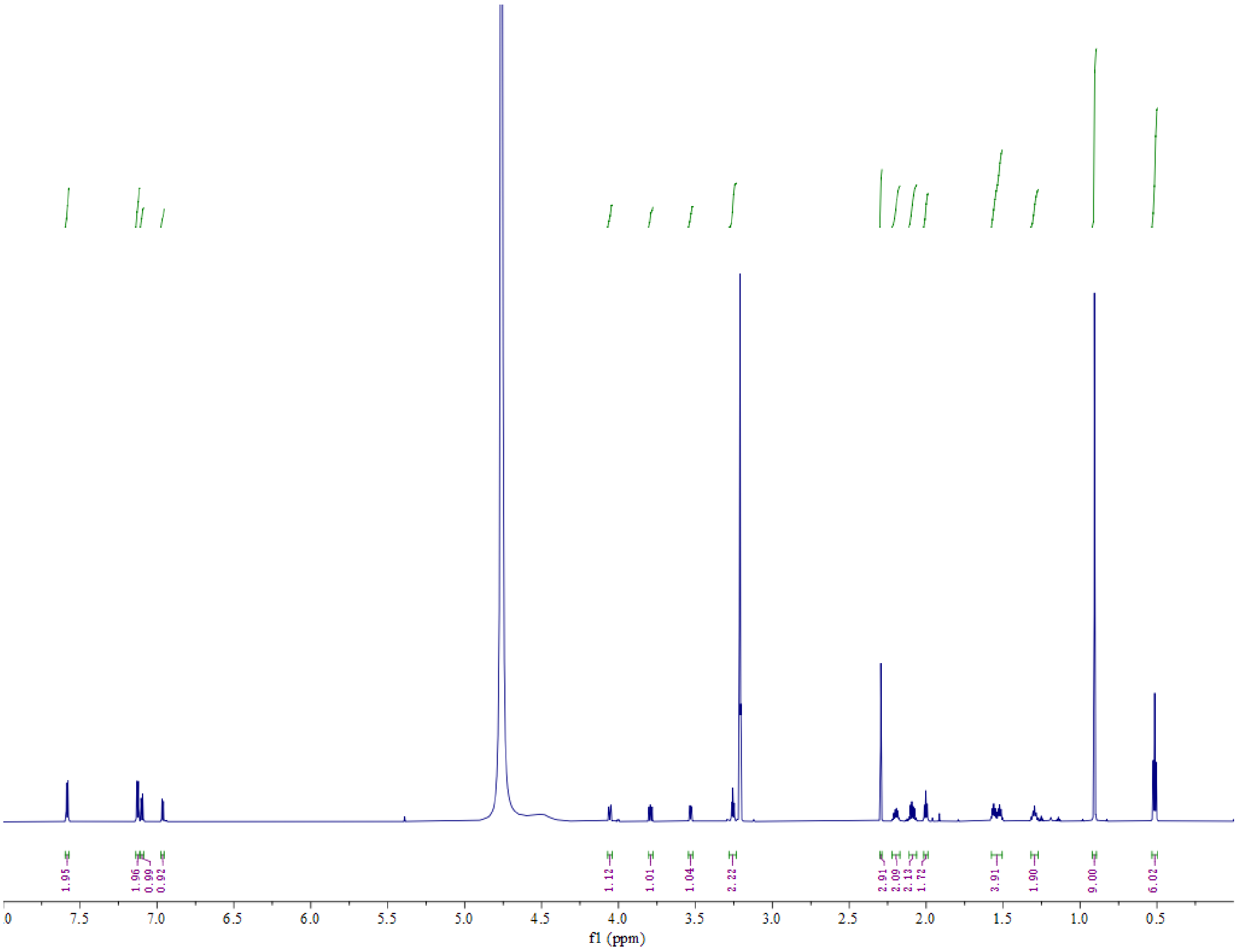

20c (ZG-102) ^{13}C NMR (201 MHz, CD_3OD)

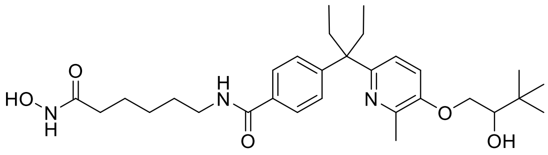

20c (ZG-102)

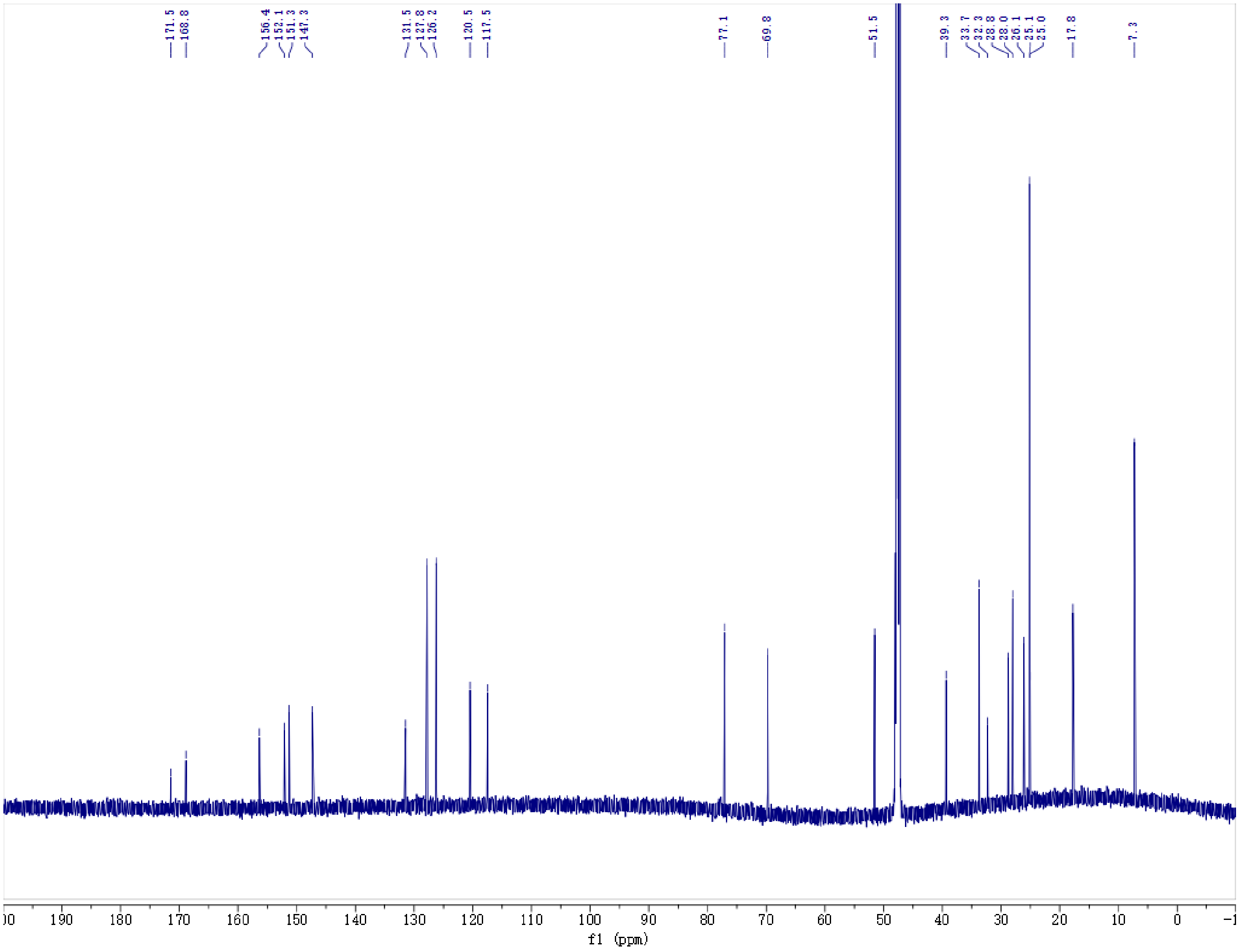

20c (ZG-102) HRMS

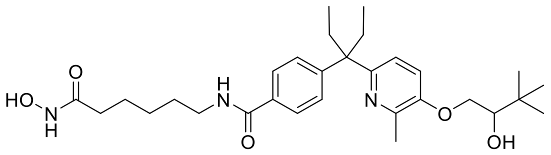

20c (ZG-102)

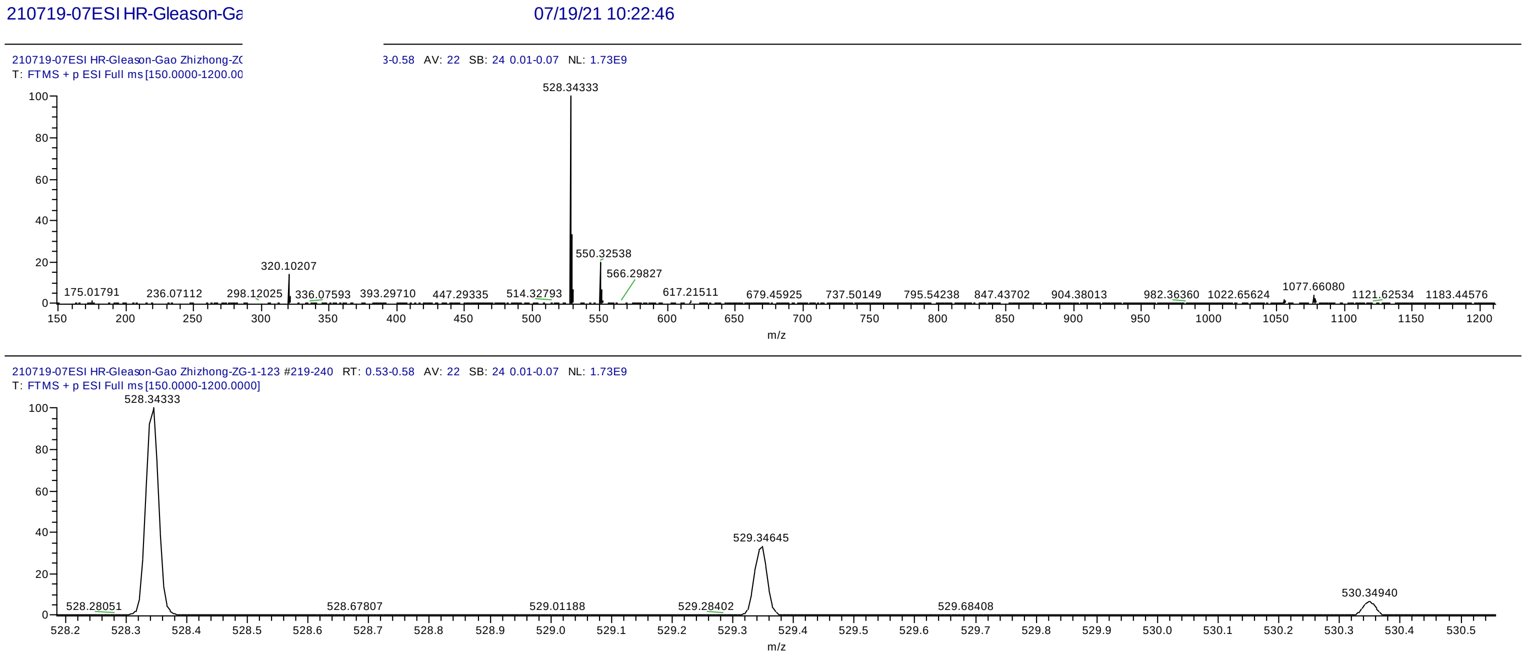

210719-07ESI HR-Gleason-Gao Zhizhong-ZG-1-123#219-240 RT: 0.53-0.58 AV: 22
SB: 24 0.01-0.07
T: FTMS + p ESI Full ms [150.0000-1200.0000]
m/z= 528.31763-528.37205

m/z	Intensity	Relative	Resolution	Charge	Theo. Mass	Delta (ppm)	RDB equiv.	Composition
528.34333	1752032256.0	100.00	22918.21	1.00	528.34320	0.25	9.5	$C_{30}H_{46}O_5N_3$
					528.34454	-2.29	9.0	$C_{32}H_{48}O_6$

ZG-123 = ZG-102

HPLC reports of 20a (ZG-132), 20b (ZG-126), and 20c (ZG-102)

HPLC report of **20a** (ZG-132)

Data File C:\CHEM32\...HIZHONG\ZG SOLUBILITY TEST 6SPOTS X3 2023-08-14 19-48-10\021-2001.D
Sample Name: ZG-132 0.684

===

Acq. Operator : SYSTEM   Seq. Line : 20
Acq. Instrument : 1200LC   Location : Vial 21
Injection Date : 15/08/2023 1:24:15 AM   Inj : 1
Inj Volume : 10.000 µl
Method : C:\CHEM32\1\DATA\ZHIZHONG\ZG SOLUBILITY TEST 6SPOTS X3 2023-08-14 19-48-10\ZG SOLUBILITY FOR VDR HYBRIDS.M (Sequence Method)
Last changed : 14/08/2023 7:48:11 PM by SYSTEM
Additional Info : Peak(s) manually integrated

===

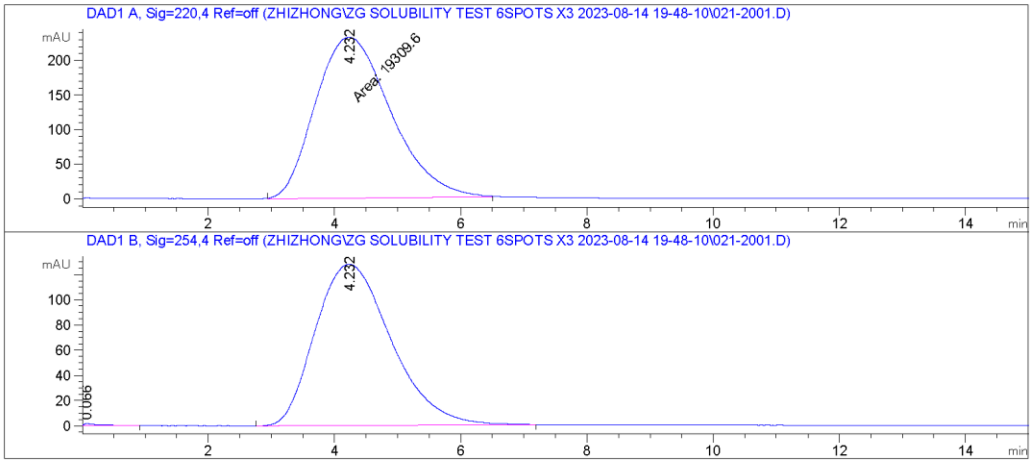

===

Fraction Information

===

No Fractions found.

===

Area Percent Report

===

Sorted By : Signal
Multiplier : 1.0000
Dilution : 1.0000
Do not use Multiplier & Dilution Factor with ISTDs

Signal 1: DAD1 A, Sig=220,4 Ref=off

Peak #	RetTime [min]	Type	Width [min]	Area [mAU*s]	Height [mAU]	Area %
1	4.232	MM	1.3838	1.93096e4	232.56323	100.0000

Totals : 1.93096e4 232.56323

===

Acq. Operator : SYSTEM   Seq. Line : 20
Acq. Instrument : 1200LC   Location : Vial 21
Injection Date : 15/08/2023 1:24:15 AM   Inj : 1
Inj Volume : 10.000 µl
Method : C:\CHEM32\1\DATA\ZHIZHONG\ZG SOLUBILITY TEST 6SPOTS X3 2023-08-14 19-48-10\ZG SOLUBILITY FOR VDR HYBRIDS.M (Sequence Method)
Last changed : 14/08/2023 7:48:11 PM by SYSTEM
Additional Info : Peak(s) manually integrated

===

Signal 2: DAD1 B, Sig=254,4 Ref=off

Peak #	RetTime [min]	Type	Width [min]	Area [mAU*s]	Height [mAU]	Area %
1	0.066	BB	0.2549	25.98524	1.41092	0.2434
2	4.232	BB	1.2901	1.06520e4	127.84653	99.7566

Totals : 1.06780e4 129.25745

===

*** End of Report ***

HPLC report of **20b** (ZG-126)

```
Data File C:\CHEM32\...HIZHONG\ZG SOLUBILITY TEST 6SPOTS X3 2023-08-14 19-48-10\012-0401.D
Sample Name: ZG-126 0.495
    =====================================================================
    Acq. Operator   : SYSTEM                          Seq. Line :   4
    Acq. Instrument : 1200LC                           Location : Vial 12
    Injection Date  : 14/08/2023 8:43:26 PM                 Inj :   1
                                                      Inj Volume : 10.000 µl
    Method          : C:\CHEM32\1\DATA\ZHIZHONG\ZG SOLUBILITY TEST 6SPOTS X3 2023-08-14 19-48-10\
                      ZG SOLUBILITY FOR VDR HYBRIDS.M (Sequence Method)
    Last changed    : 14/08/2023 7:48:11 PM by SYSTEM
    Additional Info : Peak(s) manually integrated
    =====================================================================
```

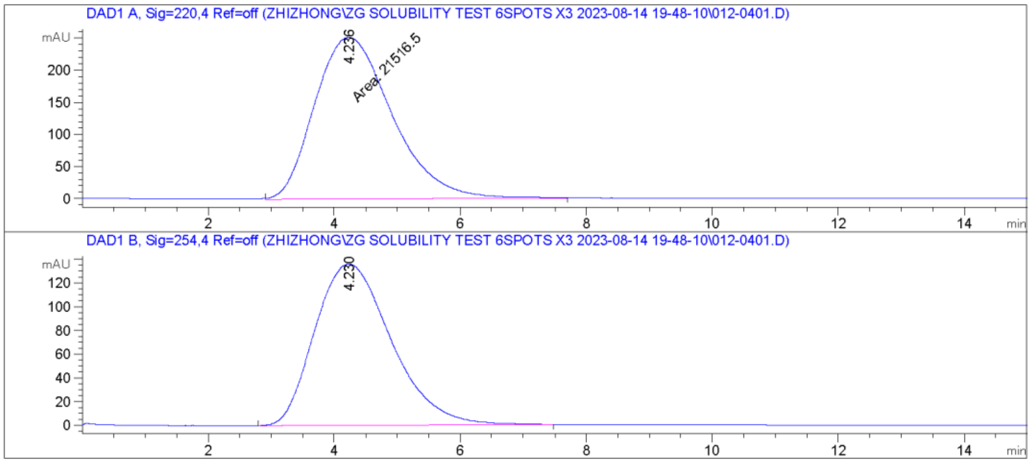


```
    =================================================================================
                               Fraction Information
    =================================================================================
    =================================================================================
    No Fractions found.
    =================================================================================
    =====================================================================
                             Area Percent Report
    =====================================================================

    Sorted By             :      Signal
    Multiplier            :      1.0000
    Dilution              :      1.0000
    Do not use Multiplier & Dilution Factor with ISTDs


    Signal 1: DAD1 A, Sig=220,4 Ref=off

    Peak RetTime Type  Width     Area      Height     Area
      #   [min]        [min]   [mAU*s]     [mAU]        %
    ----|-------|----|-------|----------|----------|--------|
       1   4.236 MM    1.4231 2.15165e4  251.99834 100.0000

    Totals :                  2.15165e4  251.99834
```

Data File C:\CHEM32\...HIZHONG\ZG SOLUBILITY TEST 6SPOTS X3 2023-08-14 19-48-10\012-0401.D
Sample Name: ZG-126 0.495

```
=====================================================================
Acq. Operator   : SYSTEM                        Seq. Line :   4
Acq. Instrument : 1200LC                         Location : Vial 12
Injection Date  : 14/08/2023 8:43:26 PM               Inj :   1
                                              Inj Volume : 10.000 µl
Method          : C:\CHEM32\1\DATA\ZHIZHONG\ZG SOLUBILITY TEST 6SPOTS X3 2023-08-14 19-48-10\
                  ZG SOLUBILITY FOR VDR HYBRIDS.M (Sequence Method)
Last changed    : 14/08/2023 7:48:11 PM by SYSTEM
Additional Info : Peak(s) manually integrated
=====================================================================
```

Signal 2: DAD1 B, Sig=254,4 Ref=off

Peak #	RetTime [min]	Type	Width [min]	Area [mAU*s]	Height [mAU]	Area %
1	4.230	BB	1.2798	1.14230e4	135.73738	100.0000

Totals : 1.14230e4 135.73738

```
=====================================================================
                          *** End of Report ***
```

HPLC report of **20c** (ZG-102)

```
Data File C:\CHEM32\1\DATA\ZHIZHONG\ZG SOLUBILITY TEST 2021-09-01 19-37-45\011-0101.D
Sample Name: ZG-102 1.0
    =====================================================================
    Acq. Operator   : SYSTEM                          Seq. Line :   1
    Acq. Instrument : 1200LC                           Location : Vial 11
    Injection Date  : 01/09/2021 7:40:32 PM                 Inj :   1
                                                    Inj Volume : 20.000 µl
    Acq. Method     : C:\CHEM32\1\DATA\ZG SOLUBILITY TEST 2021-09-01 19-37-45\ZG SOLUBILITY FOR
                      VDR HYBRIDS.M
    Last changed    : 01/09/2021 7:37:45 PM by SYSTEM
    Analysis Method : C:\CHEM32\1\DATA\ZHIZHONG\ZG SOLUBILITY TEST 2021-09-01 19-37-45\ZG
                      SOLUBILITY FOR VDR HYBRIDS.M (Sequence Method)
    Last changed    : 01/09/2021 7:37:45 PM by SYSTEM
    Additional Info : Peak(s) manually integrated
    =====================================================================
```

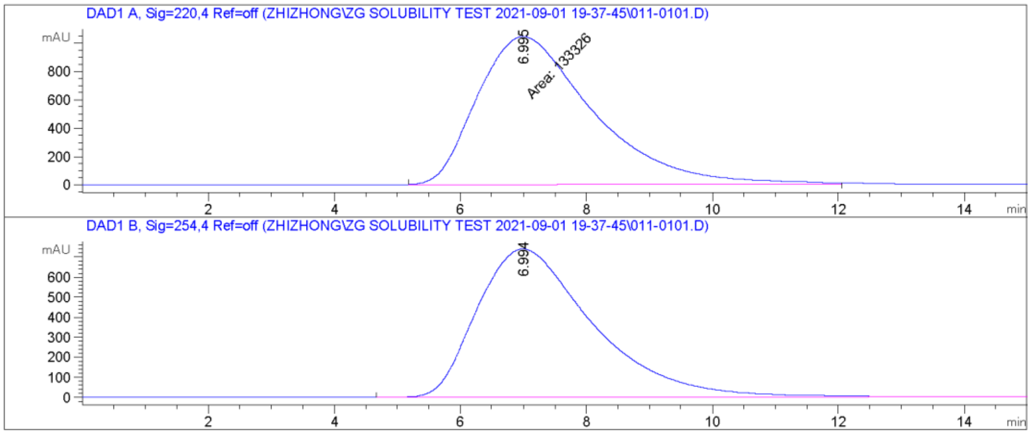


```
    =================================================================================
                              Fraction Information
    =================================================================================
    =================================================================================
    No Fractions found.
    =================================================================================
    =====================================================================
                            Area Percent Report
    =====================================================================

    Sorted By             :      Signal
    Multiplier            :      1.0000
    Dilution              :      1.0000
    Sample Amount:               :      1.00000  [ng/ul]   (not used in calc.)
    Do not use Multiplier & Dilution Factor with ISTDs


    Signal 1: DAD1 A, Sig=220,4 Ref=off

    Peak RetTime Type  Width     Area      Height     Area
      #   [min]        [min]   [mAU*s]     [mAU]        %
    ----|-------|----|-------|----------|----------|--------|
       1   6.995 MM    2.1334 1.33326e5  1041.56934 100.0000

    Totals :                  1.33326e5  1041.56934

1200LC 23/08/2023 12:35:56 PM SYSTEM                                          Page    1 of 2
```

Data File C:\CHEM32\1\DATA\ZHIZHONG\ZG SOLUBILITY TEST 2021-09-01 19-37-45\011-0101.D
Sample Name: ZG-102 1.0

===

Acq. Operator : SYSTEM Seq. Line : 1
Acq. Instrument : 1200LC Location : Vial 11
Injection Date : 01/09/2021 7:40:32 PM Inj : 1
Inj Volume : 20.000 µl
Acq. Method : C:\CHEM32\1\DATA\ZG SOLUBILITY TEST 2021-09-01 19-37-45\ZG SOLUBILITY FOR VDR HYBRIDS.M
Last changed : 01/09/2021 7:37:45 PM by SYSTEM
Analysis Method : C:\CHEM32\1\DATA\ZHIZHONG\ZG SOLUBILITY TEST 2021-09-01 19-37-45\ZG SOLUBILITY FOR VDR HYBRIDS.M (Sequence Method)
Last changed : 01/09/2021 7:37:45 PM by SYSTEM
Additional Info : Peak(s) manually integrated

===

Signal 2: DAD1 B, Sig=254,4 Ref=off

Peak #	RetTime [min]	Type	Width [min]	Area [mAU*s]	Height [mAU]	Area %
1	6.994	BBA	1.9306	9.48711e4	739.58521	100.0000
Totals :				9.48711e4	739.58521	

===

*** End of Report ***

Dose response profiles for inhibition of different HDACs by ZG-126.

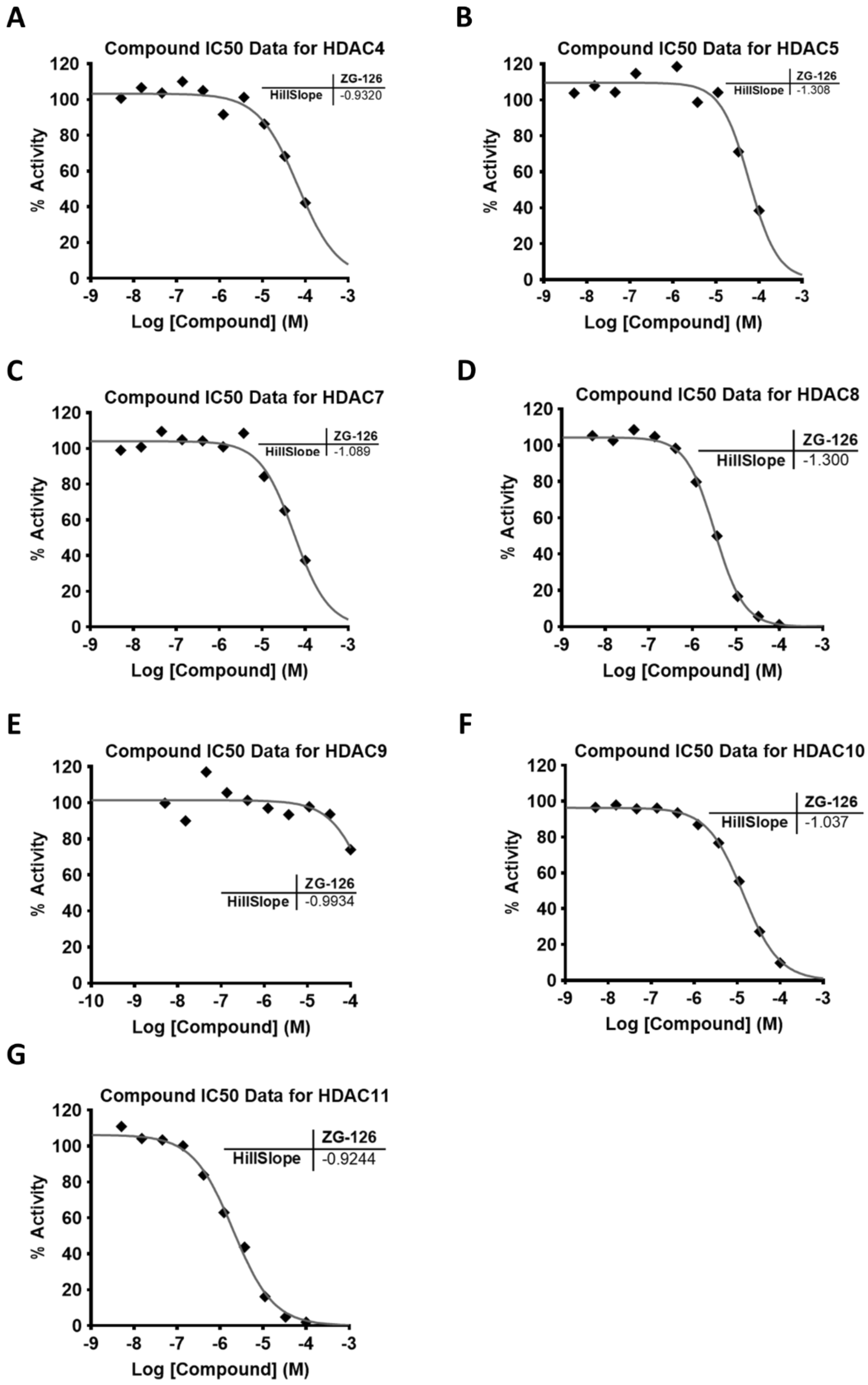

Figure S1. Dose response profiles for inhibition of HDAC4 **(A),** HDAC5 **(B),** HDAC7 **(C),** HDAC8 **(D)** HDAC9 **(E),** HDAC10 **(F)** and HDAC11 **(G)** by ZG-126.

Bifunctionality of ZG-126 in mouse melanoma B16-F10 cells *in vitro*.

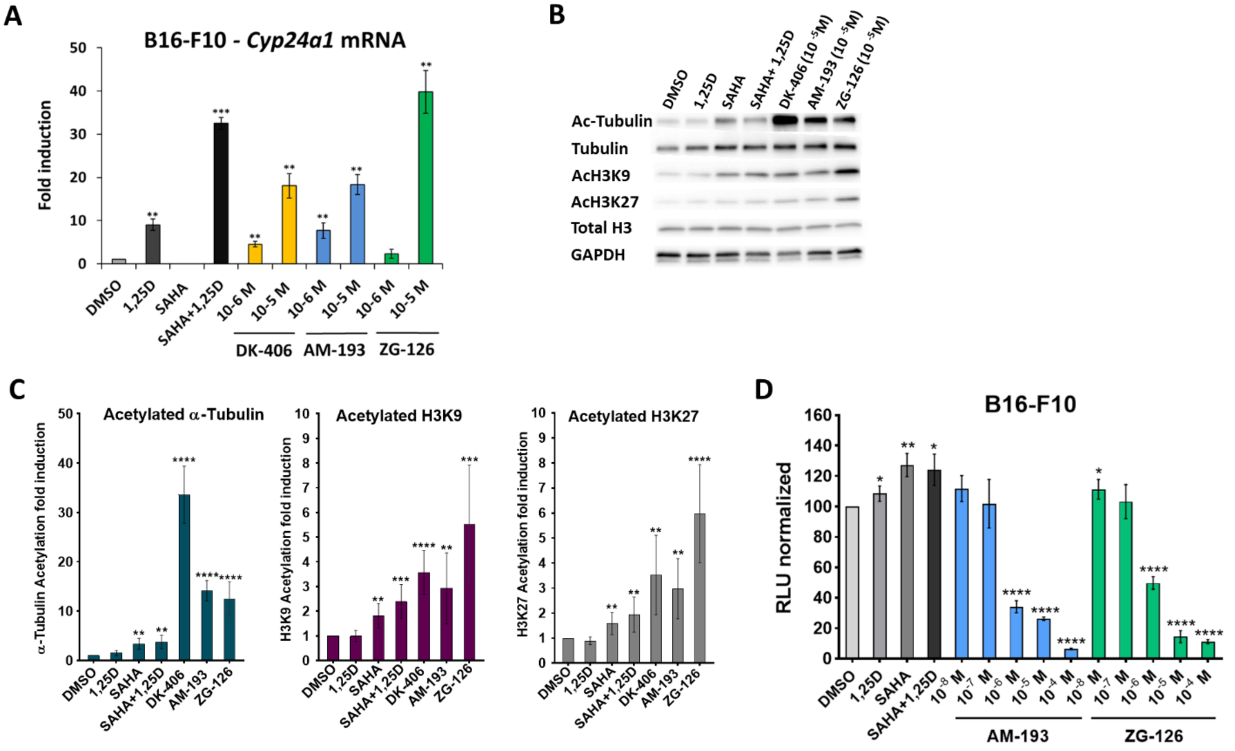

Figure S2. A. Analysis of *Cyp24a1* induction by ZG126, DK406, AM193 along with 1,25D and SAHA as controls in B16-F10 cells. **B.** Western blots analyzing the effects of 1,25D, SAHA and hybrids on hyperacetylation of tubulin, H3K9 and H3K27 in B16-F10 cells. **C.** Quantification of the analyses of hyperacetylation in B16-F10 cells of tubulin, H3K9 and H3K27. **D.** Assessment of cytotoxicity in B16-F10 cells by Glo MT assay.

Effect of ZG-126 on recruitment and polarization of macrophages in 4T1 tumors

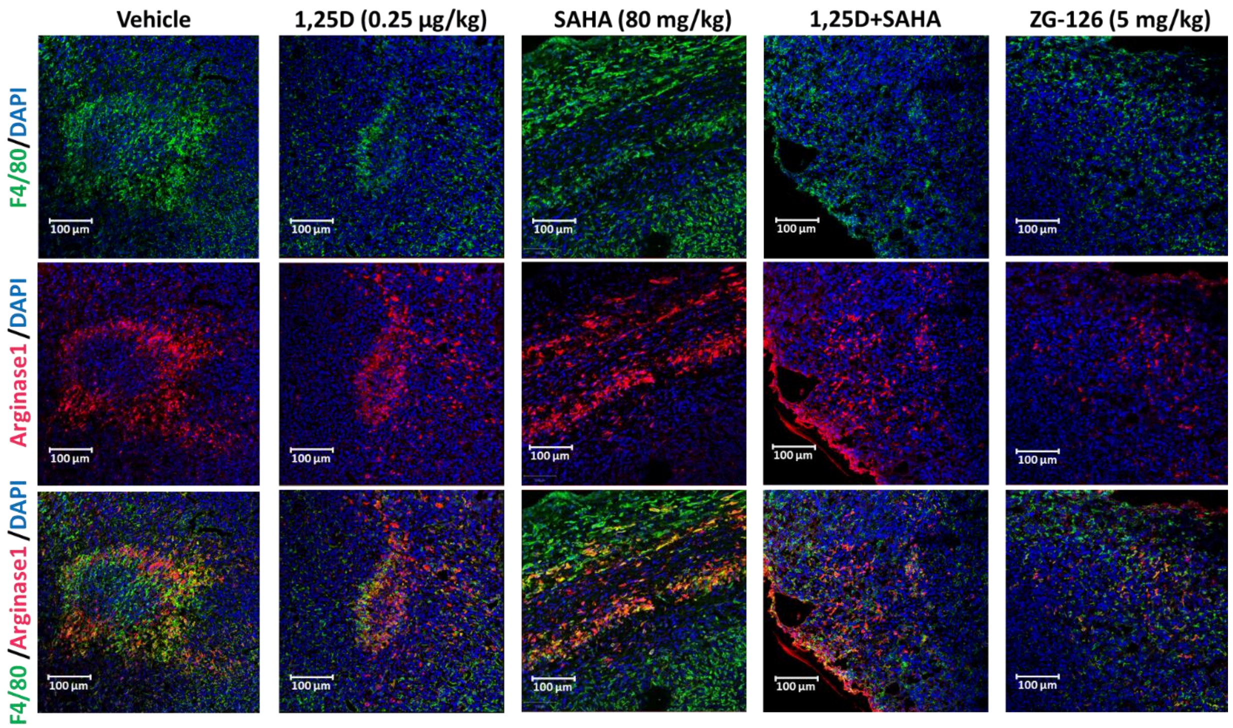

Figure S3. Immunofluorescence analysis of 4T1 tumors stained for macrophage marker, F4/80 and M2 macrophage-specific marker, Arg1. Tumors were from animals treated with vehicle, 1,25D, SAHA, 1,25D+SAHA and high dose ZG-126, as indicated.

Table S1. Hybrid molecules IC50s extracted from the *in vitro* cell viability assay.

Cell line / Compound	4T1	MDA-MB-231
**DK-406**	9.08 μM	2.43 μM
**AM-193**	0.74 μM	0.5 μM
**ZG-102**	55.8 μM	2.18 μM
**ZG-126**	0.89 μM	0.92 μM
**ZG-132**	4.55 μM	0.95 μM

Table S2. Primer sequences for RT-qPCR

Target gene	Forward Sequence	Forward Sequence
Cyp24a1	5'-GAAGCTGTGAACTTCATCAT-3'	5'-CTGCACTAGGCTGCTGAGAA-3'
Ccl2	5'-AGGTCCCTGTCATGCTTCTG-3'	5'-TCTCTTGAGCTTGGTGACAAAA-3'
Ccl5	5'-AGCAGCAAGTGCTCCAATCT-3'	5'-CTTGAACCCACTTCTTCTCTGG-3'
Ccl20	5'-ACATACAGACGCCTCTTCCTTC-3'	5'-GCGCACACAGATTTTCTTTTCT -3'
Cxcl10	5'-GCCATAGGGAAGCTTGAAATC-3'	5'-TCAGACATCTCTGCTCATCATTC-3'
Il1α	5'-TCAAGCAACGGGAAGATTCTGA -3'	5'-CTCTGGTAGGTGTAAGGTGCTG-3'
Vdr	5'-ACGCTATGACCTGTGAAGGC-3'	5'-TTACGCTGCACCTCCTCATC-3'
18s	5'-GCAATTATTCCCCATGAACG-3'	5'-GGGACTTAATCAACGCAAGC-3'
